# Microfluidic Formulation of Topological Hydrogels for Microtissue Engineering

**DOI:** 10.1021/acs.chemrev.1c00798

**Published:** 2022-09-15

**Authors:** Katarzyna O. Rojek, Monika Ćwiklińska, Julia Kuczak, Jan Guzowski

**Affiliations:** Institute of Physical Chemistry, Polish Academy of Sciences, ul. Kasprzaka 44/52, 01-224 Warsaw, Poland

## Abstract

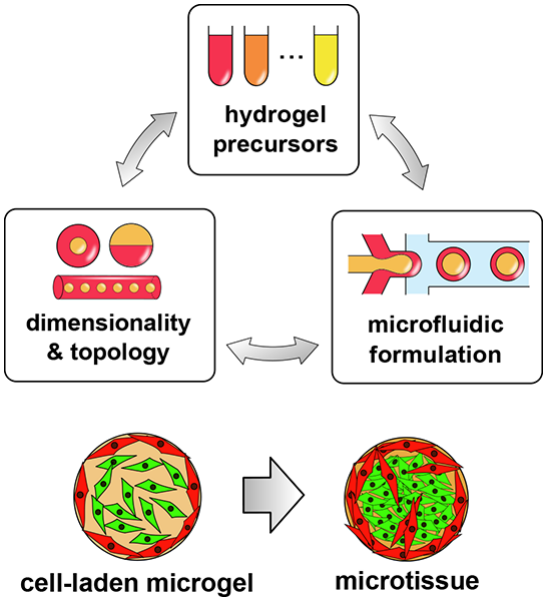

Microfluidics has recently emerged as a powerful tool in generation of submillimeter-sized cell aggregates capable of performing tissue-specific functions, so-called microtissues, for applications in drug testing, regenerative medicine, and cell therapies. In this work, we review the most recent advances in the field, with particular focus on the formulation of cell-encapsulating microgels of small “dimensionalities”: “0D” (particles), “1D” (fibers), “2D” (sheets), etc., and with nontrivial internal topologies, typically consisting of multiple compartments loaded with different types of cells and/or biopolymers. Such structures, which we refer to as topological hydrogels or topological microgels (examples including core–shell or Janus microbeads and microfibers, hollow or porous microstructures, or granular hydrogels) can be precisely tailored with high reproducibility and throughput by using microfluidics and used to provide controlled “initial conditions” for cell proliferation and maturation into functional tissue-like microstructures. Microfluidic methods of formulation of topological biomaterials have enabled significant progress in engineering of miniature tissues and organs, such as pancreas, liver, muscle, bone, heart, neural tissue, or vasculature, as well as in fabrication of tailored microenvironments for stem-cell expansion and differentiation, or in cancer modeling, including generation of vascularized tumors for personalized drug testing. We review the available microfluidic fabrication methods by exploiting various cross-linking mechanisms and various routes toward compartmentalization and critically discuss the available tissue-specific applications. Finally, we list the remaining challenges such as simplification of the microfluidic workflow for its widespread use in biomedical research, bench-to-bedside transition including production upscaling, further in vivo validation, generation of more precise organ-like models, as well as incorporation of induced pluripotent stem cells as a step toward clinical applications.

## 1 Introduction

Conventional “2D” cell cultures, relying on the use of a monolayer of cells cultured at a bottom of a culture flask, have been a standard in biology research, vaccine production, and drug testing for over a century. However, interaction of cells with flat, stiff, typically plastic substrates in general leads to nonphysiological cell responses and results in cell phenotypes which do not reproduce those encountered in vivo.^1^ To provide a more physiological microenvironment, in particular facilitating three-dimensional arrangement of cells and/or providing a three-dimensional (“3D”) support mimicking the extracellular matrix (ECM) of the native tissue,^2^ the so-called “3D” cell culture techniques have been developed.^1^ Those techniques can be in general categorized into those relying (i) on the use of nonadhesive substrates promoting cell–cell interactions and resulting in aggregation of cells into spheroids without an external hydrogel support, or (ii) on embedding the cells within the ECM-like hydrogel matrix, which provides the external support and leads to more physiological cell and tissue morphologies. The most recent developments in the 3D cell culture succeeded in integration of the two approaches via the use of microscopic hydrogel (microgel) scaffolds capable of providing both a controlled degree of confinement as well as highly biomimetic local 3D microenvironment,^3−6^ allowing for generation of reproducible, yet biologically relevant microtissues.

The areas of particularly rapid technological development in 3D cell culture include new strategies of formulation of biomaterials at the scale typical of cell aggregates or tissues at the early stages of development, i.e., at the scale of the order of 100–1000 um. Such mesoscale biomaterials could serve as scaffolds for production of microtissues in vitro, i.e., cell aggregates capable of performing basic physiological functions typical of a given tissue. Besides basic tissue-biological research, microtissues could also serve high-throughput drug testing, as microscopic living tissue “probes”, to complement or eventually replace animal models. Further, custom-tailored microtissues, generated with high reproducibility and throughput, could also be used as building blocks of more complex living constructs. The general strategy of the latter “modular” approach to tissue engineering consists of arranging the distinct hydrogel compartments, loaded with different types of cells, into a biomimetic 3D scaffold.^3,4^ Such structures provide well-defined “initial conditions” for tissue maturation, that is, for cell proliferation and differentiation into a functional tissue. Importantly, compartmentalization allows not only for 3D cell patterning^7^ but also 3D biopolymer patterning,^4^ where the latter can be used to impose varying physicochemical cues including local gradients in matrix stiffness and/or molecular protein or peptide content. In particular, the composition of the matrix can be engineered to locally promote or suppress cell–matrix interaction and thus control morphology of the ensuing microtissues. In terms of applications, the recent advancements in hydrogel microfabrication methods^8−17^ have opened new perspectives in regenerative medicine,^18,19^ personalized drug testing,^20−22^ as well as in basic cell- and tissue physiology research,^2,21^ including cancer research.^23−28^

In this review, we focus on the most recent developments in microfluidics-assisted formulation of biomaterials, in particular on those with nontrivial internal architecture, typically consisting of multiple distinct compartments.^8,16−18,29,30^ We use the term “microfluidics” to describe a set of techniques aimed at developing of high-level of control over tiny liquid volumes, typically nano- or even picoliter volumes, at submillimeter length scale. In particular, microfluidics can be used to disperse hydrogel precursor solutions into monodisperse droplets or extrude them into stable jets, which subsequently solidify, either spontaneously or via externally triggered cross-linking reaction, into hydrogel microparticles,^8,9,13,14,18,31−35^ microfibers,^36−40^ or more general “microgels”. The laminar (nonturbulent) flow conditions associated with small dimensions of the microchannels lead to reproducibility of droplet and jet morphologies as well as facilitate precise manipulation of the microscopic hydrogel liquid compartments, e.g., their on-chip merging or splitting.^41^ In particular, controlled coalescence of microfluidic droplets or jets containing different hydrogel precursors allows reproducible generation of compartmentalized hydrogel microstructures. Microfluidics can be used to formulate compartmentalized hydrogels of various “dimensionalities”^15^ ranging from “0D” particles and “1D” fibers to “1.5D” ribbons. Furthermore, the particles or fibers can be assembled into larger architectures^11,42^ such as granular, porous, or woven “2D” sheets or even densely packed, stacked, or bundled granular “3D” architectures.^43^ It is noteworthy that granular hydrogels can also be used as injectable biomaterials for tissue regeneration, wound healing, or drug delivery.^34,43^

Some of the most common examples of the internally compartmentalized microgels include “0D” core–shell structures (microcapsules)^32,44^ with a soft core and a rigid shell, where the shell provides a physical barrier, protecting the cells against the external disturbing factors such as shear forces or interactions with cells outside of the microstructure. The confined microenvironment additionally expedites cell aggregation and, in many cases, promotes cell differentiation, which in turn facilitates the development of tissue-specific functions. In bioreactor cultures involving multiple microtissues, the presence of protective shells prevents excessive cell aggregation in the cores and as such limits the risk of hypoxia. Core–shell “1D” structures (microfibers) can also serve as scaffolds for cell expansion.^40^ In addition, the elongated morphology can be exploited in culturing of tissues of fiber-like morphology such as muscles or nerves.

Overall, the large-scale production of tailorable “0D” or “1D” microgels opens new perspectives in tissue engineering and regenerative medicine, in particular in restoration of tissues such as muscle,^45−47^ heart,^47,48^ bone,^49^ or neural tissue^50^ or in cell-based therapies for treatment of diseases such as diabetes^51^ or infertility.^52^

Recently, microfluidic technologies have also been applied to culture macroscopic amounts of cells for the use as food products, e.g., cultured meat.^53^

As a complement to the already available large body of literature considering microfluidic formulation of microgels, in this review, we focus on classifying various possible routes toward their compartmentalization and self-assembly, including generation of structures of different topologies and dimensionalities. In particular, we establish that the available microfluidic techniques of formulation of compartmentalized architectures exploit either (i) the self-assembled *equilibrium* liquid architectures, typically consisting of multiple *immiscible* liquid segment, or (ii) transient *nonequilibrium* architectures consisting of multiple *miscible* segments quenched via rapid cross-linking reactions. In addition, we systematically review the most recent tissue-specific applications of the topological microstructures encompassing multiple types of microtissues, including miniature pancreas, liver, muscle, bone, heart, neural tissue, vasculature, as well as stem cell spheroids and microtumors. In each case, we highlight biological relevance of microcompartmentalization and its role in providing the optimal conditions for tissue maturation as signified by cell differentiation and/or secretion of tissue-specific markers.

**Figure 1:**
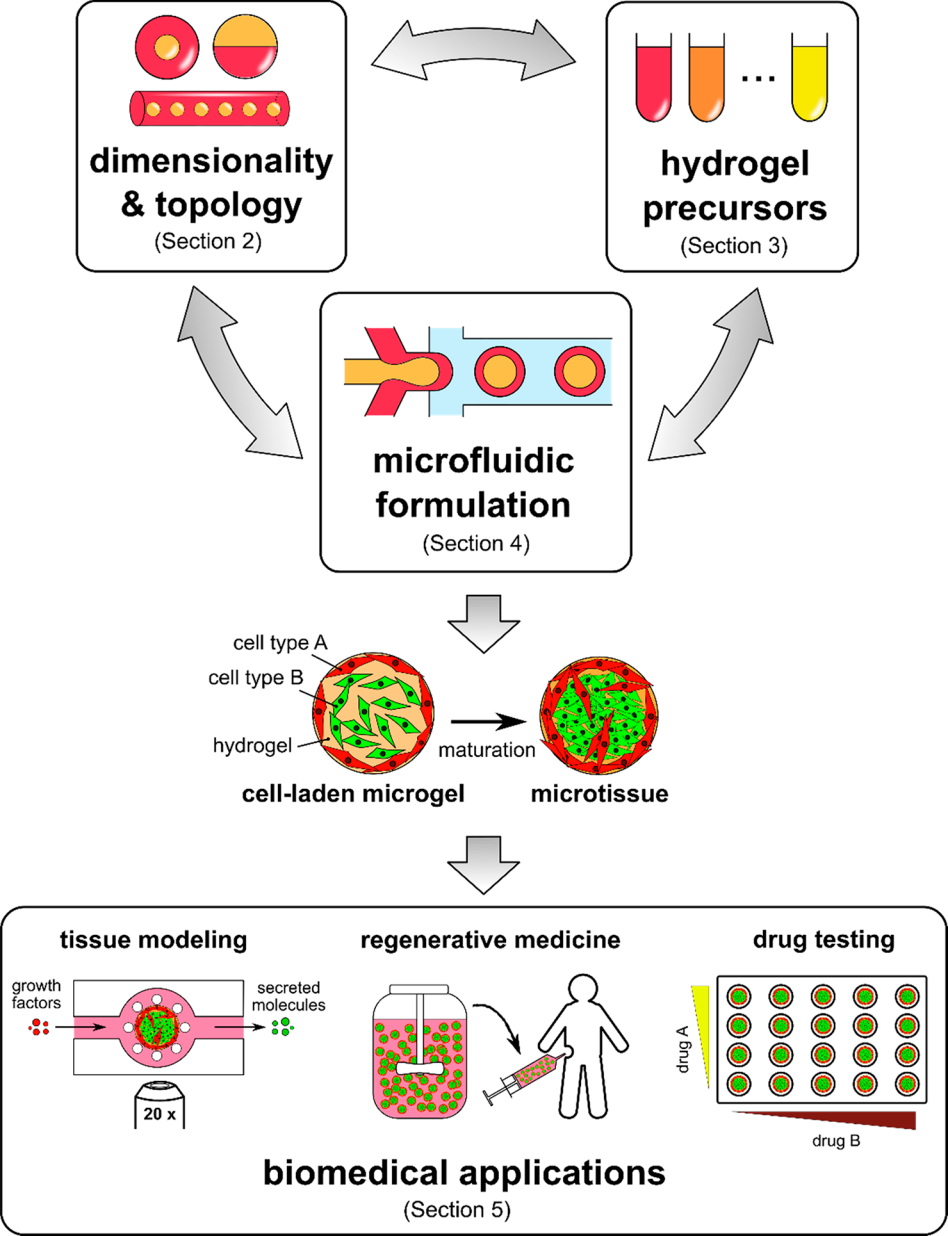
Main subjects of interest in microfluidics-assisted microtissue engineering (with indicated corresponding section numbers in this review): (i) experimentally achievable microgel topologies and dimensionalities, (ii) properties of different hydrogel biomaterials, (iii) microfluidic formulation strategies, and their (iv) biomedical applications. Note that the desired final topology of a microgel and the choice of the type of the hydrogel often dictate the choice of a particular formulation strategy. The generated topological structures with embedded cells, so-called microtissues, may serve as (i) reproducible in vitro tissue models, (ii) cell sources for tissue regeneration and cell therapies, or (iii) as tissue “probes” for high-throughput drug testing.

Our paper is structured as follows. We start with a general classification of the available microgel topologies and dimensionalities in section 2, followed by a short review of the different types of hydrogels used in microfluidic formulations in section 3. Next, we turn to a detailed description of the available microfluidic methods of fabrication of the topological hydrogel microstructures in section 4 and the different types of biomedical applications in section 5, including a detailed review of most recent tissue-specific applications in section 5.2. Finally, we discuss the remaining challenges and the emerging commercial microtissue-based applications in section 6. For convenience of the reader, the flow of information is also schematically displayed in Figure 1.

## 2 General Classification of Microgel Dimensionalities and Topologies

**Figure 2:**
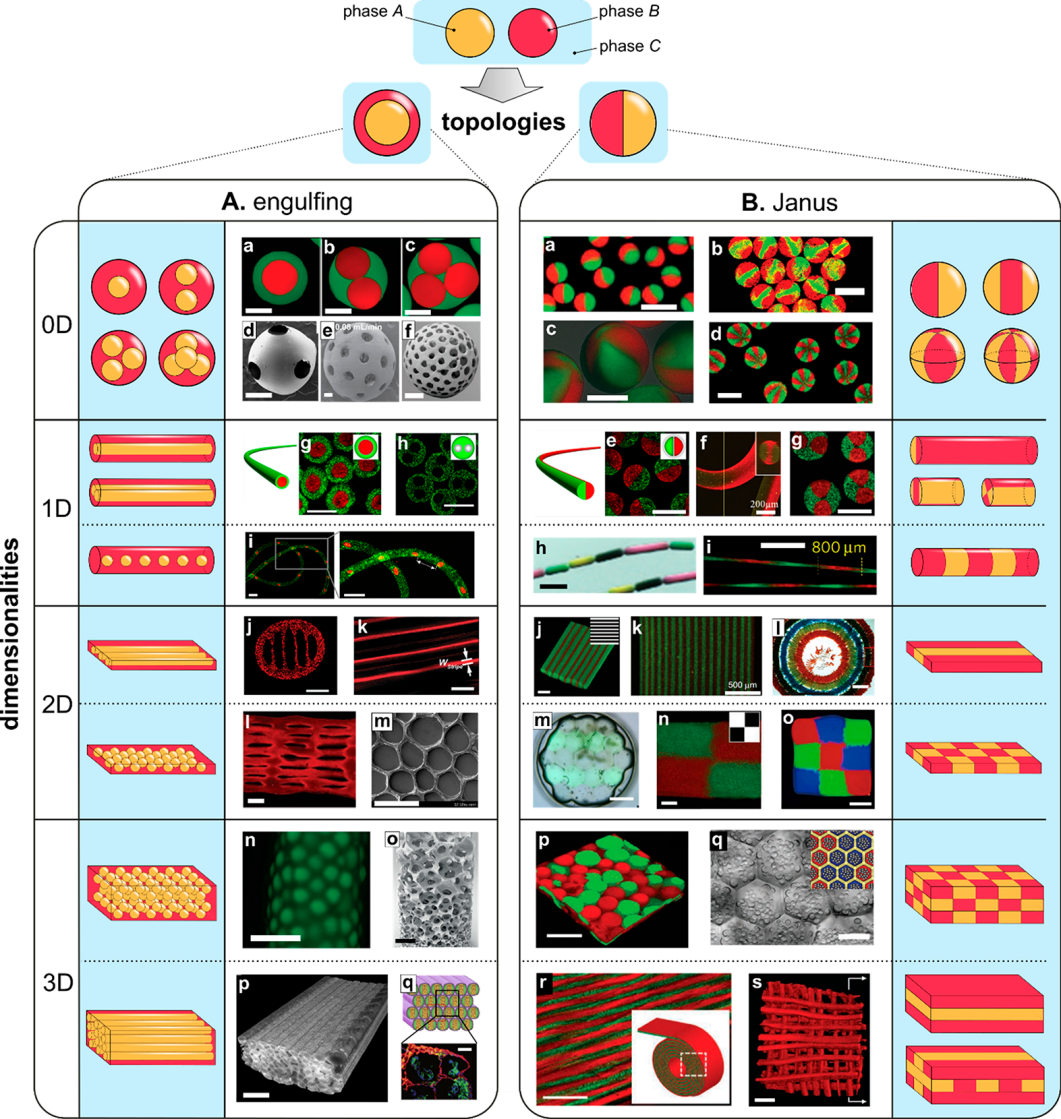
The variety of microgel structures for *N* = 2. We classify the structures in terms of overall topology (“engulfing” vs “Janus”) and dimensionality (0D, 1D, 2D, 3D) for the case of two (*N* = 2) different hydrogel phases A and B (or a hydrogel and another immiscible phase such as oil or gas) suspended in the external phase C (typically cell culture medium). (A) (a–c) pNIPAAM core–shell beads. Scale bars 100 μm. Adapted with permission from ref (63). Copyright 2010 American Chemical Society. (d–f) ETPTA, gelatin, and alginate–chitosan porous beads, respectively. Scale bars 200 μm. Adapted with permission from refs (64−66), respectively. Copyright 2015 American Chemical Society, 2013 Wiley, and 2018 Wiley, respectively. (g–h) Cross-sectional view of alginate core–shell fibers and hollow fibers, respectively. Scale bars 200 μm. Adapted with permission from ref (67). Copyright 2018 Nature Publishing Group. (i) Alginate fibers engulfing aqueous droplets. Scale bar 300 μm. Adapted with permission from ref (68). Copyright 2021 American Chemical Society. (j) Cross-section of an alginate ribbon with multiple hollow cores. Scale bar 100 μm. Adapted with permission from ref (69). Copyright 2016 Wiley. (k) Alginate sheet. Scale bar 2 mm. Adapted with permission from ref (70). Copyright 2018 Royal Society of Chemistry. (l) Fluorescent alginate sheet. Scale bar 500 μm. Adapted with permission from ref (59). Copyright 2012 Wiley. (m) Porous alginate membrane. Scale bar 100 μm. Adapted with permission from ref (71). Copyright 2016 American Chemical Society. (n) Close-packed norbornene-modified hyaluronic acid (NorHA) microbeads suspended in PBS. Scale bar 200 μm. Adapted with permission from ref (72). Copyright 2019 Wiley. (o) Porous gelatin scaffold with gradient in pore size. Scale bar 500 μm. Adapted with permission from ref (54). Copyright 2019 Wiley. (p) Extrusion 3D-printed alginate fibers. Scale bar 500 μm. Adapted with permission from ref (73). Copyright 2017 Elsevier. (q) Cross-section of wet-spun cell-laden alginate fibers. Scale bar 50 μm. Adapted with permission from ref (74). Copyright 2018 Elsevier. (B) (a–d) Alginate beads. Scale bars 500, 100, 200, and 200 μm, respectively. Adapted with permission from refs (75−78), respectively. Copyright 2015 Royal Society of Chemistry, 2018 Wiley, 2013 American Institute of Physics, and 2020 Wiley, respectively. (e–g) Alginate Janus fibers. Scale bars 200 μm. Adapted with permission from ref (79 and 80). Copyright 2020 Wiley and 2014 Wiley. (h) GelMa fiber. Scale bar 1 mm. Adapted with permission from ref (81). Copyright 2019 The Royal Society of Chemistry. (i) Alginate fiber. Scale bar 800 μm. Adapted with permission from ref (82). Copyright 2011 Nature Publishing Group. (j,k) Alginate sheets. Scale bars 500 μm. Adapted with permission from refs (59 and 83), respectively. Copyright 2012 Wiley and 2013 Elsevier, respectively. (l) Granular sheet made of GelMa microrods arranged into a macroscale stripe pattern. Scale bar 2 mm. Adapted with permission from ref (84). Copyright 2017 Wiley. (m) pNIPAAM granular sheet. Scale bar 500 μm. Adapted with permission from ref (85). Copyright 2020 Nature. (n,o) Aginate and PEGDA sheets, respectively. Scale bars 500 μm. Adapted with permission from refs (59, 86, and 87), respectively. Copyright 2012 Wiley and 2016 American Association for the Advancement of Science. (p) 3D scaffold made of annealed PEG beads. Scale bar 200 μm. Adapted with permission from ref (50). Copyright 2019 Wiley. (q) 3D-printed hydrogel droplets stabilized by lipids. Scale bar 100 μm. Adapted with permission from ref (88). Copyright 2021 The Authors. (r) Rolled alginate sheet. Scale bar 500 μm. Adapted with permission from ref (59). Copyright 2012 Wiley. (s) Stacked alginate sheets. Scale bar 500 μm. Adapted with permission from ref (59). Copyright 2012 Wiley.

Rapid progress in microfluidics-assisted fabrication of microgels in the last couple of years has brought a rich variety of the available hydrogel microstructures. In this section, we provide an overview of the different hydrogel microarchitectures achievable using microfluidics. Fabrication of such structures (Figure 2) exploits a variety of flow patterns and cross-linking strategies in accordance with the type of biopolymers employed. We start with discussing the basic topological constraints which yield the physically possible architectures without going into details of their microfabrication processes, which we later discuss in section 3.

We classify the compartmentalized hydrogel microstructures according to their (i) “dimensionality” and (ii) topology. By “dimensionality”, we understand the overall shape of a microstructure determined by its dimensions in all directions *D*_*x*_, *D*_*y*_, and *D*_*z*_ relative to the size *a* of a single compartment.^15,17^ Accordingly, we may distinguish (i) **“0D”** architectures,^8,13,14,18,32^ i.e., compact structures, strongly confined in all directions, i.e., with *D*_*x*_ ∼ *D*_*y*_ ∼ *D*_*z*_ ∼ *a* (Figure 2A(a–f),B(a–d)). (ii) **“1D”** architectures,^36,37,39,54,55^ structures elongated in one direction, i.e., with *D*_*z*_ ≫ *a*, and *D*_*x*_ ∼ *D*_*y*_ ∼ *a* (Figure 2A(g–i),B(e–i)). (iii) **“2D”** architectures, i.e., planar structures with *D*_*y*_ ≫ *a*, *D*_*z*_ ≫ *a* and *D*_*x*_ ∼ *a* (Figure 2A(j–m),B(j–o)), as well as (iv) **“3D”** architectures, i.e., bulk structures^4,43^ with *D*_*x*_ ≫ *a*, *D*_*y*_ ≫ *a*, and *D*_*z*_ ≫ *a* (Figure 2A(n–q),B(p–s)). By topology of the structures we understand the type of arrangement of the compartments, in particular, their connectivity. For the purpose of our classification, we employ an analogy to multiple emulsions, i.e., to the case of droplets built of multiple immiscible liquid segments.^56^ In the case with *N* = 2 liquid or hydrogel compartments, say A and B, suspended in the third external fluid phase, C (typically cell culture medium or oil), there are in general two different possible topologies that can form.^56−58^ One can distinguish (i) *the engulfing topology*, A/B/C, in which phase B completely engulfs phase A, such that only phase B has a direct contact with the external phase C (Figure 2A(a,g) and (ii) *the Janus topology*, (A–B)/C, in which both phases have a direct contact with the external phase as well as with each other (Figure 2B(a,e)). We note that the type of topology does not necessarily determine the dimensionality of the structure (nor vice versa): both the engulfing and the Janus topologies can be realized either in the case of “0D” (Figure 2A(a–f),B(a–d)) as well as “1D” structures (Figure 2A(g–i),B(e–i)) or higher-dimensional “2D” and “3D” structures (Figure 2A(j–q),B(j–s)). The complexity of the structures rapidly increases upon increasing the numbers *m*_A_ or *m*_B_ of compartments of the types A or B, respectively. In such a way, one can achieve, for example, the engulfing core–shell topologies with multiple cores in a single shell, (A_1_, . . .,A_*m*_A__)/B/C (Figure 2A(b–f),(h–q)) or multi-Janus topologies (A_1_ – B_1_ – ... – A_*m*_A__ – B_*m*_B__)/C (Figure 2B(b–d),(f–s)).

It is noteworthy that, in the case “1D”, besides the overall topology of the structure, one can also distinguish two different types of the internal patterning: either transversal, with compartments arranged across the fiber, or longitudinal, with compartments arranged along the fiber. In the former case, the pattern is translationally invariant along the fiber. On the contrary, in the latter case, the cross-section varies according to the longitudinal distribution of the compartments. Interestingly, this latter type of structures can be conveniently used to encode information.^59,60^ Actually, the most efficient information coders seem to be the “1.5D” ribbon-like structures which can be patterned in both longitudinal and transverse directions;^59^ see also section 4.5.4. Such coded fibers or ribbons could be used to “label” multiple microtissues sequentially embedded in the structure for the purpose of their identification, e.g., in a high-throughput screening assay.

**Figure 3:**
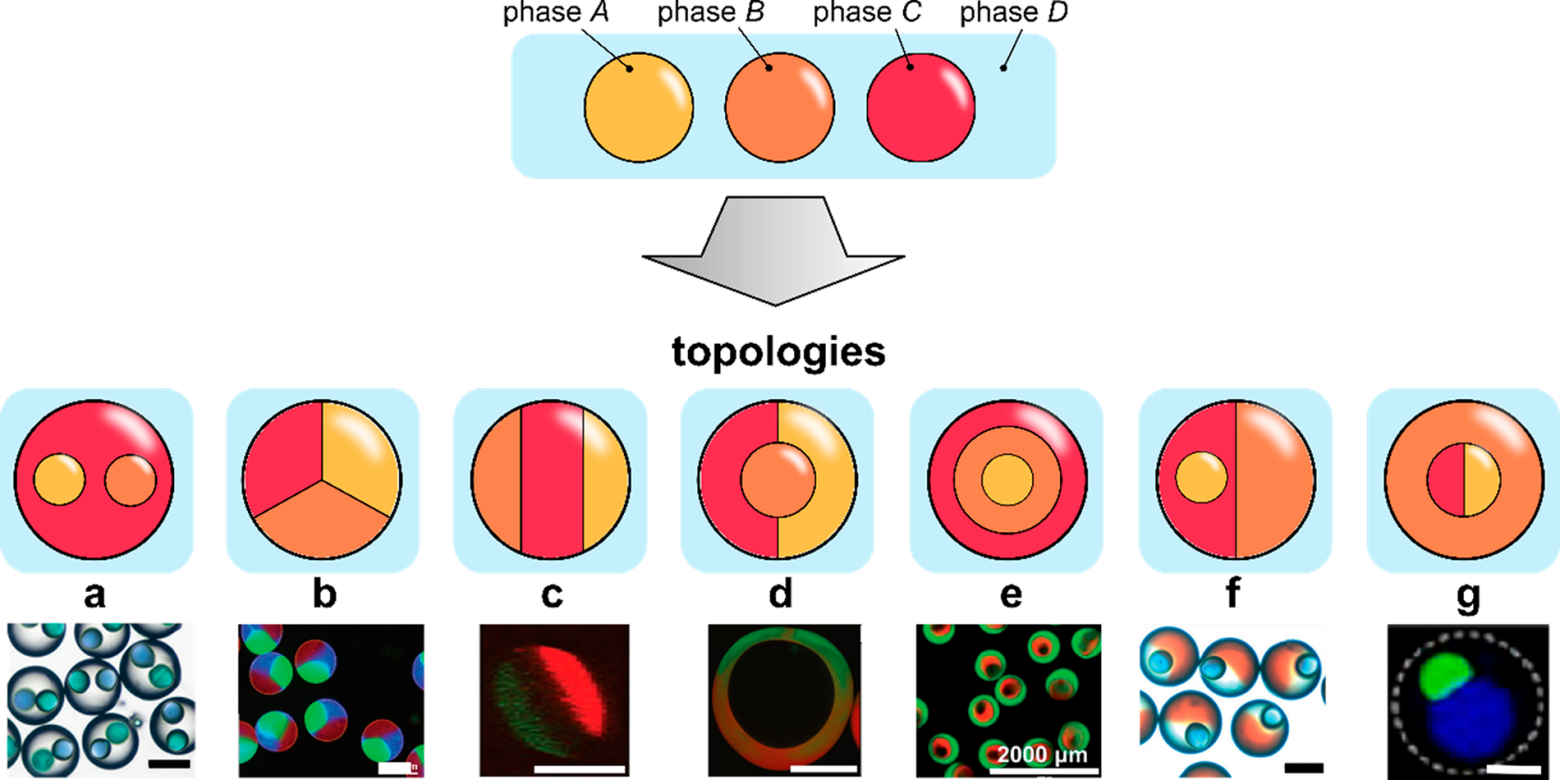
A complete list of distinct topologies for *N* = 3. (a) PVA cores in trimethylolpropane triacrylate (TMPTA) shell. Scale bar 200 μm. Adapted with permission from ref (89). Copyright 2017 Royal Chemical Society. (b) Alginate beads. Scale bar 100 μm. Adapted with permission from ref (62). Copyright 2012 Wiley. (c) Cross-section of an alginate fiber with three compartments. Scale bar 200 μm. Adapted with permission from ref (80). Copyright 2020 The Authors. (d) Oil core in a two-compartment alginate shell. Scale bar 100 μm. Adapted with permission from ref (90). Copyright 2010 American Chemical Society. (e) Alginate core–shell–shell beads. Scale bar 2 mm. Adapted with permission from ref (75). Copyright 2015 Royal Society of Chemistry. (f) Janus trimethylolpropane triacrylate (TMPTA) bead with embedded single oil core. Scale bar 200 μm. Adapted with permission from ref (89). Copyright 2017 Royal Chemical Society. (g) Janus-in-shell morphology obtained using aqueous three-phase droplet (dextran, PEG, PVA) suspended in an external oil phase. Scale bar 100 μm. Adapted with permission from ref (91). Copyright 2017 American Chemical Society.

To conclude the topological considerations, we note that, in general, the number *n* of available topologies rapidly grows with the number of compartments *m* as well as with the number of different hydrogel species *N* (in particular, one must have *m* ≥ *N*). In the simplest case *m* = *N*, for *N* = 2, we have *n* = 2 basic topologies (“engulfing” and “Janus”), while in the case *N* = 3, the number of such basic topologies increases to *n* = 7, all of them explicitly listed in Figure 3 (as demonstrated recently,^61^ in multiphase liquid architectures *n* can be calculated based on graph-theoretical considerations). In fact, all of those topologies have been already experimentally demonstrated using microfluidics. At higher *N*, the available topologies have not been much explored, with some exceptions.^62^ In general, it is clear that with increasing *N*, the complexity of the available microstructures rapidly becomes insurmountable. Apparently, the case *N* = 2 represents a reasonable trade-off between complexity and experimental feasibility and, indeed, the most of the available demonstrations involve two different types of hydrogel compartments. In fact, the most recent research efforts tend to focus mostly on developing control over the relative spatial arrangement of the compartments and on tuning of their physicochemical properties rather than on further increasing *N*.

## 3 Hydrogels as ECM-Mimics in Microtissue Engineering

Hydrogels consist of a network of cross-linked hydrophilic polymer chains. Due to their 3D mesh-like nanostructure, hydrogels are capable of absorbing large amounts of water, a feature which, together with other properties, such as biocompatibility, biodegradability, nanoporosity, and adjustable mechanical properties, make them perfect candidates for tissue-engineering applications. To properly mimic the extracellular matrix of a given tissue, the mechanical and biochemical properties of a hydrogel, such as the Young’s modulus or the presence of molecular motifs promoting cell adhesion, e.g., arginylglycylaspartic acid, the so-called RGD peptide motif, need to be carefully adjusted considering the specific type of cultured cells. In the choice of the hydrogel (and its cross-linking method), it should be taken into account that some of the hydrogels (e.g., polyacrylamides) or chemical cross-linkers (e.g., glutaraldehyde) are cytotoxic,^92^ which strongly limits their applicability in biomaterial formulation. Also, it should be considered that, e.g., free radicals generated during UV-triggered cross-linking, as well as UV light itself, may cause cell damage.^93^ Biopolymers most commonly used in preparation of microgels for tissue engineering include (i) those of natural origin such as polysaccharides, e.g., agarose, hyaluronic acid, chitosan or calcium alginate, or protein-based such as gelatin, collagen, fibrin, or Matrigel or other types of decellularized matrices (dECM), (ii) partially synthetic ones such as gelatin methacryloyl (GelMa), or (iii) fully synthetic ones, e.g., poly(ethylene glycols) (PEGs) and their derivatives.

In this section, we address the mechanical properties of the hydrogels most commonly used in microfluidics-assisted 3D cell culture. We discuss cell–hydrogel interactions and biodegradability of hydrogels as a general prerequisite for their applications in tissue engineering, and in particular their capability of mimicking the ECM of a given tissue.

### 3.1 Mechanical Properties of Hydrogels in Microtissue Engineering Applications

Mechanical properties of hydrogels forming 3D cell culture scaffolds not only determine the long-term stability of the scaffold but also directly impact the behavior of the embedded cells via mechano-transduction, i.e., biochemical signaling induced by external mechanical cues.^94^ For example, stem cells tend to retain higher levels of pluripotency when embedded in softer hydrogels, a feature recently exploited in generation of stem-cell laden core–shell capsules with soft-hydrogel cores.^95^

**Figure 4:**
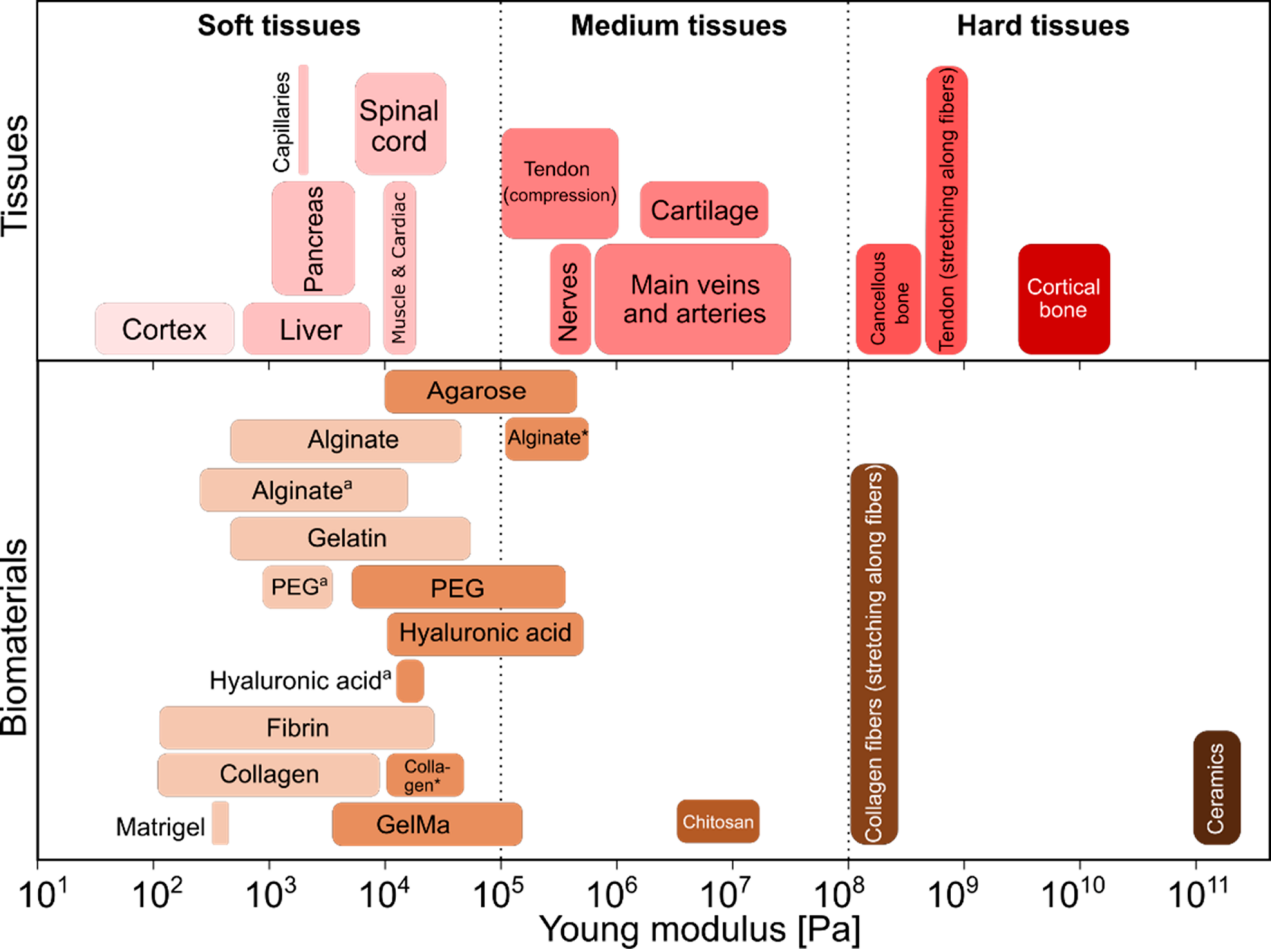
Tissues vs biomaterials: comparison of mechanical properties. Young’s moduli of various tissues (upper panel) and of the corresponding biomaterials most commonly applied in tissue engineering (lower panel). (*) Data for hydrogels cross-linked in nonphysiological conditions; (^a^) data for microgels.

Hydrogel mechanical properties are typically characterized by shear modulus (G) or elastic modulus (E) also called the Young’s modulus, which are both related to each other vis the material’s Poisson’s ratio (the latter typically in the range 0.45–0.5). Moduli of hydrogels can be tuned by changing various parameters, such as cross-linker type and concentration, as well as cross-linking time,^96^ which all impact the cross-link density defined as the number of cross-links per polymer chain.^97^ In particular, increasing the concentration of polymer leads to higher Young’s moduli of a cross-linked hydrogel.^98^ In biomimetic matrices, the mechanical properties of the matrix should match the properties of the native tissue or the native ECM, depending on the applied biomimetic strategy. A detailed comparison between the Young’s moduli of various tissues and various hydrogels is summarized in Figure 4. Tissues such as cortex,^99^ liver,^100,101^ pancreas,^102,103^ vasculature,^104^ muscle,^105^ and spinal cord^99^ with elastic moduli in the range 10^1^–10^4^ Pa can be classified as *soft* and therefore are usually cultured in soft hydrogels such as Matrigel, GelMa, gelatin, collagen, fibrin, dECM, or softer versions of alginate. Those hydrogels are also frequently applied in stem cell culture because the soft-solid microenvironment promotes spheroid formation and facilitates direct cell–cell interactions, which in turn leads to enhanced pluripotency.^95,106^ Interestingly, stem cells aggregating inside the soft core of a core–shell microcapsule^47,95,106−114^ tend to retain even higher levels of pluripotency as compared to spheroids cultured using conventional methods (nonadhesive substrates).^107^ Finally, soft hydrogels also provide optimal conditions for 3D culture of microtumors. Microencapsulation offers a unique tool for investigation of the impact of the mechanical properties of ECM on tumor progression. For example, Agarwal et al. used core–shell capsules with cancer cells contained in the soft collagen core^115^ to demonstrate that matrix stiffness alone (changed via doping collagen with alginate) impacts gene expression in breast cancer cells.

Tissues such as nerves,^116^ large vessels and arteries,^117^ as well as cartilage^118,119^ with moduli in the range 10^5^–10^7^ Pa can be classified as *medium* in terms of stiffness. Accordingly, biomimetic approaches involving these types of tissues require stiffer scaffolds which can be realized using hydrogels such as alginate, agarose, hyaluronic acid, PEG, or chitosan. Those biopolymers are frequently applied in bioprinting and general biofabrication of mesoscopic (milimeter- to centimeter-sized) tissue-like constructs^19^ but also used in generation of core–shell microstructures where they optimally serve as the shell phase. Mechanically stable alginate shells have been used in high-throughput microfluidic fabrication of stem cell spheroids^47,95,106−112^ and have also been shown to improve their cryopreservation.^120,121^

Tissues with the highest Young’s moduli, such as tendon and bone, can be classified as *hard* tissues. The moduli can reach even 10^10^ Pa in the case of cortical bone,^122^ up to 10^8^ Pa in case of cancellous bone,^123^ and up to 10^9^ Pa in case of tendon (upon stretching).^124^ It is worth mentioning that tendon is a strongly anisotropic tissue built of densely packed coaligned collagen fibers. It can be considered a hard tissue under stretching along the fiber direction,^125^ whereas upon compression or deformation in other directions, it behaves more as a medium-soft tissue.^126^ Even though the stiffness of manufactured materials such as those based on collagen fibers can be matched, e.g., with the stiffness of the tendon, such types of materials are not suitable as ECM mimics. Nevertheless, stiff biomaterials such as chitosan^66^ or ceramic scaffolds^127,128^ can be used to provide rigidity and stability to the engineered microtissues, e.g., necessary for their implantation in vivo.^128^ The most common strategy in regeneration of bone or cartilage tissue is the use of porous scaffolds. Rigid, porous structures warrant stability to the engineered constructs while also facilitating cell and nutrient infiltration into the scaffold.

Finally, we note that, in principle, the stiffness of a hydrogel sample may depend on the size of the sample. For example, the finite size may impact the cross-linking reaction, in particular, lead to a cross-link density gradient at the interface.^129^ Accordingly, in the case of microgels, it is desirable to measure the Young’s modulus directly. Several methods have been exploited for this purpose.^130^ For example, AFM-based nanoindentation was used to measure local mechanical properties of hydrogels at even nanometric scales, e.g., to detect local stiffness gradients at a hydrogel–hydrogel interface^131^ as well as to directly measure Young’s moduli of microgels.^132^ Another method, so-called real-time deformability cytometry, developed originally for cells,^133,134^ was used to extract the stiffness of microgels from the measurements of their deformation under viscous forces.^135,136^ Finally, elasticity of microgels have been also extracted from their static deformation under capillary forces emerging upon encapsulation of two (or more) microbeads inside an aqueous droplet.^137^ Importantly, the directly measured microgel stiffnesses typically remained of the same order of magnitude as those measured for bulk samples using conventional methods.^137^ From these latter results, one may conclude that, at least to the order of magnitude, the available “bulk” mechanical data can be used to approximate mechanical properties of the microgels.

### 3.2 Cell–Hydrogel Interactions in 3D Cell Culture

Cellular adhesion and proliferation are necessary to grow a healthy tissue. Some of the most important molecular factors that promote cell adhesion are the tripeptide sequence Arg-Gly-Asp (RGD) and fibronectin, whereas cell growth and proliferation are in general regulated by various types of growth factors. Growth factors can only be found in a small group of hydrogels of natural origin such as Matrigel or dECM, whereas cell adhesion motifs are naturally present also in chitosan, collagen, fibrin, gelatin, and GelMa but not in agarose, alginate, hyaluronic acid, or PEG. In the latter cases, cell adhesion can be promoted via proper chemical functionalization of the hydrogel.^109,138,139^

The required degree of cellular adhesion depends on the type of tissue. Tissues that tend to spread and form interconnected networks such as vasculature, in particular blood capillaries, typically rely on interaction with the surrounding ECM,^140^ which leads to formation of branched finger-like structures.^141−143^ Accordingly, hydrogels of choice in vascular tissue engineering include those which not only promote cell adhesion but are also soft enough to support cell migration. Indeed, the matrices frequently used in vascular tissue engineering include soft hydrogels such as fibrin^141,143^ as well as UV-cross-linkable PEG-fibrinogen,^144^ or RGD-functionalized PEGs.^142^ In 3D cultures aimed at generation of compact cell aggregates such as spheroids or organoids, cellular adhesion to the matrix should be minimized. In such types of applications, soft or even liquid-like microenvironments are advantageous.

### 3.3 Biodegradability of Microhydrogels in Vitro and in Vivo

Biodegradability of hydrogel scaffolds is one of the central issues in tissue engineering. In applications in which the hydrogel acts as a temporary support, the scaffold should gradually degrade as the tissue becomes mature. In such a cases, the degradation rate of the hydrogel needs to be matched with the rate of tissue development, which in turn depends on the type of tissue. Degradation of hydrogels is usually caused by one of two mechanisms: enzymolysis or hydrolysis. Enzymatic degradation is a local phenomenon, while hydrolysis occurs in the entire volume of the hydrogel due to the presence of unstable chemical bonds.^145^ Some hydrogels can undergo degradation in vivo without the need of further modification. Those include chitosan,^146^ collagen,^147^ dECM,^148^ fibrin,^149^ gelatin,^147^ GelMa,^84^ hyaluronic acid,^150^ and Matrigel.^151^ If their degradation rate is too fast, it can be slowed down by, e.g., introducing different cross-linking methods^152^ or adjusting cross-linking density.^153^ Several hydrogels such as agarose, alginate, and PEG do not undergo biodegradation in vivo. Various methods can be applied in order to enhance degradation such as modifications within polymer chains, e.g., oxidation of the alginate chain,^52^ incorporation of enzyme-sensitive molecules,^154^ or copolymerization with a biodegradable polymer.^155^

In in vivo applications, injectable bead-based scaffolds for wound healing,^156^ muscle,^46^ or neural regeneration^50^ should degrade possibly fast, with the degradation time of the order of weeks. In applications where implanted cells are supposed to act as a cure for prolonged periods of time, such as, e.g., in the case of insulin-producing beta cells^45,51^ or mesenchymal stem cells,^157^ the degradation time should be extended.^51^ This is typically achieved by the use of alginate whose mechanical and degradation properties can be additionally tuned, e.g., via adjusting the molecular weight.^132^

Finally, considering the degradation time of a hydrogel sample, one may expect the degradation time to actually depend on the size of the sample. In the case of the degradation due to enzymatic digestion, this time could be expected to be significantly shorter for granular hydrogels (or general microgels) as compared to the bulk nongranular samples due to the more efficient diffusion of the enzyme molecules into the hydrogel matrix in the former case, associated with the macroporosity. The data about biodegradability of microgels generated with microfluidics (>100 μm in diameter) are rather scarce, yet several reports are available in the literature.^158−160^ Considering the degradation of topological microgels, it is noteworthy that the compartmentalized structure can be used to degrade the different compartments selectively, a feature which could be further exploited in triggered release^158^ or to expedite cell aggregation within liquefied compartments.^161^

### 3.4 Most Common Hydrogels in Microtissue Engineering

**Figure 5:**
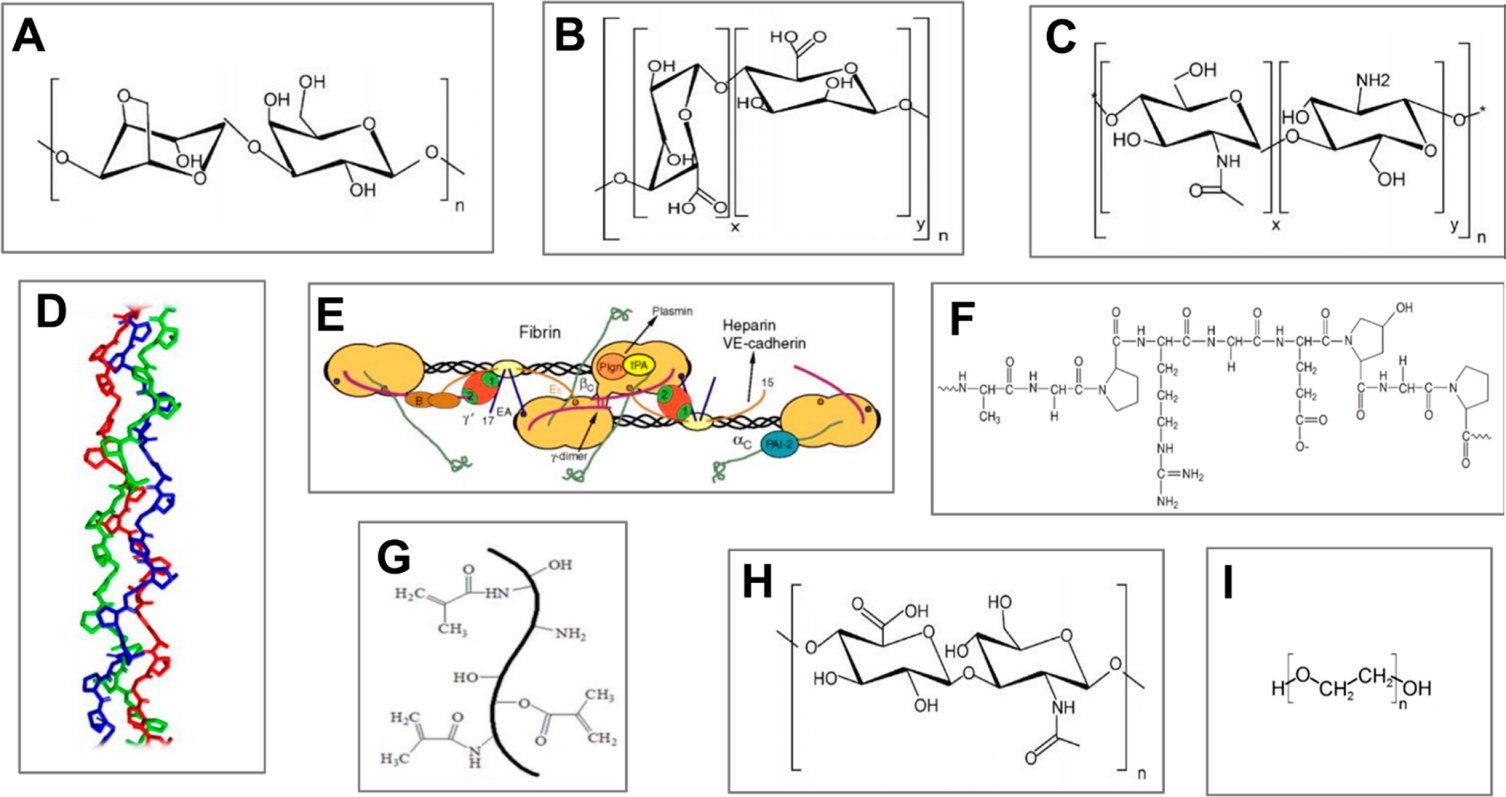
Chemical structure of biopolymers most commonly used in microtissue engineering. (A) **Agarose** consists of alternating d-galactose and 3,6-anhydro-α-l-galactopyranose units. Adapted with permission from ref (168). Copyright 2009 Wiley VCH. (B) **Alginate** consists of β-d-mannuronate (M) and α-l-guluronate (G) blocks. Adapted with permission from ref (168). Copyright 2009 Wiley VCH. (C) **Chitosan** consists of randomly distributed d-glucosamine and *N*-acetyl-d-glucosamine units. Adapted with permission from ref (168). Copyright 2009 Wiley VCH. (D) **Collagen** fibers self-assemble into a triple helix. Adapted with permission from ref (169). Copyright 2020 Wiley Periodicals LLC. (E) **Fibrin** is a protein that consists of binding sites for other proteins, enzymes, receptors, etc. Adapted with permission from ref (170). Copyright 2005 International Society on Thrombosis and Hemostasis. (F) Structure of **gelatin**. Adapted with permission from ref (171). Copyright 2005 Wiley VCH. (G) Schematic representation of **GelMa** structure. Adapted with permission from ref (172). Copyright 2019 The Royal Society of Chemistry. (H) **Hyaluronic acid** consists of alternating d-glucuronic acid and *N*-acetyl-d-glucosamine units. Adapted with permission from ref (168). Copyright 2009 Wiley VCH. (I) Chemical structure of unmodified **poly(ethylene glycol) (PEG)**. Adapted with permission from ref (173). Copyright 2012 The Authors.

In the following, we list the most common hydrogel-forming biopolymers applied in microfluidics-assisted microgel formulations. We explain their general cross-linking mechanisms and discuss properties from the point of view of microtissue engineering. The structural formulas of the biopolymers, if available, are listed in Figure 5. We address the different biopolymers in alphabetical order.

#### 3.4.1 Agarose

Agarose is a natural biopolymer derived from algae. It is a linear polysaccharide built of two main repeating units, d-galactose and 3,6-anhydro-l-galactopyranose (Figure 5A), and has the molecular weight of almost 12 kDa. Agarose chains form helical fibers that aggregate into coiled superstructures and **self-cross-link** via hydrogen bonding **upon cooling**.^162^ Young’s moduli of agarose gels may vary from several kPa up to several hundred kPa.^163,164^ Agarose lacks any cell adhesion sites or growth factors, however, it can be easily modified to provide such functional biomolecules.^165^ In microfluidics, agarose-based hydrogels have been successfully used to produce cross-linkable microdroplets,^72,166^ including core–shell structures.^167^

#### 3.4.2 Alginate

Alginate is a linear polyanionic block copolymer that is built from two main units: (1,4)-linked β-d-mannuroic (M block) and α-l-guluronic (G block) acids (Figure 5B). It can be derived from brown seaweed and is also produced by some microorganisms. The most extensively used gelation method for alginate is ionic interaction between polymer chains and divalent cations such as calcium Ca^2+^ or barium Ba^2+^. The cations form ionic bridges between polymer chains by attaching to anionic groups of alginate. It is assumed that cations preferably attach to G blocks of the alginate chains, which provide a high degree of coordination of the divalent ions.^174^ Alginate cross-linking has been widely exploited in microfluidics for generation of hydrogel microfibers, microbeads, as well as more complex compartmentalized microstructures.

Alginate itself does not contain RGD peptide motifs or other cell-adhesion cites so that the cells grown in pure alginate typically develop nonphysiological ball-like morphologies.^89,175^ The situation can be improved via using RGD-modified alginate.^76^ However, the relatively high rigidity of alginate suppresses cell spreading,^76^ which is in general disadvantageous in microtissue culture. Therefore, alginate is often mixed with other types of ECM-like hydrogels.^46,176^ To prevent microgel degradation, its surface can be additionally stabilized via coating with poly-l-lysine. Such modification allows long-term culture of alginate-ECM microgels.^176^

Considering mechanical properties, alginate is a hydrogel of intermediate stiffness, with Young’s moduli ranging from several to several hundred kPa^177,178^ depending on polymer concentration^179^ and alginate structure.^177^ Natively, alginates do not provide cell adhesion, but they can be modified with the adhesion motifs.^180^

Alginate polymers with molecular weight in the range 32–400 kDa^181^ find extensive applications in cell encapsulation. In particular, due to the almost immediate cross-linking of alginate upon contact with calcium ions, alginate precursors have been predominantly used as the shell phase in the core–shell capsules^75,95,107,108,111,115,175,182−191^ and core–shell fibers,^45,46,51,67,73,106,110,189,192−197^ various types of Janus and multicompartment Janus structures,^67,89,198^ including both capsules^75,76,176,199−201^ and fibers^67,79,82,199,202,203^ as well as all-cross-linked core–shell capsules,^184,185,204^ microdroplets,^205^ and microfibers^67,69,206^ with complex topology, droplet-loaded fibers,^60,68,207^ or helical fibers^208−210^ (see sections 4.4.4 and 4.5). 2D structures such as grooved microfibers,^211,212^ striped^83^ segmented^213^ hydrogel microsheets are also typically based on alginate (section 4.6). Finally, alginates also serve as the external cross-linkable phase in porous beads,^66^ porous hydrogel films,^71^ and porous 3D scaffolds^127,214,215^ (sections 4.4.3 and 4.7.4).

#### 3.4.3 Chitosan

Chitosan is a linear polysaccharide consisting of β-1,4-linked d-glucosamine and *N*-acetyl-d-glucosamine units (Figure 5C). It is derived from chitin, which is a natural polymer occurring in many crustacean species such as crabs and shrimp shells. Chitosan is extracted from chitin by acidic treatment followed by alkalization in order to remove proteins and some of the acetyl groups (partial deacetylation). Chitosan is normally not soluble in water, but in solutions with pH < 6.2, chitosan’s amine groups are protonated and chitosan becomes a soluble, positively charged polymer.^216^ It cross-links at pH above 6.2 or upon ionic interactions with negatively charged molecules.^217^ It is also possible to chemically cross-link chitosan, e.g., with genipin.^66,218^ Chitosan hydrogels have high stiffness, with Young’s modulus in the range of several up to several tens of MPa.^219^ They do not facilitate cell adhesion, but it is possible to provide chitosan hydrogels with, e.g., the RGD motifs.^220^

Due to the particularly high stiffness, chitosan has been used as a hydrogel additive or coating to improve mechanical properties of various topological microstructures such as porous materials,^66,218^ thin-shelled capsules,^217^ droplet-loaded fibers,^146^ or granular bioinks.^221^

#### 3.4.4 Collagen

The term “collagen” generally refers to a group of proteins, which are the most abundant structural proteins in the human body synthesized mainly by fibroblasts and osteoblasts. There are 28 types of collagen, which altogether constitute a third of the total protein content in the body and are the most prevailing components of ECM of many tissues, such as skin, bone, cartilage, teeth, or tendon. Collagens are built of three polypeptide chains that form a triple helix^222^ (Figure 5D). Despite the diversity of the collagen family,^223^ about 90% of the collagen present in human body belongs to the so-called fibrillar group, the most prominent example being collagen type I,^222^ which, due to its abundance, is extensively used in tissue engineering. Collagens can be cross-linked via self-aggregation caused by neutralization of collagen solution with, e.g., NaOH followed by heating up to the physiological temperature (37 °C).^224^ It is also possible to chemically cross-link collagen with noncytotoxic cross-linking molecules, e.g., genipin.^225^ Collagen hydrogels can be considered as soft biomaterials, with Young’s moduli of several hundred Pa^226^ up to several kPa,^227^ unless cross-linked in nonphysiological conditions such as higher pH and/or lower temperature^227^ (e.g., pH = 10, *T* = 4 °C), in which case the compressive moduli are in the range of 10–50 kPa. Collagens natively provide sustainable cellular adhesion due to the presence of the native cell adhesion motifs.^228^

Collagens find extensive use in microtissue engineering. For example, collagen type I has been used in preparation of microstructures such as cell-laden microbeads,^229,230^ core–shell^115^ and Janus microspheres,^176^ core–shell microfibers,^45^ as well as in bulk hydrogel matrices as an external hydrogel phase suspending other type of microgels.^231^

#### 3.4.5 Decellularized Extracellular Matrix (dECM)

dECM is a biomaterial produced by elimination of cells from the native ECM. It is a mixture of various macromolecular components found in native tissues, including cell adhesion proteins and growth factors, however, the actual composition is strongly dependent on the type of tissue from which ECM was derived. The process of decellularization is also highly specific to a given type of organ^232^ and involves removing of potential antigens, which could lead to inflammatory or immune response.^233^ Preparation of dECM-based scaffolds for tissue culture usually includes self-assembly of previously prepared solution in physiological conditions (37 °C).^234,235^ Mechanical properties of such scaffolds depend on biochemical composition and thereby on the type of tissue from which dECM is derived, but typically the Young’s moduli of dECMs are lower than those of native tissues.^236^

Because dECM is produced from tissues, it provides good cellular adhesion^237^ and has found applications in formation of tissue-specific cell-laden microbeads for organoid/microtissue engineering including heart,^238^ as well as liver, lung, kidney, muscle, intestine, or stomach microtissues.^239^

#### 3.4.6 Fibrin

Fibrin is a protein-based polymer that is a major component of blood clots and plays a key role in wound healing processes. Fibrin is formed by enzymatic polymerization of fibrinogen, a water-soluble glycoprotein with molecular weight of 340 kDa, built from two sets of intertwined polypeptide chains internally bridged by disulfide groups (Figure 5E). Cross-linking is mediated by thrombin,^240^ a serine protease present in blood. Fibrin is a biomaterial with low mechanical strength, exhibiting Young’s modulus in a range from several hundred Pa to several tens of kPa.^241,242^ Fibrin hydrogels natively provide cell adhesion sites.^243^

Fibrinogen has been extensively used as a precursor in preparation of microbeads for granular bioinks,^244^ as the inner phase in core–shell capsules^75^ and core–shell fibers^45,110^ or as the external matrix in 3D culture of vascular networks in the so-called angiogenic bead sprouting assays.^141^

#### 3.4.7 Gelatin

Gelatin is a derivative of collagen, produced by breaking of the collagen triple helices into single-stranded chains. Chemical composition of gelatin depends on collagen it was derived from, but generally it is built from amino acid sequence in which 1 of 3 subunits is glycine (Figure 5F). One can distinguish two types of gelatin, A and B, depending on the method of synthesis. Gelatin A is obtained by acidic treatment of collagen, while gelatin B is produced by its alkaline treatment. Both types have different isoelectric points (8.0 for type A and 4.9 for type B),^245^ which affects the overall net charge of the polymer chains in the solution. In tissue engineering applications, the most common gelatin type is type A.^65,246^ It can be cross-linked using various methods, whereas the simplest one is self-aggregation upon cooling.^246^ However, because native gelatin liquefies in physiological conditions, various derivatives of gelatin have been proposed to overcome this problem. For example, synthesis of gelatin containing phenolic hydroxyl groups^246^ or thiolated gelatin^247^ have been reported, cross-linkable via enzymatic reactions^65,246^ and via Michael-type addition,^247^ respectively. Such hydrogels are relatively soft with the Young’s moduli ranging from several hundred Pa to several tens of kPa.^248,249^

In general, gelatin natively promotes cellular adhesion,^250^ which makes it excellent biomaterial for fabrication of porous scaffolds^65,127^ as well as in preparation of all-hydrogel microstructures for cell encapsulation.^246^

#### 3.4.8 GelMa

GelMa is an acronym standing for gelatin methacryloyl, also called gelatin methacrylate, another derivative of gelatin. We dedicate a separate section to GelMa due to its widespread use in tissue engineering. GelMa is produced via chemical reaction between methacrylate groups of methacrylic anhydrite and the amine groups of gelatin^251^ (Figure 5G). It has mechanical properties resembling the ECM of soft tissues such as muscle, liver, or pancreas and can remain solid in physiological temperature (unlike native gelatin).^252^ Most popular method of GelMa cross-linking relies on the UV-induced photopolymerization in the presence of a photoinitiator.^67,197,253^ GelMa hydrogels are stiffer than unmodified gelatin, with Young’s moduli in the range from several kPa up to several hundred kPa.^254^ Similar to gelatin, GelMa supports cell adhesion.^251^

GelMa has been extensively used in preparation of microstructures, e.g., core–shell microdroplets,^253^ core–shell and Janus microfibers,^67,197,202,255^ microrods,^84^ as well as porous structures based on microfluidic foams^256^ and granular bioinks.^221^

#### 3.4.9 Hyaluronic Acid (HA)

Hyaluronic acid is a linear polysaccharide that is natively present in the ECM. It takes part in many biological processes, such as wound healing, cell signaling, and proliferation. It is built from a repeating disaccharide unit (glucuronate and *N*-acetyl glucosamine) (Figure 5H). HA can be derived from mammalian tissues (such as rooster combs), but it can also be produced via a microbial fermentation in *Escherichia coli*.^145^ Natively, it is usually present in macromolecular form (1–10 MDa), however, for hydrogel preparation usually low molecular versions are used and are achievable via acidic or basic treatment of macromolecular HA. HA and its derivatives (such as thiolated HA^247^ or methacrylated HA) can be cross-linked using UV light^257^ or Michael-type addition.^247^

Hyaluronic acid and its derivatives (e.g., its methacrylated version, HAMA) have been used in production of hydrogel core–shell microstructures, hybrid hydrogel scaffolds for cell culture,^247^ or hollow microfibers.^255^

#### 3.4.10 Matrigel

Matrigel is a trademark for a Corning company product, a complex mixture of various ECM components extracted from Englebreth–Holm–Swarm (EHS) tumors in mice. Its primary constituents are structural proteins, such as laminin, nidogen, and collagen, with total protein concentration of 8–12 mg/mL.^258^ Matrigel also contains heparan sulfate proteoglycans (which promote cell adhesion), growth factors like TGF-β and EGF, and small amounts of other proteins. However, exact composition of Matrigel can vary depending on the batch. After dilution of frozen Matrigel in PBS, the solution remains liquid at low temperatures and self-assembles into a hydrogel at the physiological temperature.^145^ Structural organization of Matrigel is caused by nidogen, which interacts with laminin and collagen as a bridging molecule.^259^ Even after cross-linking, Matrigel remains very soft, with Young’s modulus not exceeding 1 kPa.^260^

Matrigel has been used as the encapsulant in droplet-based organoid engineering,^20^ in particular as the material forming the core of the core–shell capsules^75,120,184,190,191^ or fibers,^193,261^ the inner coating of the core–shell microcapsules for neuron culture,^108^ or the core–shell fibers for blood vessel engineering,^262^ as well as in generation of hydrogel Janus microrods.^84^

#### 3.4.11 Poly(ethylene glycol) (PEG)

PEG is a hydrophilic synthetic polymer that is extensively used in biomedical applications. The basic PEG structure consists of (CH_2_–CH_2_–O) building blocks and has two hydroxyl end groups (Figure 5I). However, those end groups can be converted into other functional groups (i.e., methoxyl, carboxyl, amine).^263^ Considering spatial structure, PEG can form linear or branched polymers. Because PEG derivatives are frequently used in tissue engineering, the cross-linking mechanism depends on type of modification. However, most PEG derivatives can be cross-linked via Michael-type addition^264,265^ or UV light.^93,266^ PEGs are considered as biomaterials with intermediate mechanical strength, with Young’s modulus ranging from several kPa up to several hundred kPa.^267^ They do not promote cellular adhesion, but they can be easily provided with, e.g., RGD.^268^

PEGs have found extensive use in formulation of topological microgels, e.g., bulk macroporous hydrogels,^269^ jammed granular bioinks,^72^ porous hydrogel films,^71^ or core–shell structures.^265^

A summary of various properties of chosen hydrogels including their applications in generation of topological microtissues can be found in Table 1.

**Table 1:** Summary of Hydrogel Properties and Their Application in Generation of Topological Microtissues (TM)

hydrogel	gelation methods	Young’s modulus	cell–matrix interactions	biodegradability	cell types	application in TM
agarose	temperature (cooling)^72,166^	10–800 kPa^163,164^	possible after modification	possible after modification	mouse embryonic stem cells^167^	microdroplets for cell coculture,^72^ shell in core–shell droplets^270^ or Janus structures^166^
alginate	ionic^95,132,271^	5–50 kPa,^177^ 0.2–20 kPa^132,177^ (microgels), 150–540 kPa^178^ (nonphysiological conditions)	possible after modification	possible after modification	stem cells,^66,205,215,272,273^ HUVECs,^203,205^ PC12,^274^ rat neural Schwann cells (RSC 96),^114^ rat embryonic neurons,^82^ osteosarcoma cells,^255^ cardiomyocytes,^203^ human liver cancer cell line (HepG2)^194^	inner^185^ and outer phase^108^ in core–shell droplets, bead-loaded,^198^ core–shell^67^ and Janus^199^ microfibers, porous hydrogel films,^71^ porous scaffolds^214^
chitosan	pH, ionic,^196,275^ chemical^146,218^	6–20 MPa^219^	possible after modification	yes	PC12^50,221^	bead-loaded fibers,^221^ porous foams^218^
collagen	thermal^229^	0.1–10 kPa^226,227^ (physiological conditions), 10–50 kPa^227^ (nonphysiological conditions)	yes	yes	HUVECs,^45,115,208^ stem cells,^95,106,110,112,231^ PC12,^276^ preantral follicles,^52,95^ C2C12,^195^ cortical neurons^45^	coculture systems,^231^ core–shell microspheres,^230^ scaffold for cell culture^229^
decellularized ECM	thermal^238,239^	depends on tissue	yes	yes	stem cells,^110,192^ cardiomiocytes,^45^ HUVECs,^45,262^ smooth muscle cells,^262^ MCF-7 cancer cells,^115^ cortical neurons^45^	scaffold for tissue culture^238,239^
fibrin	enzymatic^144,244,275^	0.1–30 kPa^241,242^	yes	yes	cardiomiocytes,^45,277^ C2C12,^73^ HUVECs,^277^ fibroblasts^73^	microbead generation,^244^ inner phase in core–shell fibers,^110^ hybrid hydrogels for cell culture with PEG^144^
gelatin	thermal,^246^ enzymatic,^246,278^ Michael addition^247^	0.5–81 kPa^248,249^	yes	yes	rat H9c2 myoblasts^279^	core–shell structures,^280^ porous foam^127^
GelMa	photoinitiated,^253^ thermal^84^	3–185 kPa^254^	yes	yes	Schwann cells (RSC 96),^221^ PC12,^50,221^ stem cells,^192,272^ HUVECs,^203,281^ cardiac precursor cells,^282^ osteosarcoma cells,^255^ cardiomiocytes,^203^ fibroblasts^281^	core–shell microdroplets,^253^ core–shell and Janus microfibers,^67^ microrods,^84^ porous foams^256^
hyaluronic acid + derivatives	photoinitiated,^283^ Michael-type addition^247^	10–500 kPa^257^ 10–25 kPa^136^ (microgels)	possible after modification	yes	HUVECs^255^	scaffolds for cell culture,^247^ microfibers^255^
matrigel	thermal^20,84^	400–480 Pa^260^	yes	yes	stem cells^106,108^	core–shell microstructures,^108^ microrods,^84^ microscaffold material^20^
PEG and derivatives	photoinitiated,^93,266^ Michael-type addition^264,265^	5–500 kPa^267^ 1–3 kPa^131^ (microgels)	possible after modification	possible after modification	stem cells,^131,284^ C2C12,^285^ HUVECs^131^	beads in bulk hydrogel,^269^ jammed beads,^72^ porous hydrogel films,^71^ core–shell structures^265^

## 4 Microfluidic Strategies of Formulation of Compartmentalized Microgels

Microfluidic methods of formulation of microgel compartments can be roughly divided into those relying either on generation of hydrogel droplets or on generation of hydrogel jets, where the former serve as templates for “0D” compartments and the latter for “1D” compartments. Rapid generation of “0D” microgels typically requires the use of an external phase immiscible with the dispersed liquid-hydrogel phase, such as a hydrocarbon oil or a fluorinated oil phase, which leads to a nonvanishing interfacial tension between the aqueous and oil phases and facilitates formation of the droplets. On the other hand, transient liquid-hydrogel jets can be readily generated by also using miscible hydrogel and external phases and used to template “1D” hydrogel microfibers provided a sufficiently fast cross-linking strategy.

**Figure 6:**
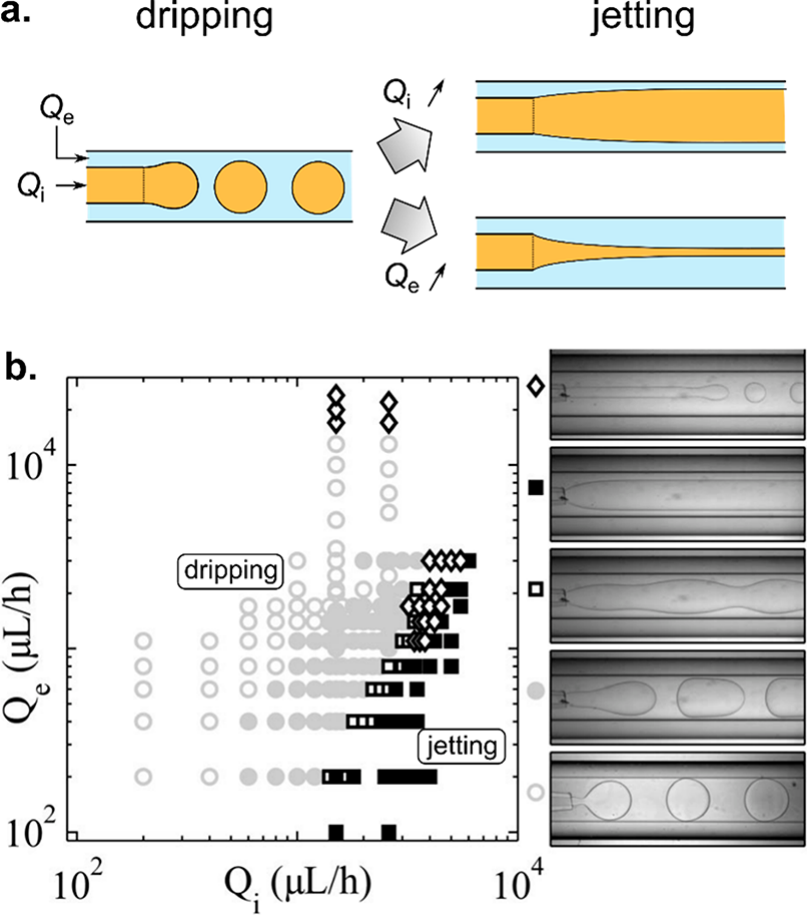
Dripping-jetting transition in a microfluidic coflow junction. (a) The transition can be achieved via increase of the rate of flow of inner phase *Q*_i_ or external phase *Q*_e_, resulting in either thick or thin jets. (b) Phase diagram spanned by (*Q*_i_, *Q*_e_) with indicated flow patterns observed in a coflow microfluidic device (concentric capillaries). Adapted with permission from ref (286). Copyright 2007 American Physical Society.

At this point, we make a note considering terminology. We use the term “droplet microfluidics” to refer to the systems which exploit at least two immiscible flows, while simply “microfluidics” to refer to the systems based on miscible flows (in the latter case the droplets typically do not form, only the jets). It is noteworthy that, even in the case of “droplet microfluidics”, the to-be-dispersed phase can actually also form jets (see Figure 6). We refer to such situations as the “jetting mode” of operation of a droplet–microfluidic device. In this section, we describe the conditions required for generation of droplets and jets in droplet microfluidics and provide examples of typical droplet–microfluidic junctions. The miscible flows are in general not suitable for generation of droplets but facilitate generation of jets. Jetting with miscible phases can be considered as a special case of droplet–microfluidic jetting with zero interfacial tension. Therefore, in the following, without losing generality, we consider only the case of immiscible flows.

In general, in droplet microfluidics, the to-be-dispersed liquid, phase A, and the immiscible external liquid, phase C (we reserve the notion of phase B for the second hydrogel phase which will be introduced later), are supplied via separate microchannels which then merge at a so-called microfluidic junction. Depending on the applied rates of flow, the two phases may flow in parallel, resulting in the formation of a jet of phase A in phase C, so-called *jetting mode*, or such that phase A breaks into droplets carried by phase C, so-called *dripping mode*(286−288) (see Figure 6a). The dripping mode is typically observed at low rates of flow of the external phase and low rates of flow of the dispersed phase, whereas sufficiently high rates of flow of either the dispersed phase or the external phase lead to jetting^288^ (Figure 6b). Due to the small lateral dimensions of the channels, typically of the order of 100 μm, the flows are laminar, which supports reproducibility of the flow patterns. In the jetting regime, this leads to stable jets of well-defined width while in the dripping regime to highly monodisperse droplets with coefficient of variation (CV) of droplet diameter, typically in the range 1–3%.^289^ The frequencies of droplet generation actually depend on the size of the generated droplets and range from the order 10^1^–10^2^ Hz for droplets of diameter roughly in the range 100–300 μm to 10^3^–10^4^ Hz for droplets of diameter in the range 10–50 μm.^290−292^ In applications involving encapsulation of cells for the purpose of formulation of microtissues, the number of encapsulated cells should be at least ∼10^2^ in order to allow rapid formation of microtissues via cell aggregation.^230^ Because typical cell concentrations in the hydrogel precursor are of the order of 10^7^ cells/mL, the droplet volume should be at least 10 nL, which then corresponds to typical droplet diameter of around 270 μm.

### 4.1 Generation of Droplet or Jets

In general, one can distinguish several different geometries of the microfluidic junctions which lead to different mechanisms of droplet breakup and determine the dripping/jetting regimes. The most common geometries, together with their advantages and disadvantages, are shortly listed below. The list includes droplet generators based on channels microfabricated in transparent chips, that is, plastic plates such as polycarbonate, poly(methyl methacrylate) (PMMA), or Teflon plates, as well as in polydimethylsiloxane (PDMS). In all those cases the channels have typically rectangular or square cross-sections. In contrast, in the devices based on capillaries or needles, the channels naturally have a circular cross-section.

**Figure 7:**
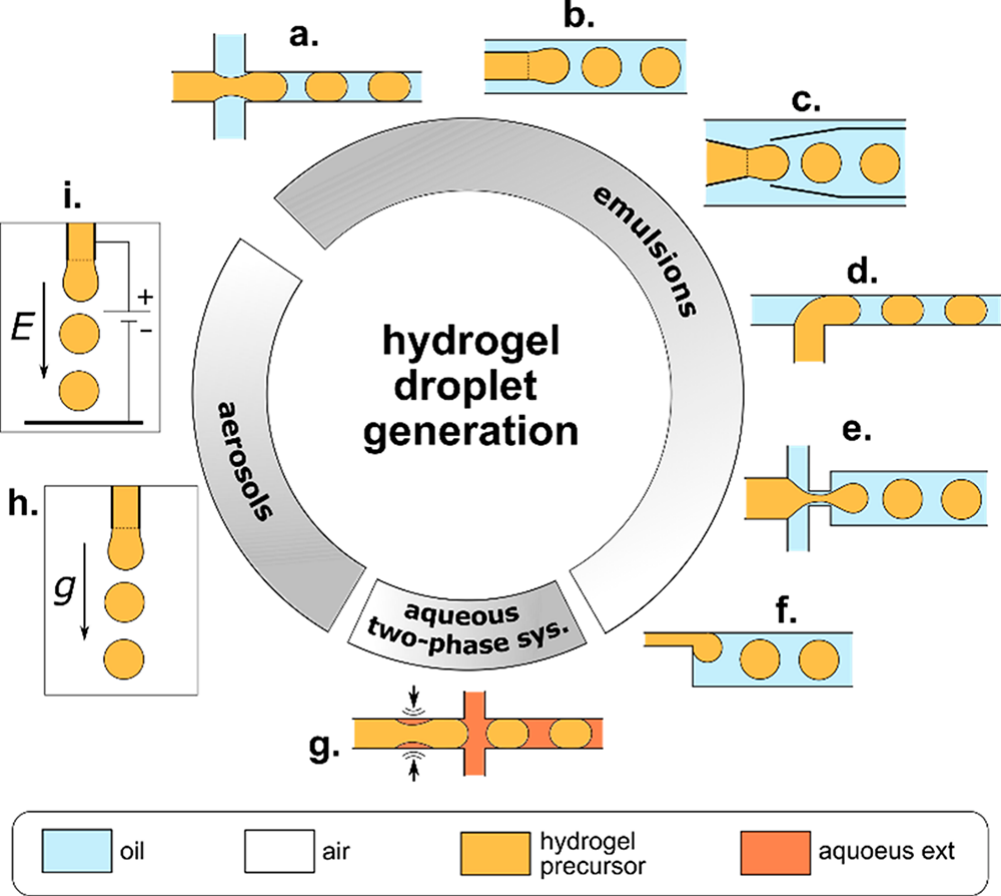
Microfluidic junctions used for generation of hydrogel droplets. We classify the geometries according to the type of generated dispersions: emulsions, aqueous two-phase systems, or aerosols. The list includes (a) cross-flow junction, (b) coflow junction, (c) concentric capillaries, (d) T-junction, (e) flow-focusing junction, (f) step junction, (g) pulse-based droplet generator, (h) gravity (or centrifugal) generator, (i) electric-field assisted generator.

- *Cross-flowjunction*. One of the simplest junctions consisting of two crossed channels,^287,288^ with the continuous phase supplied symmetrically from both sides of the dispersed phase (Figure 7a). The dispersed phase is periodically squeezed and pinched off by the continuous phase, resulting in formation of droplets.
- *Co-flow junction*. The geometry consists of two concentric tubes, needles (Figure 7b), or capillaries of circular cross-section, where the dispersed phase is delivered to the inner capillary (Figure 7c). This type of junction is easy to fabricate, as it does not require micromachining but only aligning of the capillaries. The droplets, generated by the Rayleigh–Plateau instability, are not squeezed by the walls in any direction, which eliminates the problem of wetting of the walls by the dispersed phase and facilitates droplet or jet cross-linking on-chip. The capillaries (or needles) can be nested one inside the other or assembled tip-to-tip, i.e., facing each other. In the latter case, the size of the droplets is set by the dimeter of the tip of the outlet capillary, which allows generation of very small droplets of diameters routinely below 100 μm.
- *T-junction*: In a T-junction, the channels meet at an angle 90° (Figure 7d). The main advantage of this type of junction is the simplicity of design and small footprint. The droplets are generated via shear-induced pinch-off.^291^
- *Flow-focusing junction*. The flow-focusing geometry is a modification of the cross-flow geometry (Figure 7e). The dispersed phase is focused into a narrowing by the continuous phase before breaking into droplets.^293^ The main advantages of the geometry are high achievable frequencies of droplet generation and small droplet sizes.^292^
- *Step junction*. The geometry consists of a shallow supply channel and a deep outlet channel separated by a step (Figure 7f). The droplets are generated via the imbalance of the LaPlace pressure upstream and downstream the step,^294^ and the droplet size is set predominantly by the depth of the supply channel. In particular, the droplet size is nearly independent of the applied rates of flow.^295^ Step junctions can be easily parallelized: devices with over 500,^296^ 1000,^297^ or even 10000^298^ parallel nozzles have been demonstrated. However, this type of junction is typically not suitable to formation of jets.
- *Pulse-based droplet generator*: In this type of droplet generator, the droplets are generated via periodic mechanical pulses exerted, e.g., via piezo-transducers^299^ or pressurized microchannels^197^ positioned next to the supply channel (g); geometry has little impact on droplet formation in this case, whereas the role of the external phase is just to carry away the generated droplets. Pulse-based generators have been applied in systems with extremely low interfacial tensions such as aqueous two-phase systems (e.g., consisting of PEG-rich and dextran-rich phases).
- *Body-force-base generators*. The geometry of droplet generators based on body forces, such as gravity, buoyancy, or centrifugal forces, consists of an outlet (a tip of a needle or capillary) of the dispersed phase coaligned with the direction the external body force (Figure 7h). The droplets are generated either in the jetting regime in which the ejected fluid breaks into droplets via Rayleigh–Plateau instability^300^ or in the dripping regime in which the fluid forms a growing droplet at the outlet which subsequently pinches off under the body force.^62,66^ The method is relatively simple as it does requires neither microfabrication nor aligning of capillaries, and as such it has been widely applied in the formulation of hydrogel microcapsules and microfibers.^62,66,300^
- *Electric-field assisted generator*. This type of generator resembles the above-mentioned body-force generators in that it involves an external field, in this case the electric field^66,75,301^ (Figure 7i). The droplets are pulled off an electrified needle and their sizes can be adjusted via tuning the applied voltage. The dynamic range of droplet sizes is significantly larger than in the case of a simple gravity-based generator.^66,301^ In particular, smaller droplets of sizes close to the needle-tip diameter can be readily generated, whereas gravity-based generators lead to droplets of diameter close to the capillary length, i.e., typically around 1 mm, only weakly depending on the diameter of the needle.^58^

Finally, one can also distinguish *reconfigurable junctions* whose geometry, e.g., the size of the orifice in a flow-focusing geometry, can be altered on-demand. For example, in a PDMS flow-focusing junction, the orifice can be squeezed by the pressurized air pockets placed on both sides of the narrowing and used to control the droplet/bubble size on-demand without changing the rates of flow;^289^ this type of junction has been recently employed, e.g., in 3D printing of functionally graded porous materials.^54^

In the following, we review the microfluidic strategies of formulation of microgels, which in general must take into account the type of applied hydrogel and its cross-linking mechanism. In many cases, the physicochemical factors involved in the cross-linking process are the ones that determine the layout and/or dimensions of microchannels and microfluidic junctions.

### 4.2 Physical Cross-Linking of Hydrogel Droplets and Jets

Considering general cross-linking mechanisms applied in microfluidics-assisted generation of microgels, one can distinguish chemical and physical cross-linking. Physical cross-linking relies on self-assembly of hydrogel molecules into a network induced by a change in solution temperature or mediated by physical (noncovalent) interactions between polymer chains and a cross-linker, such as ionic interaction, hydrogen bonding, or host–guest complexation.^302^ Physically, cross-linked hydrogels, due to the relatively weak nature of the molecular “physical” interactions, i.e., as compared to the covalent bonds in chemically cross-linked hydrogels (see section 4.3), are usually soft and easily degradable. The advantage of the physical cross-linking process are mild conditions which allow the embedded cells to retain high levels of viability.

#### 4.2.1 Ionic Cross-Linking

**Figure 8:**
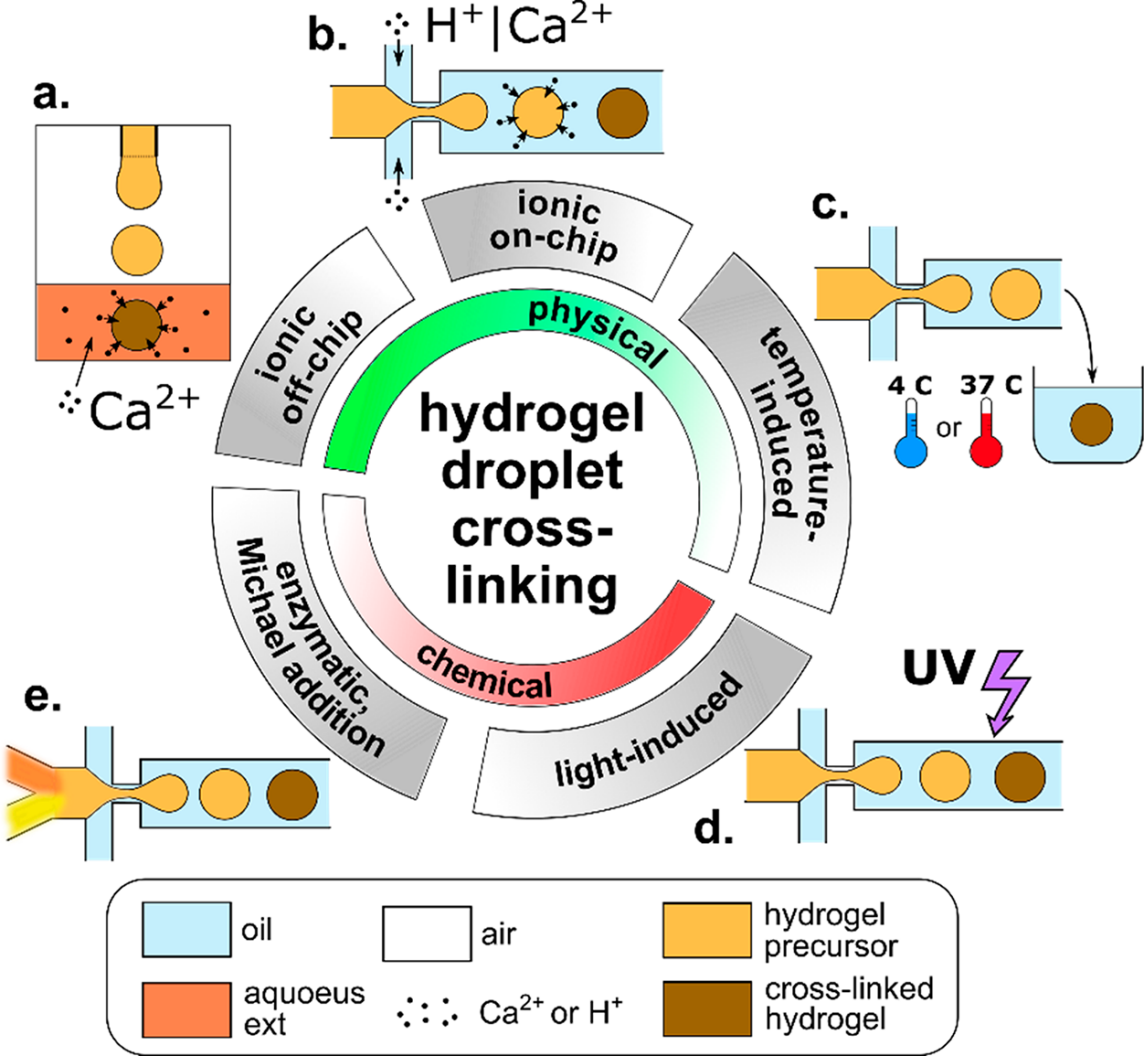
Microfluidic strategies of cross-linking of hydrogel droplets. (a) Ionic off-chip cross-linking, (b) ionic on-chip cross-linking, (c) temperature-induced, (d) light-induced, (e) chemical reaction-based (such as Michael addition or enzymatic cross-linking). In ionic on-chip cross-linking (b), the calcium ions are either delivered directly in the external phase (“Ca^2+^”) or trigger-released from the hydrogel precursor upon contact with the acidic external phase (“H^+^”).

One of the cross-linking methods particularly widespread in microfluidics is the so-called ionic cross-linking. Typically, the method involves the use of sodium alginate which cross-links in the presence of calcium cations Ca^2+^ into gelous calcium alginate. Upon coalescence of an alginate droplet with an external calcium bath, the cross-linking proceeds via rapid quench of the droplet interface, which takes of the order of milliseconds or shorter as can be judged from the fastest available microgel formulation frequencies (∼10^5^ Hz^62,199,303^). The cross-linking time scale is accordingly typically much shorter than the time scale associated with mixing of the nanoliter liquid compartments and allows generation of the compartmentalized architectures. One can distinguish two methods of alginate cross-linking applicable to droplets and jets: (i) off-chip cross-linking (Figure 8a) achievable via coalescence with an external aqueous bath containing calcium ions and (ii) on-chip cross-linking (Figure 8b). In the latter case, calcium ions can be contained in the external oil phase (“Ca^2+^” method) or released from the droplet phase upon reaction with an organic acid dissolved in the external phase (‘H^+^’ method).

In the “Ca^2+^” method, the calcium ions can be dispersed in oil in the form of nanoemulsion^95,107,304^ or directly dissociated. In the latter case, an oil-soluble calcium source must be used, such as calcium acetate.^305^ Otherwise, dissolution of calcium chloride in oil can be mediated with the use of an alcohol such as 2-methyl-1-propanol.^306^ To provide complete gelation without channel clogging, the total time required for reaction must be shorter than the time within which droplets are present on the chip but longer than the droplet formation time, which in general puts a constraint on the available rates of flow. An alternative clogging-free modification of the approach is the formulation of a W/O/W emulsion containing alginate as the inner aqueous phase, calcium nanoemulsion as the middle oil phase, and water as the outer phase.^307^

In the “H^+^” method, calcium ions are released from a calcium compound such as Ca-EDTA or CaCO_3_ pre-encapsulated in the droplet phase, e.g., upon a pH change at contact with the external oil phase containing dissolved organic acid, e.g., acetic acid.^184,271,308−310^ The acid dissociates at the droplet surface releasing H^+^ cations which react with the calcium compound, triggering the release of Ca^2+^, which in turn gradually cross-links alginate. This relatively slow gelation method requires incubation of droplets to complete the cross-linking reaction. The method is easy to perform and results in a homogeneous gelation in the whole droplet volume, but pH drop caused by H^+^ release may have a negative effect on cell viability.^311^

It is noteworthy that sodium alginate is not the only prepolymer that can be cross-linked with ionic interactions. Chitosan, which contains amine groups, becomes positively charged in solutions with pH < 7. Therefore, it can be cross-linked using multianionic cross-linkers, such as P_3_O_10_^5–^.^312^ Another approach, involving the use of chitosan and aimed specifically at generation of core–shell structures, has been recently proposed by the Qin group.^217,275,313^ The method relies on the use of a two-phase aqueous system with alginate-rich droplet phase and chitosan-rich external phase, in which alginate–chitosan complexation results in formation of capsules^217,275^ or fibers^304^ with ultrathin shells.

Lastly, we note that microfluidic cross-linking of alginate microfibers has been also demonstrated using barium cations Ba^2+^.^74,206^

#### 4.2.2 Temperature-Triggered Cross-Linking

Temperature-triggered cross-linking is often used due to the ease of application and a variety of hydrogels that cross-link upon cooling or heating. One can distinguish hydrogels with an upper critical solution temperature, *T*_crit,up_, which cross-links below *T*_crit,up_ and those with a lower critical solution temperature, *T*_crit,low_, which cross-links above *T*_crit,low_. In the hydrogels commonly applied in tissue engineering, the cross-linking relies either on the formation of hydrogen bonds between polymer chains upon cooling, typically down to 4 °C, such as in the case of gelatin,^246^ GelMa,^84^ or agarose^72,166^ or on interactions between proteins, such as, e.g., in collagen,^229^ Matrigel,^20,84^ or decellularized ECM,^238,239^ achievable via elevation of temperature up to physiological 37 °C. Temperature-triggered gelation in most cases happens off-chip (Figure 8c), but it can also be obtained on-chip by cooling the whole microsystem with hydrogel microdroplets inside.^314^ Cross-linking of the core phase in the core–shell structures can be conveniently achieved via change in temperature following, e.g., ionic shell cross-linking.^20,229,246^ Also, ionically cross-linked Janus microgels consisting of a mixture of alginate and collagen (or Matrigel) have been additionally temperature-cross-linked, which allowed subsequent alginate dissolution for generation of soft Janus capsules.^84,176^

#### 4.2.3 Host–Guest Interactions

Another method of physical microgel cross-linking involves host–guest interactions widely investigated in the field of supramolecular chemistry in recent years. Host–guest interactions rely on recognition of molecular motifs and formation of noncovalent bonds. As such, and because of their dynamic nature, host–guest interactions resemble the molecular interactions in biological systems, including those responsible for cross-linking of biopolymers. In synthetic hydrogels, cyclic compounds such as cyclodextrins can be used as “hosts” in formulation of cross-links by providing reversible bonds with various “guest” units.^315^ Such strategy offers a great potential in biomaterial design, and recent works indicate also a possibility of its adaptation in microfluidic formulation, e.g., of core–shell capsules.^316,317^ However, to date, there are no available reports of the application of such structures in encapsulation of mammalian cells.

In summary, various physical cross-linking methods can be used to formulate microgels. In particular, ionic cross-linking proves advantageous in processes requiring rapid quenching of miscible hydrogel compartments, such as in solidification of the shell-forming phase in the precursor core–shell structures. On the other hand, mild conditions involved in the temperature-triggered cross-linking or host–guest interactions are advantageous in terms of cell viability and often yield hydrogels better mimicking the actual ECM. Noteworthy, various cross-linking approaches can also be combined.^108^

### 4.3 Chemical Cross-Linking of Hydrogel Droplets and Jets

Chemical cross-linking is mediated by chemical reactions in which a polymer chain forms a covalent bond with a cross-linker molecule. Examples include light-induced cross-linking, enzymatic cross-linking, and Michael-type addition. Because residual cross-linking molecules such as free radicals can interact with the biological content of the sample (proteins, cells),^302^ the choice of the cross-linker is crucial for biocompatibility of the hydrogel. Despite potential cytotoxicity, chemically cross-linked hydrogels typically develop better mechanical properties (e.g., higher Young’s and storage moduli) and lower degradation rates as compared to physically cross-linked ones.

#### 4.3.1 Light-Induced Cross-Linking

In light-induced cross-linking, a polymer solution is mixed with a photoinitiator and exposed either to UV or visible light, which leads to homogeneous breakage of bonds in the photoinitiator molecules, resulting in the release of free radicals. As free radicals are very reactive, they form bonds between the polymer chains, which in turn lead to fast hydrogel cross-linking. Short gelation time provides high control over the cross-linking process; in particular, it can be used to “quench” (Figure 8d) nonspherical droplet shapes^318^ or prevent mixing of different hydrogel compartments. It allows continuous cross-linking of droplets on-the-fly either in the outlet tubing^282,319^ or on-chip.^93,266,320^ The droplets may also be cross-linked after their collection in an external chamber, however, in such a case their shape may be affected by contact with other droplets.^321^ In some cases, on- and off-chip cross-linking may be combined. This strategy has been used, e.g., in order to anneal the prepolymerized droplets into a porous hydrogel scaffold^322^ (see also section 4.7.1). The most common UV-cross-linkable hydrogel used in generation of cell-laden microgels is gelatin methacryloyl (GelMa), which well mimics the extracellular matrix.^253^ The time scale of cross-linking of GelMa depends on light intensity and the concentration of the photoinitiator in the hydrogel precursor and can reach down to several seconds at high applied UV intensities.^253^ Such a time scale is typically short enough to allow on-chip cross-linking of droplets, which is advantageous in terms of microgel uniformity and reproducibility. It has been applied mostly to generate simple microbeads and bead-based porous scaffolds,^160^ as well as core–shell capsules with GelMa shell.^253^ In general, however, in one-step fabrication of complex microgel architectures, the time scale of UV-induced cross-linking, as compared to, e.g., ionic cross-linking, may be considered a limiting factor.

Besides GelMa, also other hydrogels such as hyaluronic acid derivatives,^72,247,323^ PEG-fibrinogen,^46^ PEG derivatives,^93,266^ or gelatin-PEG derivatives^324^ have been applied in UV-mediated cell encapsulation. Topological core–shell or Janus microstructures with single or multiple solid or liquid cores have been demonstrated using poly(ethylene glycol) diacrylate (PEGDA),^325,326^ ETPTA^89^ or polyacrylamides.^63,90,327^ However, the utility of acrylates and acrylamides in cell encapsulation and culture can be questioned. In particular, non-fully cross-linked acrylamides as well as certain acrylic resins such as ETPTA are known to be cytotoxic.

#### 4.3.2 Enzymatic and Michael Addition-Based Cross-Linking

Some hydrogels can be cross-linked enzymatically or via Michael type addition reactions. In such cases, the reactions are initiated after mixing of the biopolymer with an enzyme or cross-linker, respectively. Enzymatic cross-linking is in general favorable for cells because cross-linkers are typically not cytotoxic. Examples include cross-linking of gelatin with microbial transglutaminase,^278^ phenol-modified gelatin with horseradish peroxidase,^246^ and hydrogen peroxide^328^ or fibrin with thrombin.^244^ Typically, separate streams containing the polymer and the cross-linker (or enzyme) molecules merge on-chip prior to droplet generation, followed by intradroplet mixing (Figure 8e) and gradual gelation on-chip or off-chip. The process of cross-linking typically takes from several minutes to hours, depending on the applied reactant concentrations. In topological microstructures enzymatically cross-linkable hydrogels such as fibrin are typically used as “dopants”, e.g., to provide ECM-like microenvironment, whereas mechanical stability is provided by another hydrogel such as alginate.^75,110^

Hydrogels prepared with PEG-derivatives may be also cross-linked using Michael-type addition reactions which involve formation of a chemical bond between a modified PEG chain (e.g., vinyl sulfone groups) and a cross-linker, e.g., a thiol group containing molecule.^329^ Similar to the case of enzymatic cross-linking, the reactants are mixed on-chip prior to droplet generation. For example, reaction between thiol-modified poly(ethylene glycol) (PEGSH), PEGDA, and heparin was used to generate simple hydrogel microspheres for encapsulation of stem cells,^264^ whereas PEG modified with four maleimide groups (PEG4MAL) and dithiothreitol (DTT) were used to fabricate core–shell structures with a liquid core for hepatocyte culture.^265^ It should be mentioned that hydrogels that do not contain PEG can also be prepared via Michael-type reactions. For example, modified poly(vinyl alcohol) (PVA)^330^ or thiol-modified gelatin (Gel-SH) and vinyl sulfonated hyaluronic acid (HA-VS) hybrid hydrogels^247^ were also used to encapsulate cells. Finally, PEG-based thiol- and acrylate-functionalized biopolymers have been also used as the substrates for intradroplet microfluidic cross-linking.^49^

#### 4.3.3 Click Chemistry-Based Cross-Linking

Another chemical cross-linking method that can be used in fabrication of microgels is based on the so-called click chemistry. This type of cross-linking relies on the use of heteroatoms, examples including copper-catalyzed azide–alkyne cycloaddition, Diels–Alder reaction, or thiol–ene photocoupling.^331^ Click chemistry reactions are designed to function in complex biological environment and as such to meet the requirements of modularity, width in scope, high yields, inoffensive byproducts that can be easily removed, stereospecificity, and mild reaction conditions.^332^ Although click chemistry has been used in hydrogel cross-linking,^333^ microfluidic microgel formulation^136^ and even cell encapsulation,^159^ to date (with some exceptions^334^) there has been little research on its use in tissue engineering.

In summary, various chemical cross-linking methods have been successfully applied to formulate microgels. Among the available methods, the UV-triggered cross-linking has the advantage of short cross-linking times and therefore can be readily used to generate compartmentalized microgels even using miscible hydrogel precursors. On the other hand, the enzymatic, Michael addition, or click chemistry approaches are in general more biocompatible and technically easier to implement as they do not require integration of any external equipment (such as UV light source) into the microfluidic workflow, and as such provide optimal solution for less complex microstructures.

### 4.4 Generation of Compartmentalized “0D” Structures

Formation of “topological” structures with internal compartments necessarily requires the use of at least *N* = 2 different hydrogel phases plus the external carrier phase. One can distinguish different formulation strategies depending on the miscibility of the three phases, which also determines the type of microfluidic junctions and overall design of the microfluidic workflow. Below, we discuss several strategies of formulation of the complex liquid architectures in view of their applications as templates in synthesis of compartmentalized cell-laden microgels.

#### 4.4.1 Templating Based on the Equilibrium Liquid Architectures

**Figure 9:**
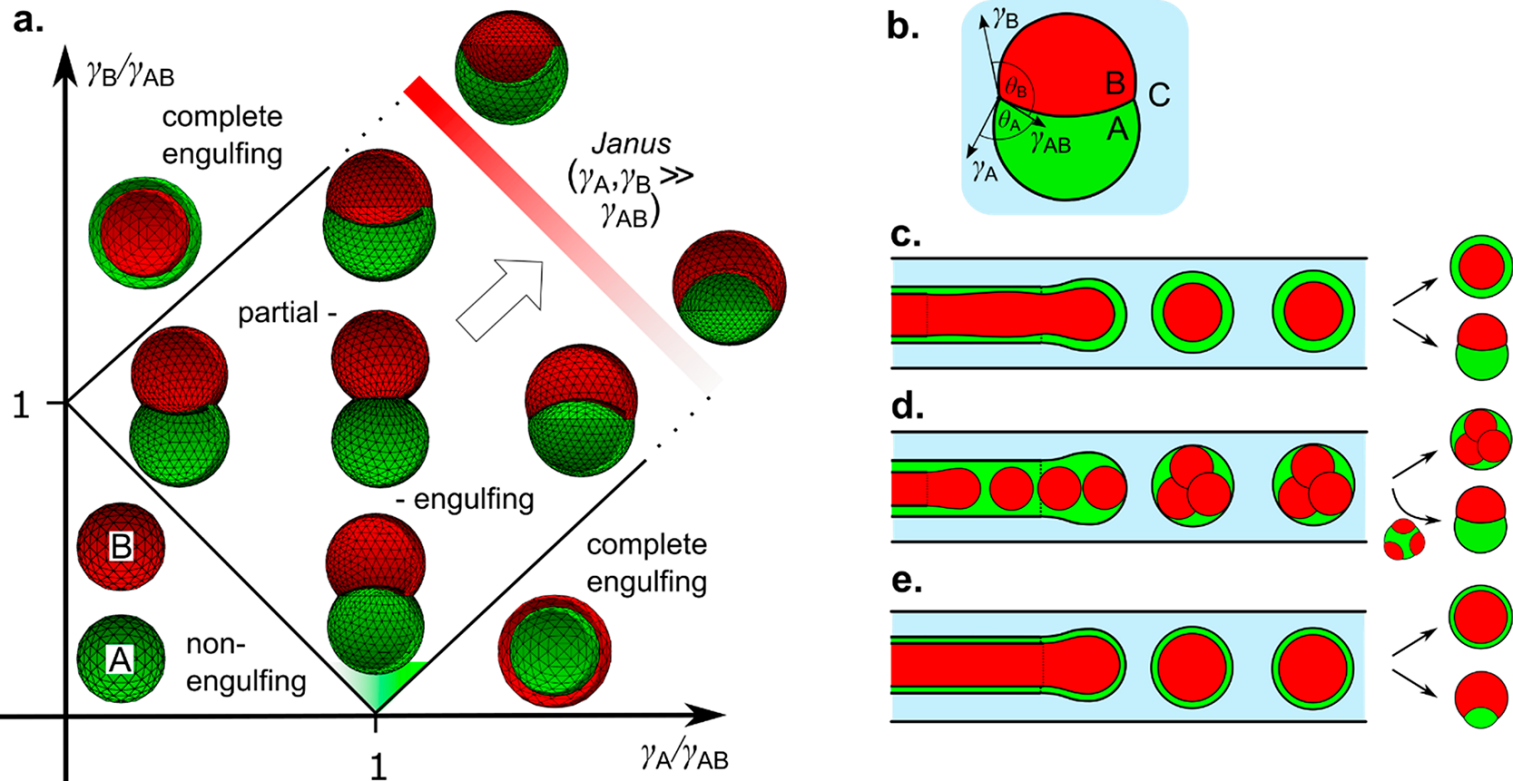
Equilibrium double-emulsion droplet morphologies. (a) Diagram spanned by the ratios of interfacial tensions γ_A_/γ_AB_ and γ_B_/γ_AB_. Adapted with permission from ref (58). Copyright 2012 Royal Chemical Society. (b) Schematic representation of the balance of forces acting at the three-phase contact line in a double-emulsion droplet. (c–e) Formulation of double emulsions based on two nested droplet generators. Generation of core–shell structures with (c) single core, (d) multiple cores, and (e) single core with ultrathin shell. The inner and outer droplet generators may operate either independently (d) or such that the breakup of the outer interface triggers also the breakup of the inner interface (c,e). The final equilibrium morphologies evolve according to the interfacial tensions. The structure with multiple B-cores in the “engulfing” regime remains stable provided that phase A contains a surfactant stabilizing the cores against coalescence. In the “partial engulfing” regime, the structure evolves into a transient state with multiple B-phase “buds”, which further coalesce into a single B-phase compartment. In general, in the case of partial engulfing, the sign of curvature of the A–B interface (concave vs convex) may change depending on the volume fraction (c vs e).

As already mentioned, in the case with *N* = 2 hydrogel phases, there are two available topologies: the core–shell and the Janus topology. In the case with immiscible precursors, the final topology emerges spontaneously in the process of equilibration of the liquid–liquid interfaces, which tend to minimize the interfacial energy.^57,58^ In such a case, the final topology depends solely on the values of the interfacial tensions between the three immiscible phases (Figure 9a) and not, in particular, on the applied volumes of the compartments, strategy of droplet formation, or the kinetics of the equilibration process. One should note that the notion of equilibrium in this case means that the equilibrium topologies are those which spontaneously emerge after a sufficiently long time. Typically, in systems without surfactants, this equilibration happens even within tens or hundreds of milliseconds, whereas in systems with surfactants, it can take the order of several seconds^57^ up to minutes or hours.^166^ In fact, surfactant-rich systems in many cases remain always out-of-equilibrium due to the Marangoni microflows, yet the topologies of the compound droplets evolve typically slowly enough to be used as templates for fabrication of compartmentalized microgels.

We now turn to the classification of the different topologies depending on the values of interfacial tensions between the different phases. In a system composed of droplet phases A and B suspended in an external phase C, the topologies are set by the relative values of the interfacial tensions γ_AB_, γ_AC_, and γ_BC_ (see Figure 9b, where we denote γ_AC_ ≡ γ_A_ and γ_BC_ ≡ γ_B_), the conditions γ_BC_ > γ_AC_ + γ_AB_ or γ_AC_ > γ_BC_ + γ_AB_ result in engulfing of phase B by phase A or vice versa, respectively. The condition γ_BC_ + γ_AC_ < γ_AB_ stabilizes a nonmerged configuration with phases A and B separated by phase C. The remaining region of the interfacial tension “phase-space” (Figure 9a) corresponds to the Janus configurations. One can actually use a more general notion of “partial engulfing”, in which the overall morphology of the double droplet is not strictly spherical but rather consists of two distinct semispherical buds. Then, the actual Janus topology emerges as a limiting case in which the interfacial tension γ_AB_ between the dispersed phases vanishes as compared to γ_AC_ and γ_BC_.^58^ In fact, in the case of miscible hydrogel phases γ_AB_ does tend to zero, which accordingly facilitates formation of Janus topology in this case. Yet, the lack of proper A–B interface typically results in mixing of the compartments, and the structure must be rapidly solidified before full equilibration of the interfaces in order to keep the compartmentalized architecture (section 4.4.4).

In microfluidics, double-emulsion droplets are typically formed using two nested junctions (or nozzles) in which the first junction introduces phase A into phase B and the second junction phase B into phase C.^31^ The generated liquid architecture (which forms the initial topology from which the final equilibrium topology evolves) depends on the mode of operation of the first junction. If the first junction operates in the jetting regime (Figure 9c), the jet gets dispersed into droplets at the second junction, resulting in core–shell double-emulsion drops with a single, large core.^335^ Alternatively, if the first junction operates in the dripping regime (Figure 9d), the device generates core–shell double-emulsion drops with single or multiple cores.^57,66,336−338^ In yet another approach, both droplet phases A and B are delivered directly to a single junction such that a compound A–B–C interface is formed (Figure 9e) and its breakup results in the formation of double-emulsion drops with a single core and, typically, a very thin shell.^339−341^ The generated core–shell topologies subsequently relax into the final equilibrium topology, which may be either the same core–shell (engulfing) topology or the partial-engulfing topology.^57^ If the interfacial tension between the droplet phases is particularly large, one can also observe escape of the inner core from the shell and formation of separate simple droplets of phase A and B suspended in C.^57^ Such situation is disadvantageous and must be avoided on the way toward generation of compartmentalized hydrogel droplets.

#### 4.4.2 Compartmentalized All-Hydrogel Microparticles

Despite the appeal of the equilibrium morphologies, they are difficult to achieve in the case of different hydrogel precursors which are typically miscible. This poses a challenge in direct application of the double-emulsion droplets as templates in generation of compartmentalized microgels. However, several approaches have been demonstrated that circumvent the problem of miscibility.

**Figure 10:**
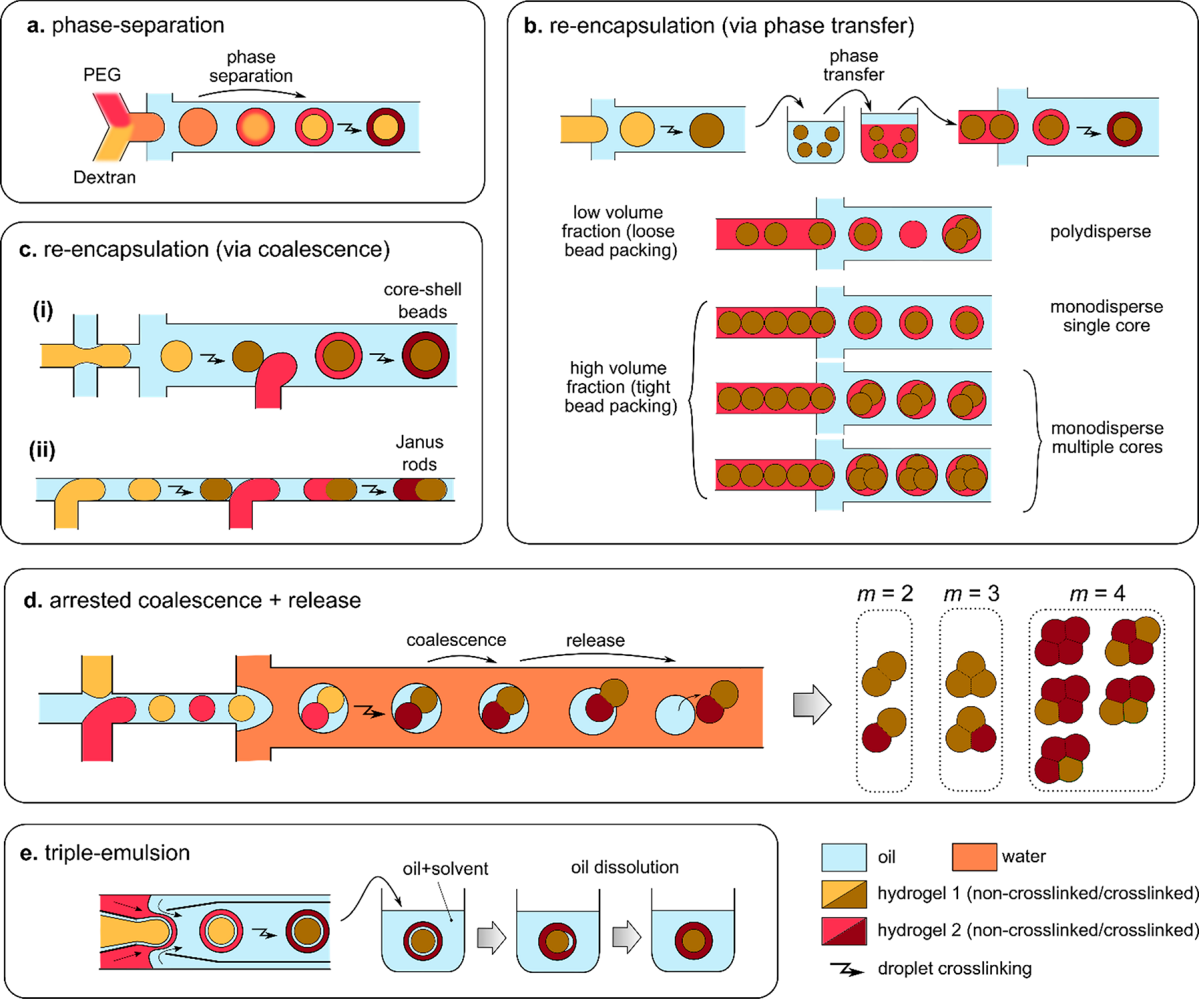
Microfluidic formulation of compartmentalized microgels based on the equilibrium double-emulsion-like topologies. (a). Phase separation into dextran-rich and PEG-rich phases.^342^ (b) Re-encapsulation assisted by off-chip phase transfer.^246^ Low volume fraction of beads in the hydrogel after phase transfer results in polydisperse droplets with a random number of encapsulated cores. High volume fractions result in bead ordering and monodisperse droplets with a narrow distribution of the number of cores.^63,343^ (c) Re-encapsulation via on-chip coalescence of a cross-linked droplet and a non-cross-linked droplet. (i) The unconfined droplets lead to core–shell spherical beads,^204,310^ whereas (ii) droplets confined in a narrow tubing result in Janus rods.^84^ (d) Arrested coalescence of pre-cross-linked hydrogel droplets encapsulated inside a drop of oil.^344^ After coalescence, the structure is spontaneously released from the oil via dewetting. (e). Triple-emulsion approach^345^ consisting of generation of W/O/W/O triple-emulsion followed by hydrogel phase cross-linking and dissolution of the middle oil phase in the external oil phase with an added solvent, such as, e.g., 2-propanol.^345^

The first of those approaches relies on the use of the aqueous two-phase (ATP) systems in which the hydrogel phases A and B phase-separate, forming an interface of ultralow interfacial tension. The separation is typically achieved via addition of phase-separating biopolymers, such as dextran and PEG, to the hydrogel precursor solution. After injection on-chip, the dextran- and PEG-rich phases mix at a Y-junction, and subsequently the mixture gets dispersed into droplets (Figure 10a). With time, the solution inside the droplets spontaneously phase-separates and develops distinct dextran- and PEG-rich compartments. The topology of such compound droplets, suspended in the external oil, such as fluorocarbon or organic oil, can be adjusted between the engulfing or Janus topology via tuning the concentrations of PEG and dextran^166^ as well as via changing pH.^342^ Subsequently, one or both of the compartments can be cross-linked (the case with two compartments has not yet been demonstrated in the literature but actually seems feasible). Accordingly, in the case with one cross-linked compartment, depending on the topology, the second non-cross-linked compartment can be either washed out, resulting in moon-shaped particles^166^ (for the Janus topology) or retained as a liquid core of a core–shell structure (for the engulfing topology).^342^

The second approach to compartmentalization of all-hydrogel compartments relies on re-encapsulation (Figure 10b,c), that is, reinjection of a preformulated microgel-in-hydrogel precursor suspension, that is a suspension of hydrogel beads, into a microfluidic junction and its redispersion into droplets, resulting in encapsulation of the beads inside larger liquid-hydrogel droplets. Because the interfacial tension between the cross-linked hydrogel (the bead) and the non-cross-linked hydrogel (the droplet) is close to zero (both phases are aqueous), the equilibrium topology is the “complete engulfing”. Another cross-linking step results in the final core–shell topology, with one or multiple solid-hydrogel cores inside a solid-hydrogel shell.^204,246^ One can further distinguish two different approaches to the re-encapsulation step: (i) phase transfer, in which the hydrogel beads are resuspended in the second hydrogel precursor (typically off-chip) and reinjected into another chip, and (ii) coalescence, in which the beads and the precursor droplets are generated at the same chip in unison, and such that they coalesce in pairs. Noteworthy, the phase-transfer approach allows generation of structures with multiple cores via simply tuning the rates of flow, whereas the coalescence approach is more suitable for generation of structures with a single core.

First, we focus on the phase transfer approach (Figure 10b). In this case, the number of cores in each generated shell can be roughly estimated as the product of the volume of the generated shell and the concentration of the beads in the suspension (set by their volume fraction) upon their reinjection on chip. However, at low volume fractions, due to the inhomogeneity of the suspension at the microscopic level, the actual volume fraction at the microfluidic junction strongly fluctuates. This leads to the randomness in the distribution of cores in the generated shells, i.e., to the polydispersity of the generated core–shell structures^204,246^ (Figure 10b). Accordingly, the yield of a given structure (i.e., with a given number of cores) is relatively low. Yet, the monodispersity can be improved via increasing the volume fraction of the beads up to the close-packing limit. In such a case, the steric repulsions between the beads lead to their ordering inside the microfluidic channel. As a result, the number of the re-encapsulated cores becomes strongly peaked around the most probable value (Figure 10b). It has been shown that one, two, or three hydrogel cores can be encapsulated in aqueous shells, with probabilities exceeding 90%.^63,289,343^ Such an approach has been extended also to generation of structures with two different types of cores.^343^ However, those results have been achieved using polyacrylamide or poly(*N*-isopropylacrylamide) (pNIPAAM) microbeads of sizes typically below 100 μm, which at sufficiently high (yet moderate) rates of flow readily rearrange and pack into ordered arrays and can be injected inside microchannels without the risk of clogging. However, because polyacrylamide as well as pNIPAAM precursors (containing acrylamide) are cytotoxic, these types of microgels are avoided in microtissue engineering. Also, it is not clear if the bead-packing and re-encapsulation strategy could be directly applied to other types of microgels, in particular to those with more “sticky” surfaces or to those with particle sizes beyond 200 μm.^204,246^ So far, the most promising results were obtained in the case of alginate beads generated using the “H+” method (this method yields very regular microgels of high sphericity) and re-encapsulated at a coflow junction. In this case, reproducibility of core–shell structures with a single core has been reported to reach up to 78% (versus empty or multicore structures).^204^ However, in the case of protein-rich ECM-like hydrogel beads such as gelatin, Matrigel, or GelMa beads, the re-encapsulation approach has been exercised to a very limited extent.^246^

Next, we turn to another approach to re-encapsulation based on coalescence of the core and the shell (Figure 10c(i)) as proposed in a series of works by Carreras et al.^109,185,310^ The authors used two independent droplet generators connected in series and separated by a long channel, such that the droplets generated at the first junction (“cores”) cross-linked via internal H^+^-triggered calcium release before reaching the second junction, where they coalesced with another alginate droplet (“shell”), resulting in an all-alginate core–shell capsule with a single core. The method integrated the whole workflow on a single chip which allowed skipping the intermediate washing steps and prevented potential problems with channel clogging upon reinjection. However, the efficiency of the encapsulation, e.g., in terms of the available throughputs and yield (fraction of nonencapsulated beads), has not been reported. One might expect that the generation of droplets at the two junctions must have been ideally synchronized. Such synchronization imposes some restrictions on the applicable rates of flow, and as such on capsule morphology (shell thickness etc.).

The coalescence-based re-encapsulation has been also applied to fabricate Janus rods.^84^ In this case, Matrigel plugs generated at a T-junction partially cross-linked before reaching the second T-junction also supplying Matrigel plugs. After coalescence, the plugs remained confined in a narrow tubing (Figure 10c(ii)), resulting in a Janus-like topology. It is noteworthy, in this case, that the final topology was dictated by the confinement rather than by the kinetics of cross-linking or by the values of the interfacial tensions.

The effect of confinement and partial coalescence was also exploited in a recent work by Samandari et al.,^344^ who encapsulated several hydrogel droplets inside a drop of oil. As the droplets gradually cross-linked, they also partially coalesced. The onset of coalescence was explained in terms of the depletion of the surfactant from between the solidifying droplet interfaces while nonmerging of droplet volumes in terms of the elastic response of the hydrogel matrix. Overall, the partial coalescence resulted in the arrested “budded” microgel morphologies with well-defined nonmixed compartments (Figure 10d). As the oil phase dewetted the microgel surfaces, the coalesced microgels spontaneously migrated outside the oil droplets into the external aqueous phase.

Finally, compartmentalized microgels were also generated using triple-emulsion droplets as templates.^345,346^ The strategy relied on W/O/W/O (water in oi in water in oil) emulsions consisting of droplets with a aqueous hydrogel core and an aqueous hydrogel shell separated by a thin oil layer (Figure 10e). After generation of the compound droplets, the hydrogel core and shell were cross-linked; the film of oil spontaneously ruptured and dissolved in the external oil with added solvent such as isopropyl alcohol (IPA).^345^ The use of IPA in the oil-removal step remains problematic in terms of cell encapsulation because of cytotoxicity of IPA. However, provided a more biocompatible method of oil removal, the strategy would have some advantages over other methods of formulation of core–shell structures, e.g., the possibility of independently controlling the composition and volume of the core and the shell phases. Noteworthy, the method provides high level of sphericity of the microgels, which often remains problematic in other methods^107,253,306^ (see section 4.4.4).

In summary, there are multiple different approaches toward reproducible generation of microgels with multiple compartments. However, generation of structures with an arbitrary (possibly large) number of compartments from arbitrary hydrogel materials remains a challenge, while also a bottleneck in fabrication of more complex (beyond single-core) hydrogel structures. Noteworthy, successful fabrication of such structures could open the way to topological meso-gels consisting of tens or hundreds of internal compartments. In fact, the structures built of hundreds of hydrogel droplets have been already domonstrated using droplet bioprinting,^88^ where the droplets settled under gravity and adhered to each other one-by-one. Such an approach, despite great success in fabrication of milimeter-scale organoids,^88^ is strongly limited in terms of precision and throughput. One could imagine that reproducibilty and rate of generation of compartmentalized mesostructures could be multifold increased via the use of microfluidics and in such a case significantly advance the emerging field of tissue engineering at the mesoscale.

#### 4.4.3 Porous Microparticles

**Figure 11:**
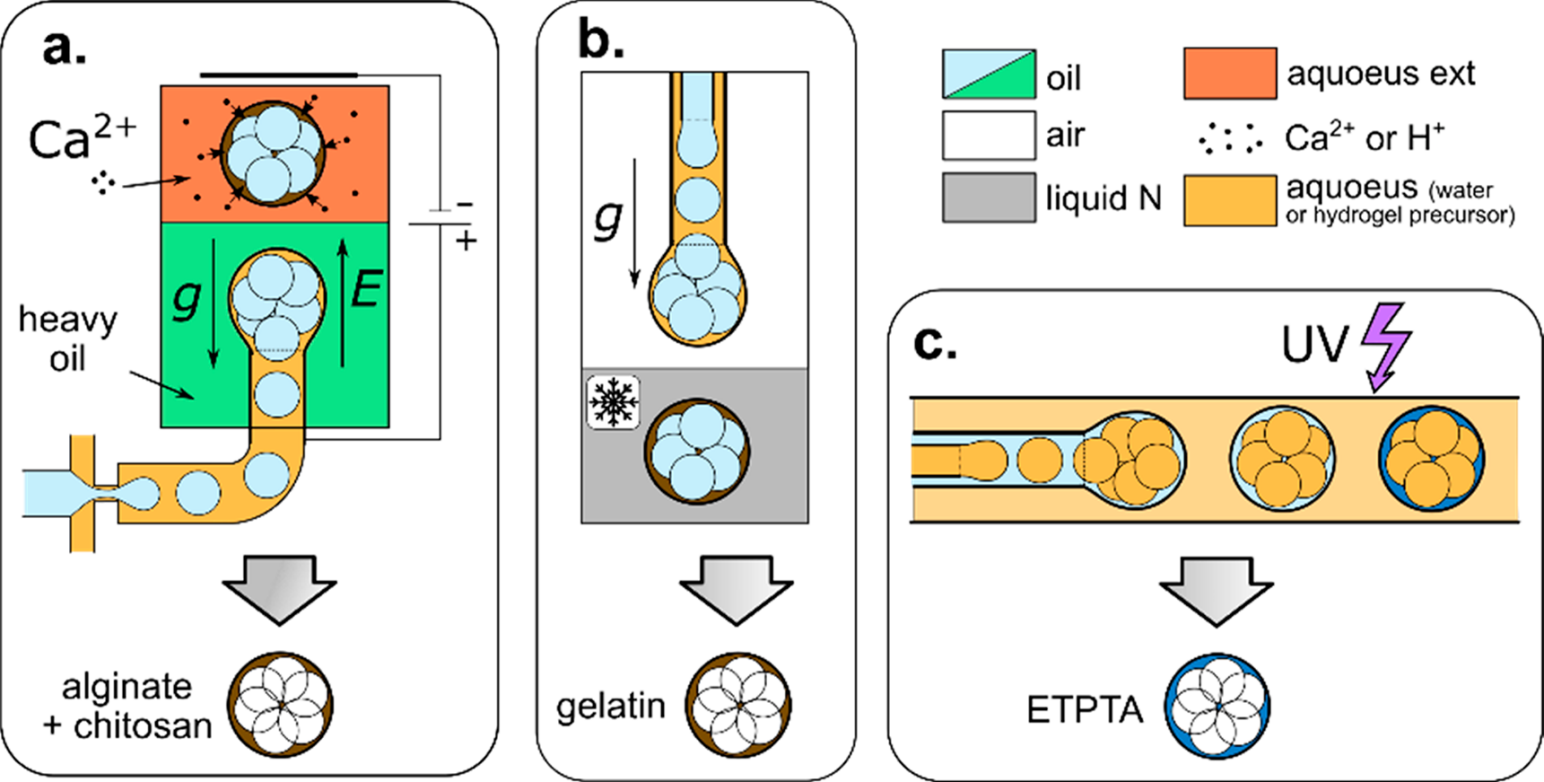
Microfluidic formulation of porous beads. (a) Generation of alginate–chitosan porous beads via electric-field-assisted dripping of O/W emulsion into the external fluorinated oil phase (FC40).^66^ The drops, carried by buoyancy, cross-link upon contact with a calcium bath placed above the oil. Cross-linking of chitosan with genipin is performed off-chip, followed by extraction of the inner oil phase. (b) Generation of gelatin porous beads via gravitational dripping of oil-in-gelatin emulsion into a liquid nitrogen bath.^65^ (c) One-step generation of porous ethoxylated trimethylolpropane triacrylate (ETPTA) beads via on-chip UV cross-linking. Water with surfactant is used as the outer and the inner phase.^64^

The engulfing core–shell double-emulsion-like topologies with multiple cores have been exploited as templates in the generation of porous microparticles. In tissue engineering, porous structures are used as scaffolds for cell seeding. This means that cells are not directly embedded in the ECM-like hydrogel matrix but rather attach to the inner surface of the macropores of the scaffold. Accordingly, porous scaffolds have some advantages over the ECM-like scaffolds such as less constrained reaction conditions (those can be harsh since cytotoxicity issues are irrelevant at the fabrication stage, i.e., before cell seeding), and a wider range of applicable biomaterials. The number of the inner pores and the size of interconnections between the pores in the structures templated from close-packed emulsions is known to depend on the volume fraction of the droplets^339^ as well as on the concentration of surfactant in the continuous phase.^347^ Generation of double-emulsion drops is typically carried out off-chip via buoyancy-,^66^ gravity-,^65^ or via electric-field-assisted^66^ dripping (Figure 11a,b, see also Figure 29E). Such methods lead to the number of cores in the range *n* = 10–100. However, smaller beads with *n* = 1–6 can be also generated via on-chip double-emulsification, e.g., using concentric capillaries^64^ (Figure 11c).

Various solidification mechanisms of the shell phase (as the one templating the porous microstructure) have been explored. The drops were cross-linked via ionic cross-linking in the calcium bath,^66^ via thermal quench in liquid nitrogen,^65^ or via exposure to UV light.^64^ In the strategies involving a hydrogel as the shell phase and oil (such as cyclohexane^66^ or toluene^65^) as the core phase, the oil is eventually extracted, e.g., using dimethyl sulfoxide^66^ or ethanol.^65^ In other approaches exploiting organic acrylates as the cross-linkable shell phase, the cores may be aqueous and not need to be removed.^64^ The latter approach allows direct use of the cross-linked beads in cell culture without any additional washing steps.

#### 4.4.4 Templating Based on the Nonequilibrium Liquid Architectures

Coalescence of miscible hydrogel-precursor liquid phases A and B leads to their mixing, whereas the A–B interface gradually vanishes. Accordingly, a sufficiently rapid cross-linking mechanism, capable of quenching of the transient nonequilibrium (nonmixed) liquid architectures, is required to fabricate compartmentalized microscaffolds. Several basic topologies have been demonstrated using such quench-assisted cross-linking including core–shell structures with solid shell/liquid core or solid shell/solid core as well as Janus structures.

First, we focus on the formulations involving calcium alginate, perhaps the most common hydrogel in microfluidics-assisted synthesis of compartmentalized microgels. In the case of core–shell structures, the core phase can be doped with a softer ECM-like biopolymer such as Matrigel, fibrin, or PEG-fibrinogen or even remain liquid to support fast cell aggregation.^217,230^

**Figure 12:**
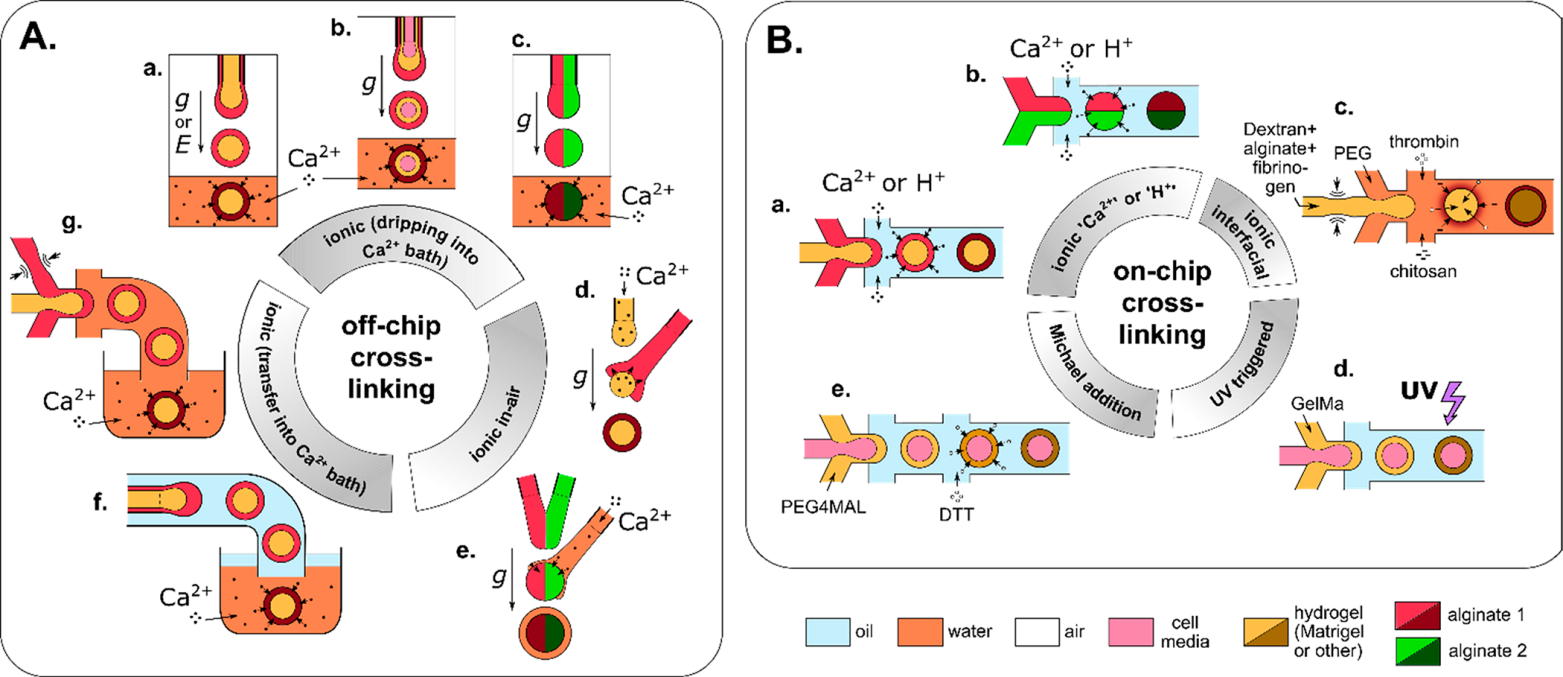
Microfluidic formulation of compartmentalized microgels based on quenching (rapid cross-linking) of nonequilibrium liquid architectures. (A) off-chip and (B) on-chip cross-linking strategies. (A) (a–c) Ionic cross-linking of compound droplets via gravitational, centrifugal, or electric-field-induced dripping into a calcium bath, resulting in core–shell^62,65,77,108,182,189,190,300,301^ structures with an alginate shell and (a) soft-hydrogel core or (b) media-filled core, or in (c) Janus^75,77,89,176,205^ structures. (d). In-air coalescence of calcium droplets with an engulfing alginate jet resulting in high-throughput generation of core–shell structures. (e) In-air coalescence of alginate Janus droplets with an engulfing calcium jet resulting in Janus structures.^199^ (f,g) Direct transfer of alginate core–shell droplets (via tubing) into a calcium bath^121,340^ prevents droplet deformation upon impact into a bath (a–c). (B) (a,b) Ionic on-chip shell cross-linking via Ca^2+^^95,107,306^ or H^+^^175,184,271,309^ ions supplied in the external phase, resulting in (a) core–shell or (b) Janus structures. (c) Interfacial cross-linking in an all-aqueous system via complexation of alginate in the droplet phase with chitosan in the external phase, resulting in ultrathin-shelled structures capsules.^217,275^ Dextran and PEG are added in the respective two phases to support the formation of droplets. Fibrinogen, added to the droplet phase, gradually cross-links as the cross-linking enzyme thrombin diffuses inside the droplets from the external phase. (d) Rapid UV cross-linking of core–shell droplets with a GelMa shell, resulting in a solid-shell/liquid-core structure.^253^ (f) Michael-addition type cross-linking of core–shell droplets with a PEG derivative containing shell (e.g., PEG4MAL, maleimide functionalized PEG^265^) triggered by thiol molecules, e.g., dithiothreitol (DTT),^265^ supplied in the oil phase.

In general, the formulation of microcapsules via ionic cross-linking can be divided into methods in which the hydrogel precursor droplets are cross-linked off-chip (Figure 12A) or on-chip (Figure 12B).

**Off-chip** cross-linking typically relies on the use of two concentric needles (or concentric capillaries) in which the inner needle supplies a nonalginate soft-hydrogel or a liquid core phase, whereas the outer needle supplies a rapid-cross-linking alginate shell phase. The core–shell droplets are formed via gravitational-,^65,205,300^ centrifugal-,^62,176,189^ or an electric-field^47,77,111,120,182,190,301^ assisted dripping (Figure 12A(a–e)) and subsequently coalesce with a calcium bath, which causes immediate cross-linking of the alginate shell. An additional hydrogel layer, e.g., Matrigel, can be added between the core and external shell phases as a cell-adhesive coating at the inner surface of the shell^108^ (Figure 12A(b)). Dripping strategy has also been applied to generate **Janus** alginate microcapsules (Figure 12A(c)). In such a case, the two alginate phases A and B have been supplied in parallel to the tip of a needle via dedicated connectors^75^ or via a Y-junction^205^ or otherwise coejected using a two-barrel (or a multibarrel) capillary^77,89^ or a theta-shaped capillary.^176^ The dripping has also been realized via mechanically induced (via high-frequency actuator) Rayleigh–Plateau instability of alginate or calcium jets in air^199,303^ (Figure 12A(d–e)). For example, core–shell structures have been generated by encapsulating calcium droplets within alginate shells^303^ (Figure 12A(d)), while Janus structures have been achieved via merging the jets A and B generated by two independent nozzles before their breakup into Janus droplets, followed by coalescence with a calcium jet generated with a third nozzle^199^ (Figure 12A(e)).

It is noteworthy that the transfer of droplets to a calcium bath has also been realized via tubing. This approach has been exercised using either oil^121^ (Figure 12A(f)) or aqueous media as the carrier phase, in the latter case with droplets generated via mechanical pulse-induced dripping applied with an external actuator^340^ (Figure 12A(g)).

Alginate shells in compound microdroplets have also been cross-linked **on-chip**, with calcium ions Ca^2+^ dissolved in the external oil (Figure 12B(a)) via the use of a solvent such as 2-methyl-1-propanol^306^ or via dispersion of aqueous calcium chloride solution in the form of a nanoemulsion in the oil phase.^95,107^ Alternatively, calcium ions can be trigger-released directly from the shell phase. The latter strategy is realized via addition of a nondissociated calcium compound (e.g., Ca-EDTA or CaCO_3_) to the alginate phase and an organic acid (e.g., acetic acid) to the continuous oil phase. When both phases come in contact, the organic acid dissociates at the shell–external interface, releasing H^+^ cations (Figure 12B(b)), which react with the calcium compound and cause release of calcium ions, which in turn gradually cross-links the shell.^175,184,309^

In both of the above on-chip cross-linking methods (“Ca^2+^” or “H^+^”), the core can remain liquid^175,306,309^ or become cross-linked. Hydrogels such as Matrigel^184^ or collagen^95^ readily cross-link upon placing the microparticles in 37 °C for culture. Alternatively, cross-linking of the shell and the core can happen simultaneously upon generation of droplets of alginate–dextran mixed with fibrinogen in the external aqueous PEG-rich phase with added chitosan and thrombin (Figure 12B(c)), where the interfacial complexation of alginate and chitosan results in an ultrathin shell, whereas thrombin diffuses into the core to cross-link fibrinogen.^217,275^

Finally, the H^+^-triggered on-chip cross-linking mechanism has also been applied to fabricate Janus microgels^271^ (Figure 12B(d)). We note that the H^+^-triggered microdroplet gelation method has an important advantage over other ionic cross-linking approaches in that it provides homogeneous gelation in the whole droplet volume, which results in highly spherical hydrogel beads. This contrasts with the all-aqueous systems in which the extremely low interfacial tension and the external viscous forces can lead to distortions of the shape of the alginate droplets.^108,340^ A possible disadvantage of the H^+^-triggered method is that the drop in pH caused by H^+^ release may have a negative effect on cell viability;^311^ a potential wayaround has been recently proposed by Liu et al., who added HEPES to the shell phase, which then served as a pH barrier protecting the cells.^309^

Besides ionic cross-linking, UV cross-linking has been also applied to solidify compartmentalized hydrogel droplets such as core–shell structures. PEGDA,^348^ or GelMa have been used as the shell phase^253^ (Figure 12B(e)), resulting in microcapsules with a liquid core. Janus PEGDA structures have also been solidified with UV light,^349^ but the utility of the PEGDA microparticles in microtissue engineering can be in general questioned due to the reported poor cell survival and low cell proliferation rates.^350^ Finally, on-chip cross-linking of the hydrogel shell has also been demonstrated^265^ with PEG derivatives such as maleimide functionalized PEGs (PEG4MAL) cross-linking via Michael-type addition reaction triggered by dithiothreitol (DTT) molecules supplied in the oil phase (Figure 12B(f). Due to the longer time of cross-linking and the associated dilution of PEG in the liquid core, this method generated capsules with thin shells (around 10–50 μm thick). The use of fluorescently labeled PEG (FITC-PEG-SH) revealed a diffuse core–shell interface, indicating possible gradient in cross-link density, which in turn was ascribed to the diffusion or partial mixing of the core and the shell compartments. The mixing was reduced by adding 35 kDa PEG at 8% w/v concentration to the core phase.

#### 4.4.5 Throughput-Limiting Factors

In the case of microfluidics-assisted synthesis of hydrogel microbeads, the throughput-limiting factors, in general, may be associated not only with the frequency of droplet generation^351^ but also with the rate of droplet cross-linking.^199,303^ If the cross-linking reaction is triggered “internally”, i.e., via a reaction happening inside the droplet independently of the external environment (such as in the enzymatic, Michael addition, or click chemistry cross-linking reactions, see sections 4.3.2 and 4.3.3), the droplets can be incubated outside the chip, which effectively decouples droplet generation and cross-linking.^351^ However, the internally triggered cross-linking cannot be directly used for one-step generation of microgels with multiple internal compartments (yet, two-step methods are possible, as already reviewed in section 4.4.2). For this purpose, the more rapid “external” cross-linking strategies are required, such as ionic cross-linking (see previous section 4.4.4). In fact, ionic cross-linking has been recently applied to generate the internally structured microgels, such as core–shell or Janus beads, at frequencies of the order 10^5^ Hz via solidification “on the fly” using calcium jet supplied directly to the droplet upon their transit (in air) between the droplet generator and a collection chamber^199,303^ (Figure 12A(d,e)).

### 4.5 Generation of Compartmentalized “1D” Structures

In general, the strategies of formulation of “0D” microbeads can also be adapted to formulation of “1D” microfibers via simply changing the mode of operation of the microfluidic junction from dripping to jetting. However, because jets do not represent an equilibrium morphology, complex architectures can only be achieved via their kinetic quench. This is typically achieved via ionic cross-linking, and in this section we focus on ionically cross-linked compartmentalized alginate microfibers. In particular, generation of core–shell microfibers consisting of a rigid alginate shell and an ECM-like soft-hydrogel core, e.g., Matrigel,^106,193,261,262^ collagen,^45,106,112,189,195^ fibrin,^110^ or GelMa,^197^ or a liquid core^114,208,304^ can be achieved via cross-linking of a core–shell jet generated, e.g., using concentric needles or capillaries with the alginate phase supplied to the outer capillary and the ECM phase to the inner capillary. Similarly, the use of parallel needles or capillaries leads to the Janus microfibers. In either case, such type of strategies always lead to transversally patterned microfibers. Longitudinally patterned microfibers have also been demonstrated, e.g., via embedding droplets^60,207,352,353^ or hydrogel beads^221^ or via longitudinally varying the fiber diameter.^354^ The different protocols of generation of the variety of alginate microfiber morphologies have been recently also reviewed by Yu et al.^59^

#### 4.5.1 Transversally Patterned Microfibers

**Figure 13:**
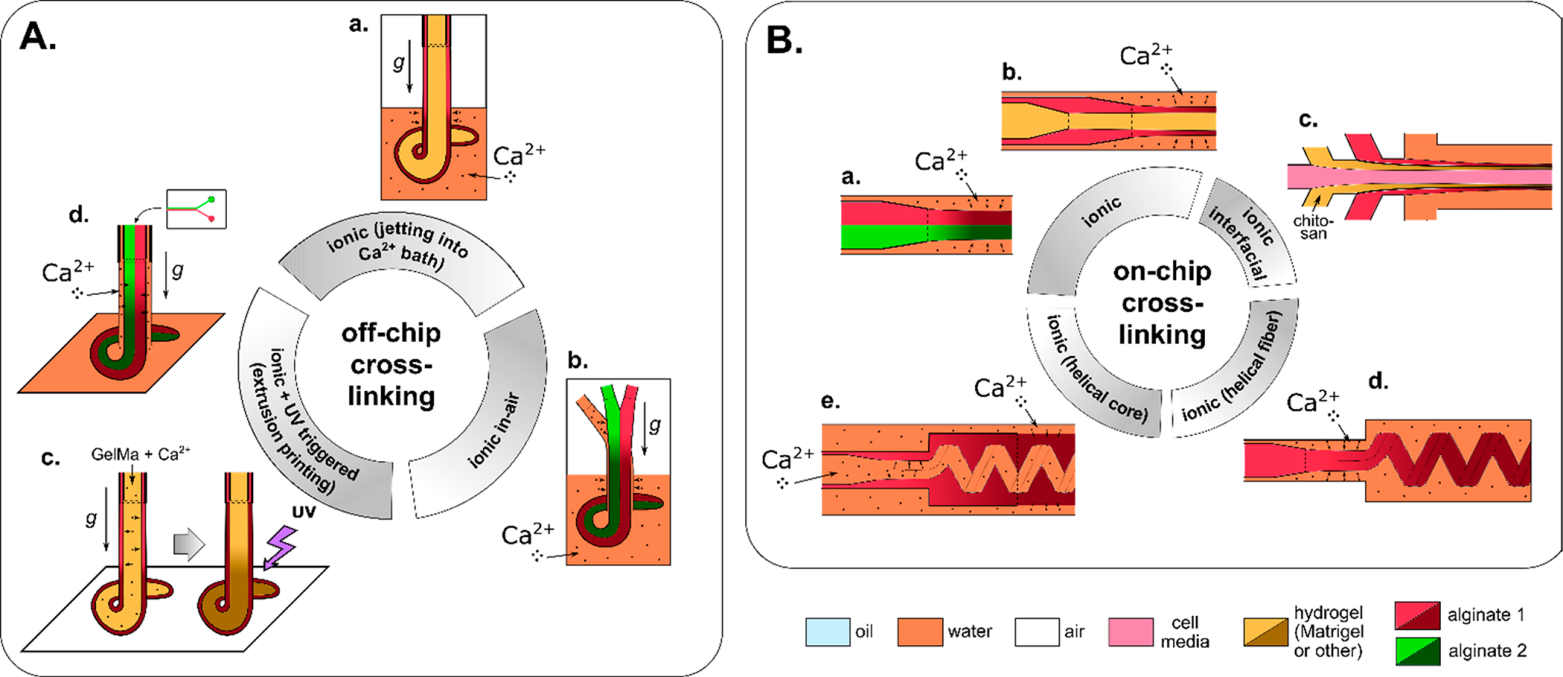
Microfluidic formulation of compartmentalized hydrogel microfibers. (A) off-chip and (B) on-chip strategies. (A) (a) Coaxial jetting of alginate (shell) and soft-hydrogel (core) into a calcium bath.^114,189,255,262^ (b) Janus alginate jet cross-linking via coalescence with a calcium jet.^199^ (c) Extrusion printing of a core–shell jet with an alginate shell and a GelMa core with calcium ions supplied in the core phase, followed by UV cross-linking.^197^ (d) Extrusion of a Janus jet using a coaxial needle with calcium supplied to the outer needle.^73,203^ (B) (a,b) On-chip cross-linking of Janus^67,79,82^ and core–shell jets^29,45,67,74,79,80,146,193,202,208,253,255,261,355^ via coflowing outer calcium phase. (c) Generation of ultrathin shelled fibers via interfacial complexation of coflowing sodium alginate and chitosan jets.^304^ (d,e) Generation of (d) a helical fiber^208,210,340^ and (e) a fiber with helical core^208^ in concentric capillaries with stepwise increasing dimeter of the (d) outer or (e) middle capillary.

In the case of fibers templated from jets consisting of several coflowing subjets, the fiber cross-linking strategies can be divided into those performed off-chip (Figure 13A) and on-chip (Figure 13B).

**Off-chip** cross-linking is typically performed via jetting directly into a calcium bath^114,189,255,262^ (Figure 13A(a,b)) or via extrusion onto a substrate^73,112,197,203^ (Figure 13A(c,d)). All-alginate Janus fibers can also be generated by merging of two different alginate jets in air, followed by their coalescence with a calcium jet^199^ (Figure 13A(b)) or via using a Y-junction,^203^ followed by coextrusion with a calcium solution using a concentric needle^73,203^ (Figure 13A(c)). In the latter case, cross-linking is initiated at the tip of the needle directly upon extrusion when the inner alginate and the outer calcium streams come in contact. Additional doping of alginate with GelMa followed by UV exposure can be applied to allow gradual dissolution of alginate in cell media^203^ without losing hydrogel integrity, thus providing softer hydrogel, optimal for cell culture. Another extrusion-based strategy aimed at generation of core–shell fibers relies on supplying the calcium ions directly inside the core phase (Figure 13A(d)), such that the gelation of the shell is triggered directly upon extrusion.^197^ In this case, the core phase consists of a GelMa precursor, which may be later also cross-linked via UV exposure.

In **on-chip** cross-linking, calcium ions are delivered to the alginate jet directly via the coflowing external phase. The strategy has been used to fabricate Janus,^67,79,82^ core–shell,^45,67,74,79,80,208,255,355,356^ and hollow^67,79,80,146,202,208,255^ microfibers (Figure 13B(b,c)), including microfibers with ultrathin shells^304^ (Figure 13B(c)). In the latter case, chitosan and alginate were separately supplied as the inner-shell and outer-shell phases, and the cross-linking was achieved via interfacial complexation between the two phases.^304^ The method led to extremely thin shells, of thicknesses presumably in submicrometer range, which were however too delicate to handle. To achieve slightly thicker, yet still ultrathin shells, calcium ions were added to the external phase, which additionally triggered alginate cross-linking. In this case, the complexation also occurred, however, without advection, resulting in a thin alginate–chitosan layer. Final dissolution of calcium alginate in the external PBS finally resulted in core–shell fibers with alginate–chitosan shells of thicknesses in the range 1–10 μm, suitable for handling and long-term culture with excellent permeability properties, facilitating nutrient/waste exchange between the encapsulated cells and external media.

Unique fiber morphologies have been achieved via the use of a concentric capillary with step-like increase in the diameter of the outer capillary^208,210^ (Figure 13B(d,e)). The flow of the external phase abruptly decelerated at the step which led to longitudinal compressive viscous stresses acting on the inner jet resulting in jet coiling. The spiral-like jet morphology was quenched via ionic cross-linking, resulting in spiral microfibers or core–shell microfibers with a spiral core, respectively. The structures have been proposed to mimic a special kind of “curly” vessel responsible for creating swirling blood flows (see section 5.2.7).

**Figure 14:**
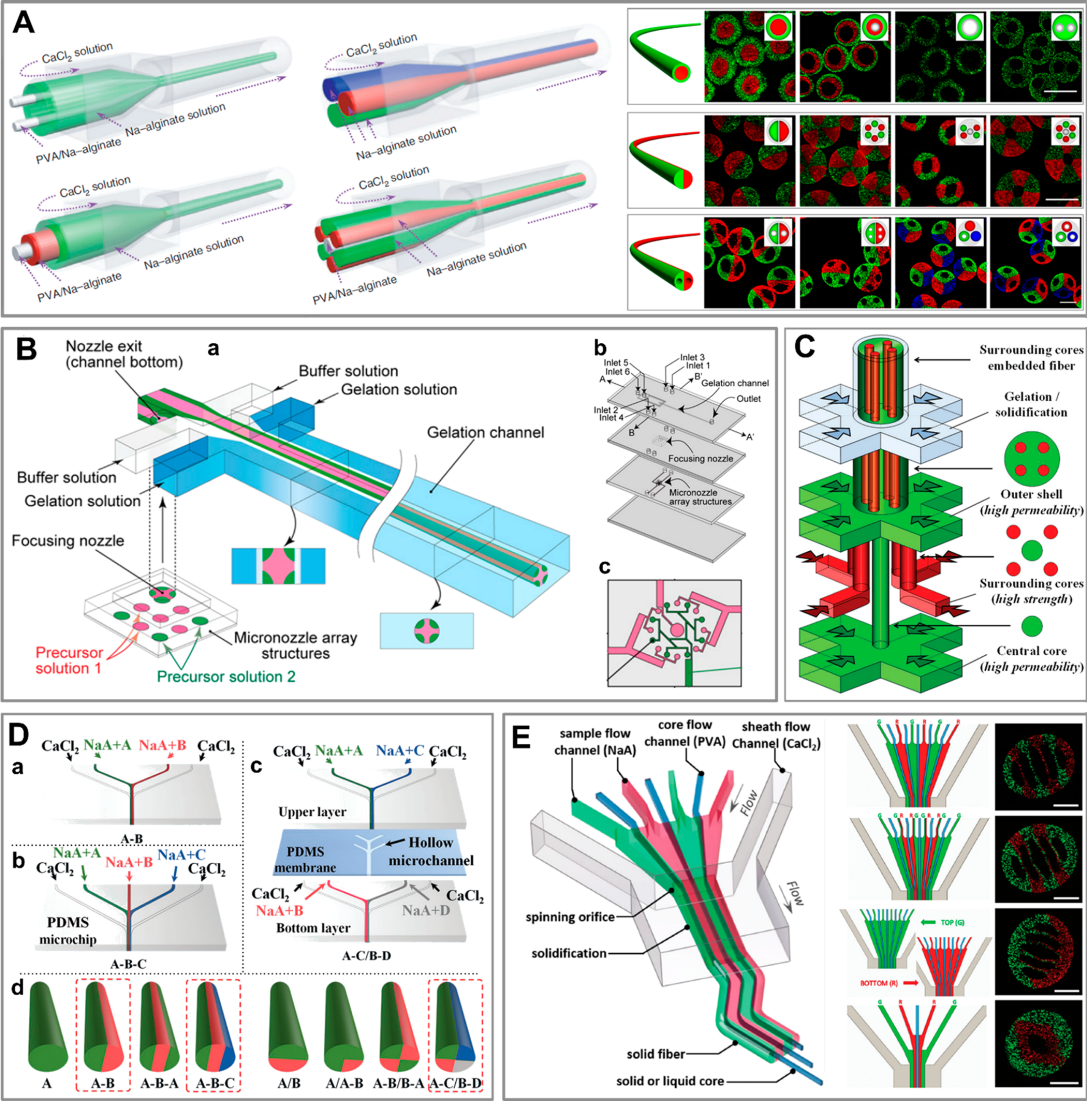
Microfluidic formulation of complex transversally patterned fibers. (A) Generation of complex core–shell, Janus, and hollow alginate fibers with the use of multiple coaligned inner capillaries inside an outer capillary supplying calcium coflow. Scale bar is 250 μm. Adapted with permission from ref (67). Copyright 2018 Nature Publishing Group. (B) (a) Generation of grooved Janus fibers via integration of micronozzle array with a coflow channel. (b) Scheme of the multilayer chip design. (c) Layout of 16 nozzles for generation of grooved fibers with 8 grooves. Adapted with permission from ref (274). Copyright 2018 IOP Publishing Ltd. (C) Integration of multiple aligned and nested nozzles for generation of core–shell fibers with multiple hollow “pockets” (red channels). Adapted with permission from ref (357). Copyright 2018 Wiley. (D) (a–d) Planar (a,b) two-layer, and (c) three-layer microfluidic devices for generation of (d) fibers with 2, 3, or even 4 independent longitudinal compartments. Adapted with permission from ref (80). Copyright 2020 Wiley. (E) Microfluidic chip with multiple parallel inlets of different depths supplying alginate and water for generation of compartmentalized multi-Janus multihollow fibers. Scale bar is 100 μm. Adapted with permission from ref (69). Copyright 2016 Wiley.

Finally, the most complex, tailorable alginate fiber morphologies have been demonstrated using coaligned nozzles or nozzle arrays (Figure 14). For example, multiple coaligned capillaries^67,79^ supplied with up to three different alginate phases were used to generate hollow double-shell fibers, multihollow fibers, multi-Janus fibers, hollow multi-Janus fibers, and multihollow Janus fibers (Figure 14A). Another approach, proposed by the Seki group,^206,274^ relied on integration of multiple alginate streams via the use of a three-layer microfluidic device with the consecutive layers of the device consisting of a micronozzle array, a focusing nozzle, and a coflow geometry (Figure 14B), respectively. The device generated alginate Janus fibers, with multiple compartments arranged along the surface, thus forming longitudinally grooved fibers, which served, e.g., as a substrate for guiding intercellular linear network development in neuron culture.^274^

An analogical approach has been later also used to generate core–shell fibers with hollow subcompartments (Figure 14C), resulting in fibers with cross-sectional tunable flower-like morphology.^357^ Merging of up to four different alginate streams has been also demonstrated^80^ via 3D coflow in a three-layer device, resulting in Janus fibers with compartments aligned side-by-side or forming a pie-chart-like pattern with up to four different compartments (Figure 14D). Yet another approach has exploited integration of multiple streams in-plane,^69^ resulting in alginate structures intermediate between a fiber and a ribbon, with up to 11 parallel compartments including hollow sections (Figure 14E).

#### 4.5.2 Longitudinally Patterned Microfibers

**Figure 15:**
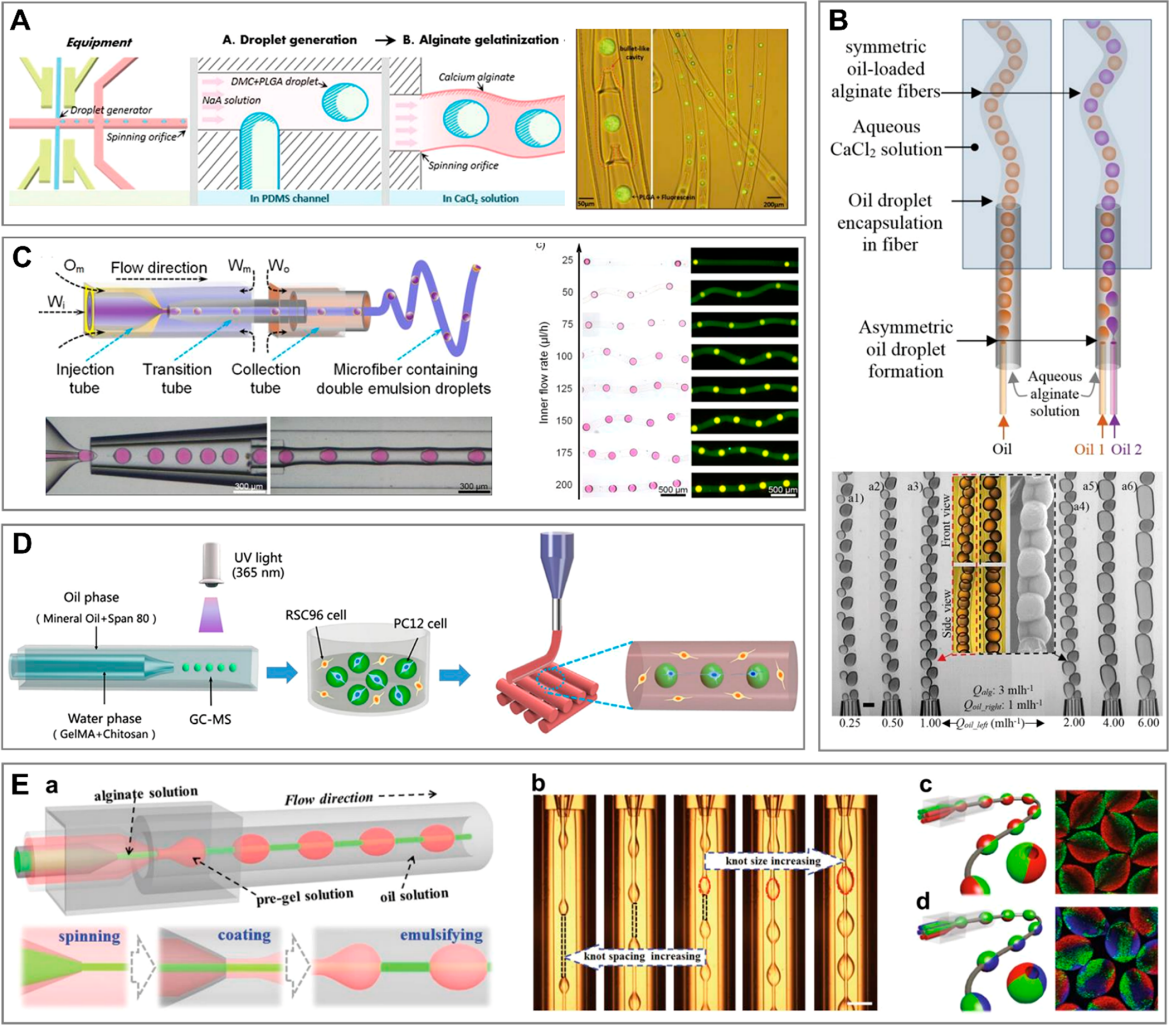
Microfluidic formulation of longitudinally patterned fibers. (A) Generation of alginate fibers with encapsulated fluorescein-doped polylactic-co-glycolic acid (PLGA) spheres (obtained from PLGA-rich dimethyl carbonate droplets) via wet spinning. Scale bars are 50 μm (left) and 200 μm (right). Adapted with permission from ref (60). Copyright 2014 Wiley. (B) Similar oil-in-alginate structures generated via buoyancy-assisted extrusion. Note various morphologies depending on droplet packing inside the fiber. Scale bar is 200 μm. Adapted with permission from ref (352). Copyright 2016 Wiley. (C) Encapsulation of aqueous droplets in alginate fibers via double-emulsion approach. Adapted with permission from ref (207). Copyright 2018 American Chemical Society. (D) Extrusion 3D printing of GelMa fibers loaded with encapsulated precross-linked GelMa microbeads. Adapted with permission from ref (221). Copyright 2020 Elsevier. (E) (a) Rayleigh–Plateau instability of the outer jet in a coflowing system can be exploited toward generation of spindle-knotted fibers with alginate core and UV-curable “knots”. (b) Size and spacing of the knots can be controlled via tuning the rates of flow. Adapted with permission from ref (354). Copyright 2016 Wiley. (c,d) The method can also be used to generate fibers with transversally patterned “multi-Janus” GelMa knots. Adapted with permission from ref (201). Copyright 2017 Science China Press and Springer-Verlag.

In devices based on two nested junctions, with the inner and outer junctions operating in the dripping and jetting modes, respectively, the ensuing structure is a “1D” droplet-in-fiber morphology (Figure 15). With droplets distributed evenly along the fiber, cross-linking of the fiber shell phase may serve to fix the spacing between the droplets, thus introducing additional level of control into multimicrotissue cell cultures.^68^ Also, the embedded droplets can be used to modify mechanical properties of the fibers.^352^

Alginate microfibers encapsulating evenly spaced oil droplets have been demonstrated by Yu et al.^60^ using integrated valves to generate the droplets and wet spinning to cross-link the microfiber (Figure 15A). In another work, Chaurasia et al.^352^ used nested capillaries to fabricate alginate fibers with two types of encapsulated oil droplets. Precise control over volume and arrangement of droplets was demonstrated (Figure 15B), whereas the mechanical properties of the fiber such as its tensile stiffness and strength were shown to depend on the droplet volume fraction. Droplet-in-fiber structures have also been generated by Deng et al.^207,353^ using a triple-emulsion approach in which the embedded droplets consisted themselves of an aqueous core and an oil shell. The control over droplet spacing over a wide range of interdroplet distances has been demonstrated (Figure 15C). Chen et al.^221^ fabricated GelMa microfibers with cross-linked inner GelMa droplets using a two-step approach consisting of generation and resuspension of GelMa beads in a GelMa precursor, followed by extrusion and UV-cross-linking of the compound GelMA-in-GelMa bioink (Figure 15D). A very recent work by Wang et al.^68^ additionally demonstrated the possibility of generation of all-hydrogel droplet-in-fiber morphologies via the use of two-phase aqueous systems in which the dextran-rich droplet phase can be suspended in the PEG-rich alginate continuous phase (see Figure 2A(i)) as well as Figure 24E).

Finally, capillary instability of the outer jet preceded by cross-linking of the inner jet has been exploited by the Zhao group^354^ using various types of coflowing core–shell systems. The resulting structures were the so-called spindle-knotted fibers with regularly spaced spindle-shaped droplets of GelMa strung along an alginate fiber (Figure 15E). The authors used a coflowing external oil phase to induce Rayleigh–Plateau instability of the outer GelMa jet and cross-linked the structure with UV light as the instability developed. A the same time, the alginate inner jet was cross-linked via diffusion of calcium ions added to the GelMa phase. Even though similar spindle-knotted structures were previously demonstrated in nonmicrofluidic systems, microfluidics improved the control over the spacing and size of the knots. The authors also demonstrated that the fibers can be used as cell carriers with adjustable linear density of cells (i.e., along the fiber) controlled via knot spacing. Later, the same group also demonstrated that the technology can be extended toward fabrication of the spindle-knotted fibers with transversally patterned Janus and multi-Janus knots.^201^

#### 4.5.3 “1.5D” Ribbon-Like Structures

**Figure 16:**
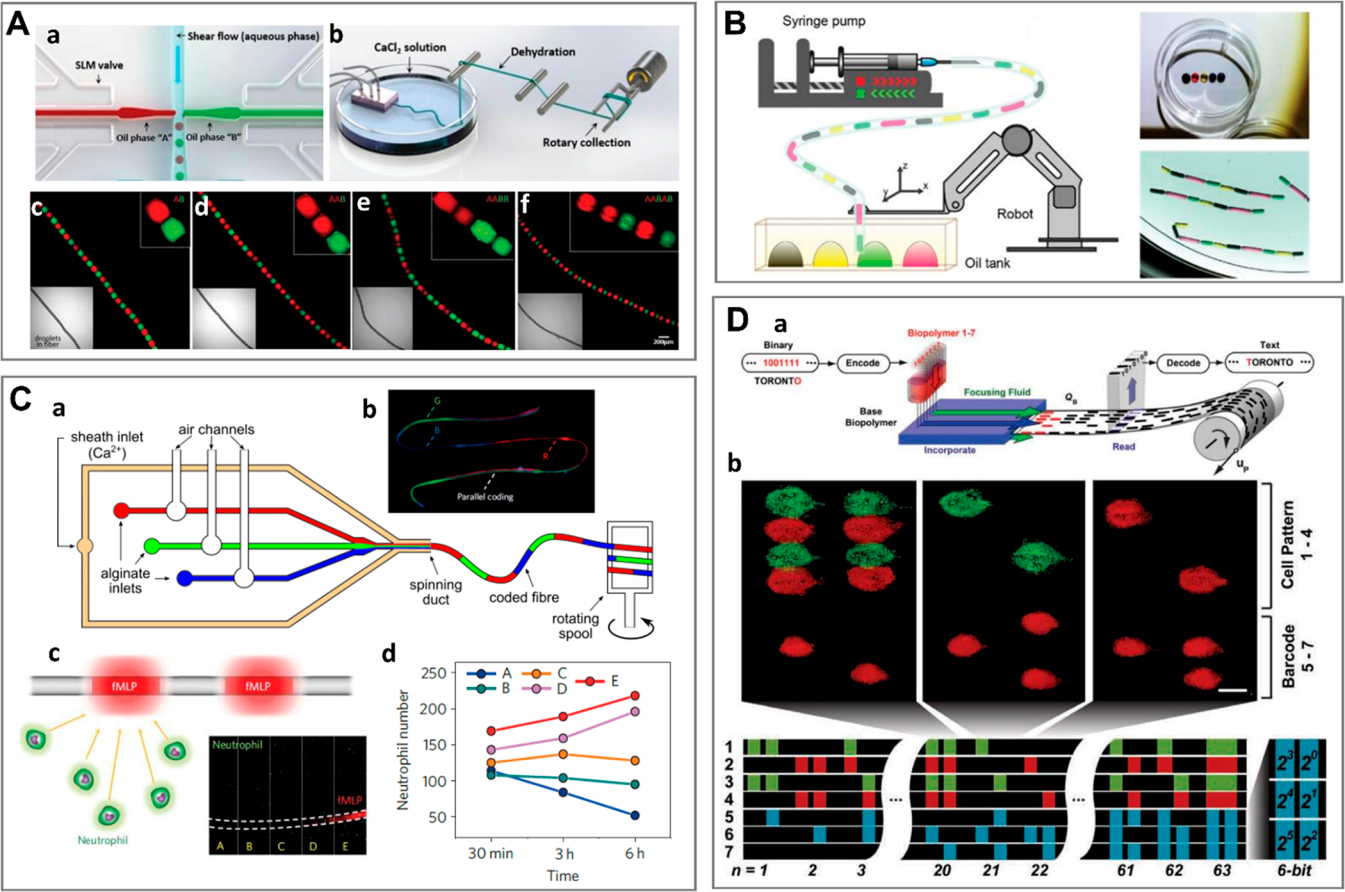
Microfluidic formulation of pattern-coded fibers and ribbons. (A) (a,b) Wet-spun alginate fibers with encoded periodic sequences of red- and green-dyed hexadecane droplets generated on demand using PDMS valves. (c–f) Examples of encoded structures. Scale bar is 200 μm. Adapted with permission from ref (60). Copyright 2014 Wiley. (B) Aspiration assisted printing of sequences of different GelMa plugs. The process is assisted by physical partial-cross-linking of the plugs via lowering the temperature to 4 °C prior to printing. Adapted with permission from ref (81). Copyright 2019 Royal Chemical Society. (C) (a) Generation of fibers with encoded patterns based on sequential delivery of different alginates to the spinning orifice with the use of pressurized valves. (b) Microfiber with longitudinal and lateral patterning. (c) Longitudinal patterning was used to generate regions with different concentration of a cell chemoattractant (fMLP). (d) Migration of neutrophils toward the region with highest concentration of fMLP was observed. Adapted with permission from ref (82). Copyright 2011 Nature Publishing Group. (D) (a) Generation of alginate ribbons patterned via “spotting” from seven parallel nozzles, each operating independently and controlled with an external valve. (b) Cell patterns and corresponding barcodes generated with a device. Scale bar is 500 μm. Adapted with permission from ref (59). Copyright 2012 Wiley.

Parallel arrangement of multiple alginate jets can be also used to generate hydrogel ribbons with transversal stripe-like patterning^83,212^ for applications in coculture studies^83^ or a substrate for muscle^212^ or neuron^211^ culture. For example, Leng et al.^59^ developed a multinozzle device for generation of all-alginate ribbons with lateral as well as longitudinal hydrogel patterning (Figure 16D), whereas Kang et al.^211^ used a slit-shaped nozzle with microfabricated “teeth” to impose longitudinal grooves at the surface of alginate ribbons. High-aspect-ratio alginate ribbons (around 2 mm wide and 50–100 μm high) were demonstrated by Kobayashi et al.,^83^ who integrated 64 inlets supplied with alginate with and without cells (the latter as spacers, see Figure 23C). More recently, Zhao et al.^212^ applied similar technique using interchanging softer and stiffer hydrogel precursors to fabricate ribbons with longitudinal grooves (Figure 28E).

#### 4.5.4 Complex Patterning and Information Coding in Hydrogel Fibers and Ribbons

So far, we only considered microfibers and ribbons which were either translationally invariant along the axial direction or consisted of simple periodic patterns formed by the embedded droplets or jets. A more complex combinatorial longitudinal patterning has been proposed by Yu et al.^60^ via embedding oil droplets consisting of two types of oil (hexadecane with two different dyes) supplied to the fiber in a predesigned sequence (Figure 16A). The droplets were generated on-demand using programmable actuators which allowed generation of a wide range of different periodic sequences, which suggested possible application in information coding (yet no specific application of such coding was demonstrated).

Another approach to the fabrication of microfibers with longitudinal hydrogel motifs was recently demonstrated by Ma,^81^ who used an automated aspiration-printing system to aspirate a sequence of hydrogel droplets from different hydrogel reservoirs (GelMa with different dyes) into tubing filled with an oil-phospholipid mixture, with lipids serving as a surfactant, and then to extrude the precross-linked droplets onto a substrate (Figure 16B). The precross-linked droplets partially coalesced as the oil drained from between them, resulting in fibers with longitudinal segments composed of the different aspirated hydrogels.^81^

Microfibers with longitudinal patterning have been also demonstrated by Kang et al. from the Khademhosseini group,^82^ who used programmable valves to achieve longitudinal patterning with three types of dyes (Figure 16C). The strategy relied on the use of continuous all-alginate fibers rather than on embedding droplets. The work demonstrated the possibility of linear patterning with a chemoattractant such as fMLP (formyl-Mat-Leu-Phe); the fiber was embedded in the external hydrogel matrix encapsulating living cells (neutrophiles), and chemotaxis was observed with cells migrating toward the patterned regions containing higher concentrations of fMLP.

Finally, Leng et al.^59^ developed wet-spun alginate ribbons with lateral patterning and used them to encode information about the local cellular content of the ribbon where the coding was realized via valve-based alginate spotting (Figure 16D). The ribbons consisted of seven “lanes”, with four lanes dedicated to generation of a cellular pattern and the remaining three to the generation of the “barcode”. The authors used two consecutive spots on each of the three coding lanes, resulting in 2 × 3 = 6-bit coding capacity, i.e., the possibility of generation of 2^6^ = 128 different barcodes. More recently, a similar alginate-sheet technique was used by the same group to fabricate hydrogel membranes for wound healing,^70^ allowing extrusion of a hydrogel wound dressing directly on the skin.

In summary, despite the interesting functionality, the capabilities of the linearly coded structures in tissue engineering or high-throughput screening seem to have not yet been fully exploited.

### 4.6 Generation of Compartmentalized “2D” Structures

**Figure 17:**
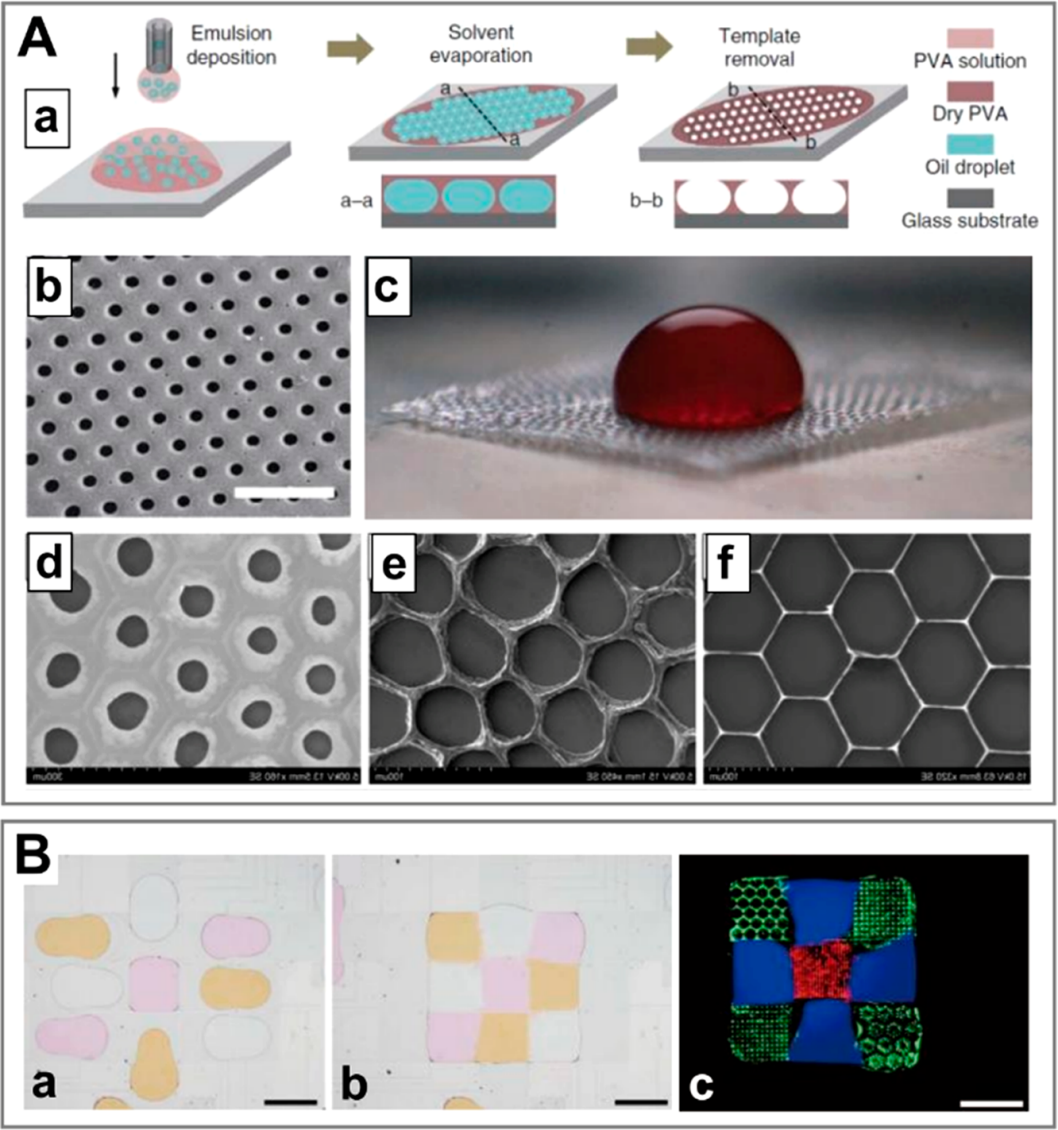
Microfluidics-assisted formulation of hydrogel sheets. (A) Porous membranes. (a) Schematic of generation based on deposition of a monolayer of microfluidic droplets or bubbles at a substrate. (b) SEM image of a PVA membrane (scale bar 100 μm). Adapted with permission from ref (359). Copyright 2017 Nature Publishing Group. (c) Drop of blood at a PVA membrane. Adapted with permission from ref (360). Copyright 2020 Wiley. (d–f. PEG/alginate membranes for alginate concentrations (d) 0.1 wt %, (e) 0.3 wt %, and (f) 0.5 wt %. Adapted with permission from ref (71). Copyright 2016 American Chemical Society. (B) (a,b) Generation of checkerboard hydrogel PEGDA sheets from precross-linked droplets via digital microfluidics. (c) Structure generated from droplets of several different hydrogels: PEGDA (green), polyacrylamide (red), and Matrigel (blue). Scale bar is 1 mm. Adapted with permission from ref (87). Copyright 2016 The Authors.

Compartmentalized 2D structures (Figure 17f) constitute a relatively small group of microfluidics-assisted tissue engineering scaffolds and include porous membranes,^71,358−360^ bottom-up assembled 2D microgel checkerboard patterns^87^ and 2D hydrogel droplet networks.^85^

The porous hydrogel membranes, generated from close-packed 2D arrays of microfluidic droplets or bubbles (Figure 17A), find applications as wound dressings because the interfacial porosity results in hydrophobicity^359,360^ and even omniphobicity,^359^ which lowers the probability of bacterial infections. At the same time, hydrogel dressings can be loaded with growth factors and/or antibacterial agents such as zinc compounds.^360^ Very recently, Chi et al.^361^ demonstrated porous “Janus” membranes with hydrophobic top surface and hydrophilic bottom surface for optimal drainage of exudate without undesired tissue adhesion.

The assembly of hydrogel checkerboard patterns have been demonstrated with digital microfluidics via bottom-up approach in which individual precross-linked hydrogel precursor droplets were brought together sequentially^87^ (Figure 17B). The structures were assembled from precross-linked PEGDA, Matrigel, and GelMa droplets via their one-by-one partial coalescence followed by post-cross-linking. Additionally, dielectrophoretic patterning of cells encapsulated inside the GelMa microgels was demonstrated without affecting cell viability.

**Figure 18:**
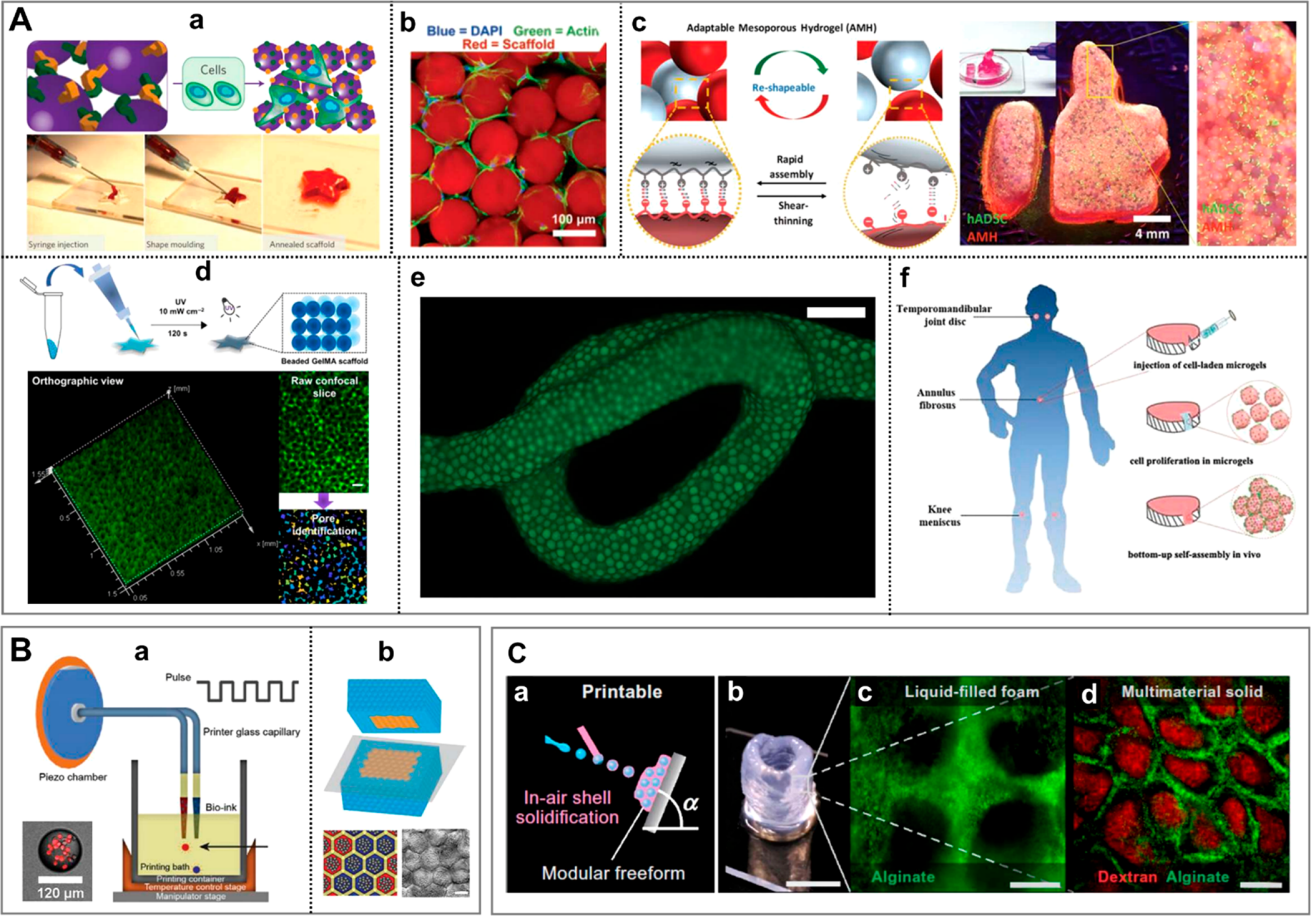
Microfluidics-assisted formulation of granular 3D structures. (A) (a) Injectable PEG beads anneal into a 3D scaffold via binding of interfacial complementary peptide motifs (orange and green). Adapted with permission from ref (156). Copyright 2015 Nature Publishing Group. (b) Microscopic view of the annealed scaffold seeded with cells. Adapted with permission from ref (351). Copyright 2019 Wiley. (c) Oppositely charged GelMa and ChitoMa beads formed adaptable mesoporous hydrogel (AMH), which was mixed with cells (here, human adipose derived stem cells (hADSC)) and injected into a mold. Adapted with permission from ref (50). Copyright 2019 Wiley. (d) GelMa beads are annealable via UV cross-linking. Bottom panel shows interstitial spaces between the beads after annealing. Adapted with permission from ref (322). Copyright 2019 The Authors. (e) Extruded granular bioink, composed of norbornene-modified hyaluronic acid (NorHA) beads suspended in PBS. Scale bar is 500 μm. Adapted with permission from ref (72). Copyright 2019 Wiley. (f) Scheme of a strategy based on cell-mediated annealing of the beads after injection in vivo. Adapted with permission from ref (247). Copyright 2019 Wiley. (B) (a) 3D printing of hydrogel droplets into oil-lipid bath. (b) 3D rendering of the printed structure; (lower panel) expected and observed hexagonal arrangement of the cell-laden droplets. (C) (a) Ultrahigh throughput printing of core–shell alginate beads via in-air microfluidics. (b,c) A printed hollow tube with granular microstructure. (d) Multimaterial granular scaffold (dextran vs alginate). Scale bar are 1 cm (b) and 100 μm (c,d). Adapted with permission from ref (303). Copyright 2018 The Authors.

Finally, 2D networks consisting of hydrogel droplets connected by lipid bilayers have been proposed by Downs et al.^85^ as a part of a wider campaign of the Bayley group aimed at fabrication of large biomimetic constructs using lipid-stabilized droplets as building blocks (see next section 4.7.2 and Figure 18B). The authors demonstrated bottom-up assembly of temperature- and pH-responsive planar hydrogel microstructures (Figure 2B(m)) capable of shape-changing, with possible future applications, e.g., in soft-robotics. Yet, the work relied on the use of acrylamides as hydrogel precursors, which in general are known to be toxic for cells. Therefore, the potential application of such 2D droplet networks in tissue engineering would require switching to different hydrogel precursors.

### 4.7 Generation of Compartmentalized “3D” Structures

In the following we review the microfluidics-assisted strategies of fabrication of compartmentalized 3D biomaterials such as (i) injectable granular scaffolds, including granular bioinks, (ii) 3D printed droplet-based structures, (iii) 3D-printed or bundled microfibers and (iv) bulk porous materials. We provide an overview of such “3D” patterns for completeness and to provide the examples of the “bulk” counterparts of the previously discussed lower-dimensional topological microscaffolds. We limit the review to the structures with nontrivial internal topologies and to those assembled using microfluidics or composed of building blocks generated using microfluidics.

#### 4.7.1 Injectable Granular Scaffolds and Granular Bioinks

Hydrogel microbeads, fabricated using monodisperse microfluidic droplets as templates, can be close-packed into a 3D porous architecture (Figure 18A), wherein the surfaces of the beads may support cell attachment.^48,50,156,160,351,362^ Microgel-based porous structures have a great advantage over other types of porous scaffolds in that they are injectable and can thus be molded into any desired shape. After injection, the scaffold settles and self-stabilizes via annealing of the neighboring microbeads. Accordingly, microgel based scaffolds can serve as fillers for the damaged tissues. They have been already applied in regeneration of heart,^48^ nerve^50^ and skin tissue.^156^

Several annealing strategies have been proposed. Di Carlo, Segura, and co-workers^156^ exploited noncanonical amide linkage between transglutaminase peptide substrates attached to multiarmed poly(ethylene) glycol vinyl sulphone (PEG-VS) backbones forming the microgels (Figure 18A(a,b)). Later, a similar strategy was also used by the group to formulate annealable hyaluronic acid-based microgels,^363^ whereas the most recent work by the group focused on high-throughput fabrication of such microgels.^351^ An alternative approach to the bead annealing, based on electrostatic interactions, has been proposed by Hsu et al.,^50^ who used a mix of GelMa beads and chitosan oligomer-methacrylate (ChitoMa) beads carrying positive and negative surface charges, respectively (Figure 18A(c)). Yet another strategy has been explored by Sheikhi^160^ and Zoratto^322^ from Khademhosseini group, who used precross-linked GelMa microbeads which “sintered” at contact upon UV-assisted post-cross-linking (Figure 18A(d)).

In applications requiring delivery of large amounts of cells or biofabrication of granular high-cell-density constructs, the microbeads can be loaded with cells before forming the granular material. Such an approach was first demonstrated by Matsunga et al.^364^ from the Takeuchi group, who used microfluidics to formulate cell-laden collagen beads, also coated with other type of cells. Nearly centimeter-sized living structures have been formed via molding of such beads, resulting in surface cell densities of the order nearly 10^6^/cm^2^ (at sample thickness 100–200 μm), corresponding to a volumetric density similar to those found in vivo, ∼10^8^–10^9^ cells/cm^3^. The porosity of the close-packed structures allowed efficient nutrient delivery upon their immersion in the media and prevented necrosis in culture for at least 6 days.

Jamming of cell-encapsulating droplets has been more recently also used by Highley et al.^72^ in preparation of granular printable bioinks for applications in high-throughput 3D extrusion printing (Figure 18A(e)). In particular, encapsulation of cells inside the beads have been shown to protect the cells against shear stresses and to fully retain cell viability upon extrusion. Finally, Feng et al.^247^ used hybrid gelatin–hyaluronic acid microgels with embedded bone mesenchymal stem cells as injectable scaffolds for cartilage repair (Figure 18A(f)). In this case, annealing of the granular microbead scaffold was mediated solely by cell adhesion as the cells migrated to the surfaces of the beads (beads were precultured for several days prior to their close-packing). The study demonstrated significantly increased viability of the encapsulated cells upon injection as compared to nonencapsulated cells, especially at high injection rates (over 5 mL/h; needle diameter not specified). A similar strategy involving injectable stem cell-laden GelMa beads was also applied in bone regeneration.^365^

In the cases with low concentration of microbeads in the suspending bioink, the stability and/or injectability of the bioink depends mainly on the rheological properties of the suspending hydrogel matrix itself, whereas the beads serve as cell carriers,^269^ allowing coculture studies^115,231^ or as microenvironmental niches.^115,131^ For example, Agarwal et al.^115^ suspended core–shell microbeads laden with cancer cells inside an external collagen matrix with embedded endothelial cells to model tumor vascularization. These bihydrogel structures were assembled inside a perfusable microfluidic cell, which allowed direct observation of the development of the vasculature and further use of the system in drug testing. A similar gel-in-gel strategy was used to model a cancer microenvironment by Husman et al.^131^ The authors used PEG and heparin precursors as well as their mixtures to independently adjust hydrogel mechanical properties of the beads and the external matrix (see section 5.2.7 for more details on the vascularized tumor models).

#### 4.7.2 3D Printing of Hydrogel Droplets One-by-One

Droplet-based 3D bioprinting has recently emerged as a high-precision tool in 3D biofabrication. Villar et al. from the Bayley group demonstrated fabrication of 3D structures using adhesive (yet noncoalescing) microdroplets stabilized by phospholipids forming membrane-mimicking bilayers at droplet–droplet contacts (Figure 18B). More recently, Graham et al.^366^ demonstrated the possibility of printing of cell-laden hydrogel droplets, resulting in centimeter-sized constructs with controlled spatial cell patterning at the scale of 1–2 droplets (100–200 μm). The most recent development, the work by Zhou et al.,^88^ also from the Bayley group, constitutes an important step toward tissue engineering at the mesoscale. The authors 3D printed millimeter-sized brain organoids consisting of neural stem cells and astrocytes located in predefined regions of the printed construct. Long-term culture and in-depth biological analysis of the organoids allowed unique insights into human cerebral cortex development (see section 5.2.3).

Another droplet bioprinting technology, offering much higher throughputs at a cost of somehow lower precision, has been recently demonstrated by Visser et al.,^303^ who used high-frequency piezo-actuation to generate simple- or core–shell alginate droplets in air, directly followed by droplet collection and post-cross-linking at the substrate (Figure 18C). The technique allowed the fabrication of mesoscopic multimaterial granular 3D constructs such as a hollow tube.

#### 4.7.3 3D Bundles of Microfibers

**Figure 19:**
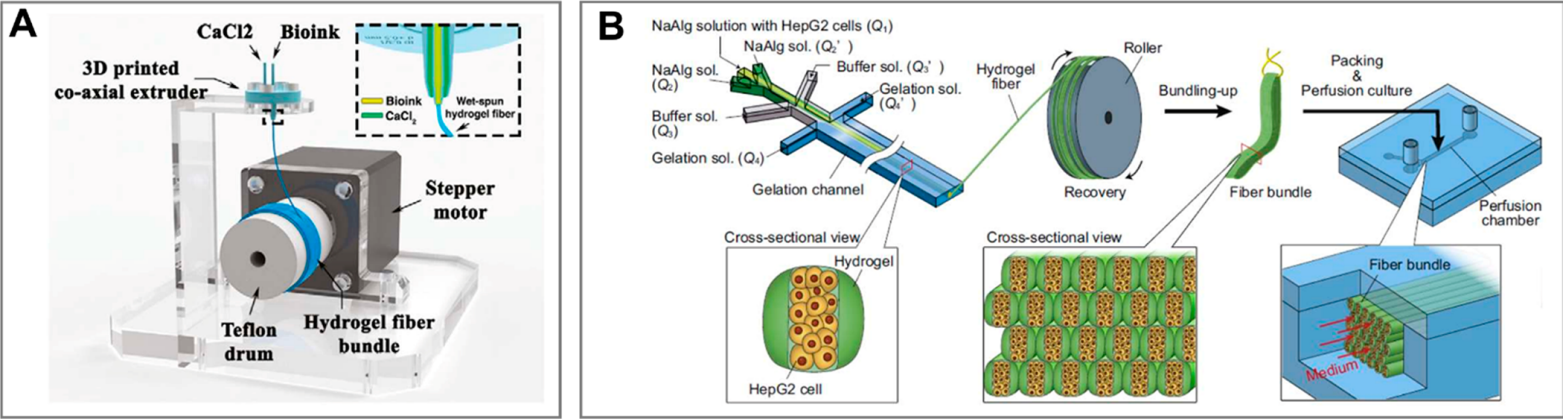
Microfluidics-assisted formulation of 3D bundles of microfibers. (A) Visualization of the experimental setup used for spinning of alginate–PEG–fibrinogen fibers. Adapted with permission from ref (46). Copyright 2021 The Authors. (B) Schematic representation of the microfluidic chip, the spinning system, and the on-chip 3D culture chamber for expansion of liver tissue. Adapted with permission from ref (74). Copyright 2018 Elsevier.

In analogy to 3D packing of microbeads or microdroplets, also microfibers have been tightly packed into 3D structures forming bundles or stacks (Figure 19). Stacking of multiple cell-laden microfibers allowed for generation of bulk biomaterials for applications in muscle regeneration^46^ or in development of a 3D liver model for drug toxicity screening.^74^ The packing of aligned microfibers can be achieved via their winding onto a rotating drum, with a tension applied to the fiber to avoid uncontrolled curling or knotting. Such a wound-up bundle can be easily removed from the drum and transferred into a Petri dish for further culture. Costantini et al. used microfluidics-assisted wet-spinning^46^ with encapsulated mesoangioblasts to generate few-millimeter-thick muscle-fiber bundles (Figure 19A). Formation of highly aligned myotubes was demonstrated after 15 days in culture. The structures were also implanted in vivo (for more details about this work, see section 5.2.5). In another demonstration, Yajima et al.^74^ fabricated a tightly packed bundle of hepatocyte-laden microfibers. The bundle was placed in a perfusable chamber for further long-term culture (Figure 19B), whereas the interstitial spaces between individual microfibers allowed efficient nutrient perfusion and warranted cell viability across the bundle.

For completeness, we also mention that 3D structures can also be fabricated via stacking of “2D” microsheets. Such approach has been demonstrated by Leng et al.,^59^ who spun alginate sheets into 3D stacks or 3D bundles (see Figure 2B(r,s)). However, to date, the utility of such a 2D–3D stacking technique in engineering of specific tissues has not been demonstrated.^59^

#### 4.7.4 3D Porous Materials Templated from Bulk Emulsions and Foams

Finally, we very briefly describe the most recent advancements in microfluidics-assisted fabrication of porous materials for tissue engineering. For a more comprehensive treatment of the field, we refer the interested reader to the recent review by Wang et al.^367^

**Figure 20:**
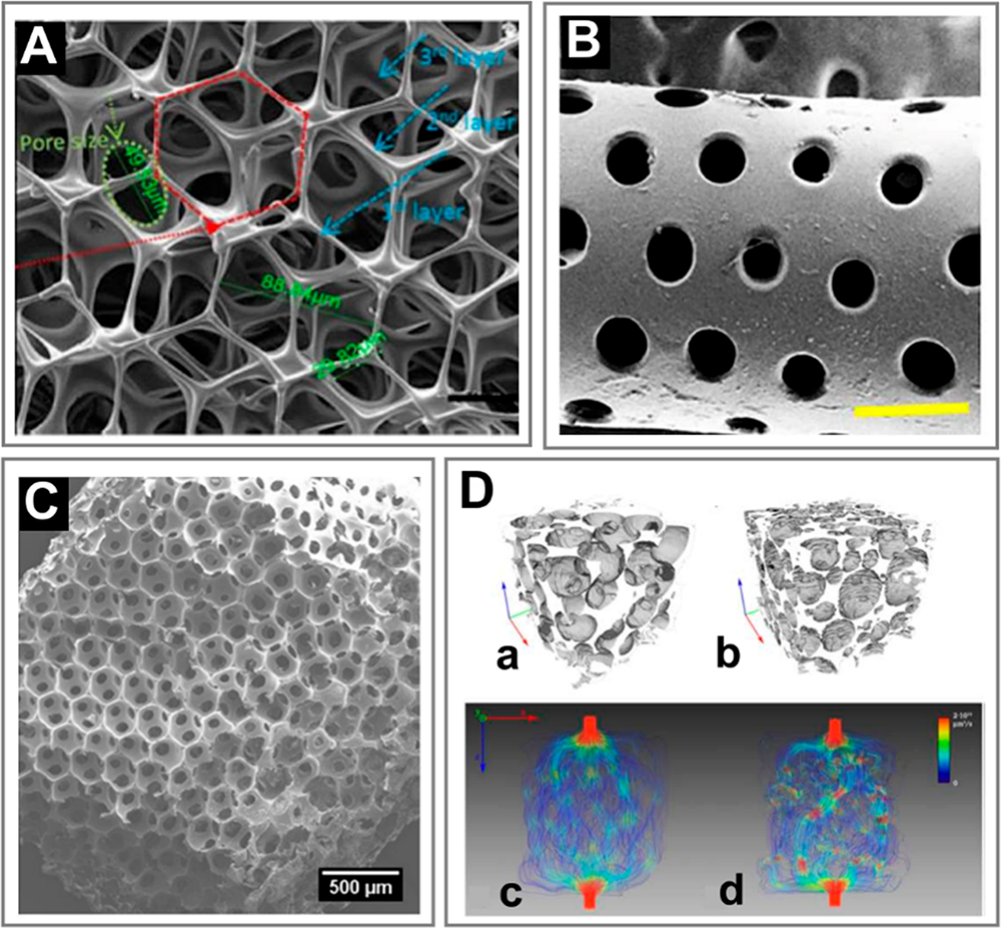
Microfluidics-assisted formulation of 3D bulk porous materials. (A) Gelatin bubble-based scaffold with pore and interconnection sizes around 100 and 50 μm, respectively. Adapted with permission from ref (279). Copyright 2019 Wiley. (B) Alginate–GelMa porous scaffold templated from microfluidic emulsion. Scale bar is 500 μm. Adapted with permission from ref (214). Copyright 2019 Elsevier. (C) Monodisperse GelMa foam generated with microfluidics. Adapted with permission from ref (256). Copyright 2019 American Chemical Society. (D) (a–d) Micro-CT scans and CFD flow simulation snapshots for (a,c) monodisperse and (b,d) polydisperse porous scaffolds Monodisperse structures develop visibly smoother and more homogeneous flows. Adapted with permission from ref (215). Copyright 2016 Elsevier.

The main advantage of the microfluidic emulsion- or foam-based scaffolds is the monodispersity of the pores^214,218,256,279,368,369^ as well as large pore and interconnection size^54,215^ (Figure 20A–C), facilitating infiltration of cells inside the scaffold^54^ as well as media inflow,^215^ warranting efficient nutrient supply to the seeded cells (Figure 20D).

The most recent advancements in the field include pore-size patterning across the structure achievable via changing the size of bubbles in real-time^54,370^ upon foam collection. Such control has been demonstrated via changing the gas flow rate^370^ as well as via adjusting the size of the microfluidic junction with the use of PDMS orifice with flexible walls, inflatable on demand.^54^ In particular, the latter strategy, proposed by Costantini et al.,^54^ allows varying of bubble size by nearly an order of magnitude without changing the bubble volume fraction (see Figure 29F). The authors combined the technique with 3D printing and demonstrated fabrication of a 3D bone-like structure with internal gradients in pore size.

Finally, we note that the internally patterned 3D tissue engineering scaffolds can be generated by using a combination of various microfluidic fabrication techniques. Recent examples include liposome-laden GelMa microbeads used in combination with 3D printed bioceramic scaffold for bone tissue regeneration^128^ or alginate cell-laden beads sandwiched between electrospun nanofiber mats, also applied in bone engineering^273^ (see also section 5.2.6).

### 4.8 Comparison of Microfluidics with Other Formulation Methods

As a summary of the microfluidic methods of formulation of microgels for tissue engineering, we provide a list of general advantages and disadvantages of microfluidics as compared to other formulation methods (see Table 2). Those other methods include (i) cell aggregation in microwells, (ii) layer-by-layer (LbL) assembly of multilayered spherical core–shell structures,^44^ (iii) formulation of spherical hydrogel structures exploiting superhydrophobic substrates,^44^ (iv) formulation based on micropatterned substrates (biochemical, magnetic, dielectrophoretic patterning; see ref (371) and references therein), (v) electrospraying and electrospinning,^372^ and (vi) 3D printing of mesostructures using adhesive hydrogel droplets.^88^ Shortly, the most important advantages of microfluidics (including microfluidic spinning) include the very high throughputs of their formulation and the variety of the available topologies (even at the length scale of 100 μm), all achievable with an excellent reproducibility (i.e., monodispersity). This particularly applies to the microparticles (“0D”) and microfibers (“1D”) which can be generated using mirofluidics in a fully automated one-step process. Generation of “2D” and “3D” structures requires additional manipulation of the pregenerated “0D” or “1D” building blocks, e.g., their stacking, bundling, weaving, etc., which is typically more time-consuming and less reproducible. Nevertheless, among available methods of generation of “2D” and “3D” constructs, microfluidics still offers very high precision and, in some cases, also the highest throughputs (e.g., the case of in-air microfluidics^303^). At the same time, microfluidics also allows for direct generation of unique “2D/3D” topologies such as longitudinally and/or transversally patterned, or pattern-coded bundles, sheets,^59^ or complex granular scaffolds,^303^ which are not always easily accessible by other methods. Among disadvantages, preparation of microfluidic devices is relatively complex (includes microfabrication of the chip, surface modification) while the experiments involve the use of rather costly equipment (e.g., the syringe pumps). Also, the fabrication of “0D” structures typically requires the use of external oil as the carrier phase (except for all-aqueous systems^217^ or in-air cross-linked droplets^303^), which must be washed out before culture and may possibly impact cell viability. Yet, we note that the most widespread “oils” in microfluidic biomedical applications, that is, fluorinated fluids such as Novec 7500 or FC40, are actually considered fully biocompatible.

**Table 2:** Advantages and Disadvantages of Microfluidic and Various Nonmicrofluidic Methods of Formulation of Topological Microtissues

method	complexity of the generated microtissue	size and monodispersity of the generated microtissue	throughput	complexity of the experimental setup	degree of automation of the formulation process	organic solvents (potential cytotoxicity)
microwells	“0D” structures (no compartments)	no size limit; monodispersity low	low	low; no special equipment required	low; manual one-step process (pipetting)	no
layer-by-layer (LbL) assembly	“0D” structures of core–shell topology (1 cellular compartment)	monodispersity and size depending on the process of core generation	mode rate	low to high depending on the process of core generation	moderate; involves a number of manual washing steps (same as the number of shells)	no
superhydrophobic surfaces	“0D” structures of core–shell topology (2 cellular compartments)	>1000 μm (limited by the pipetted volume); monodispersity high	low	low; no special equipment required	low; manual one-step or multistep process (when combined with LbL)	no
micropatterning based methods	“0D” or “2D” structures of Janus topology (2 cellular compartments)	>50 μm; monodispersity high	mode rate	moderate; requires microfabrication	moderate. automation limited; requires calibration/optimization of the setup	no
electrospray/electrospinning	complex “0D” and “1D” structures of core–shell or Janus topologies (multiple cellular compartments)	200–500 μm; monodispersity high	high	moderate; requires specialized equipment	high; automated process; requires calibration/optimization of the setup	no
3D printing of adhesive droplets	complex “2D” and “3D” structures of Janus topologies (multiple cellular compartments)	>1000 μm; monodispersity moderate	moderate to high	high; requires specialized equipment and microfabrication	high; automated process; requires calibration/optimization of the setup	yes/no
microfluidics/microfluidic spinning.	Complex “0D”, “1D”, “1.5D”, “2D”, and “3D” structures of core shell and Janus topologies (multiple cellular compartments)	100–500 μm; monodispersity high	high to very high	high; requires specialized equipment and microfabrication	high; automated process; requires calibration/optimization of the setup	yes/no

## 5 Applications of Topological Microgels in Tissue Engineering

Having reviewed the microfluidic methods of fabrication of cell-laden microgels, we now turn to a more detailed description of their applications in tissue engineering. We start with general considerations before turning to the tissue-specific examples.

### 5.1 General Considerations

First, we review the applications dictated by the dimensionality and topology of the microgels, which in many cases dictate the types of tissues that a given structure may actually support.

#### 5.1.1 Controlling Microgel Dimensionality and Topology as a Route to Biomimetics

**Figure 21:**
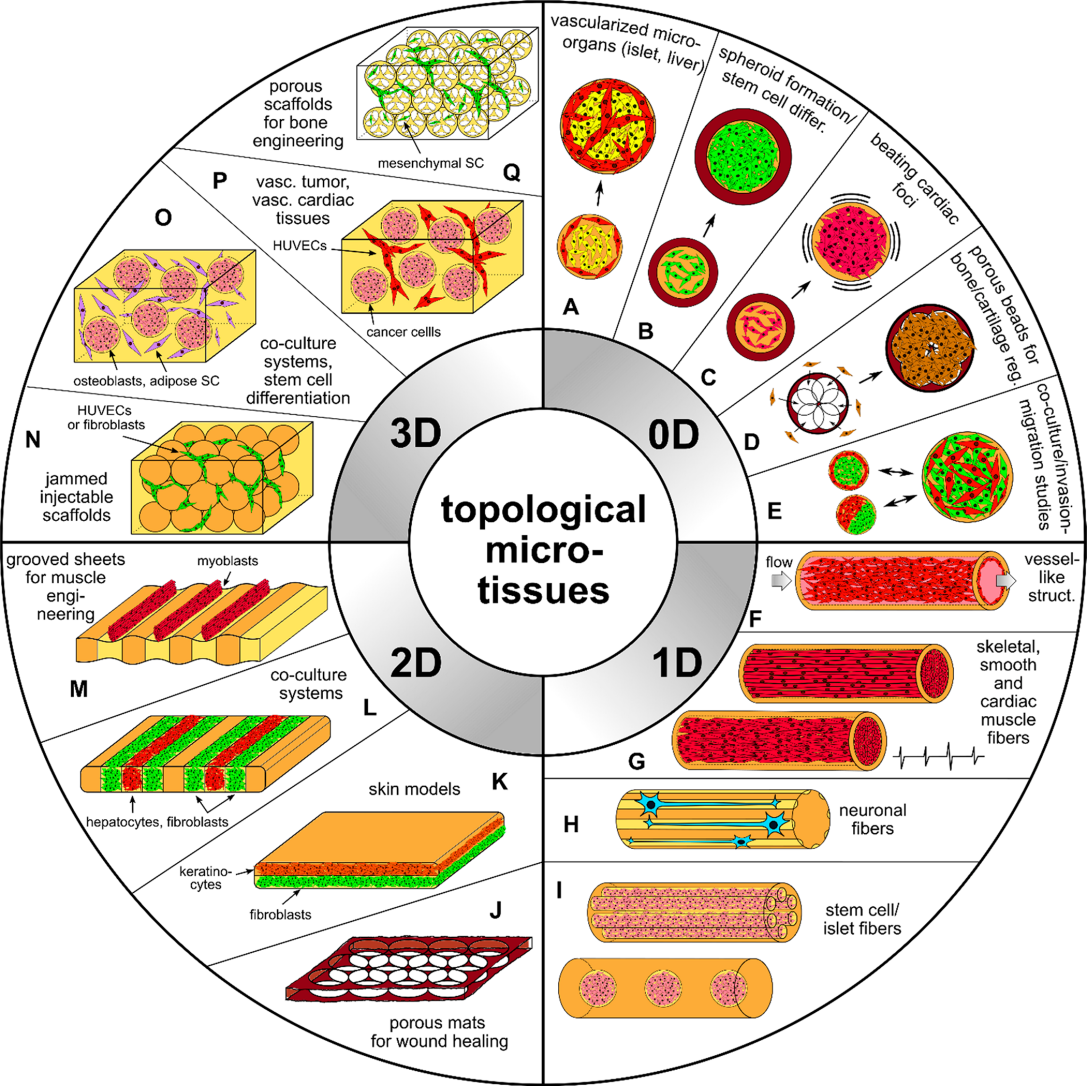
The variety of applications of compartmentalized microgels in microtissue engineering. Schematic representation of various microstructures achievable with microfluidics, classified in terms of their dimensionality.

The variety of structures available with microfluidics inspires the variety of applications (Figure 21). Starting with “0D” objects, the various types of core–shell structures may naturally serve as models for compact tissues or tissue parts (Figure 21A) such as pancreatic islets,^161,217^ hepatic tissue,^175^ and small tumors^75,115,300^ as a microenvironment for stem cell aggregation (Figure 21B) or differentiation into organoids of reproducible dimensions and tissue-specific functions, such as, e.g., beating cardiac foci^238^ (Figure 21C). In regenerative or therapeutic applications, the shells can be used to protect the encapsulated cells against patient’s immune response^132^ or against ice formation upon their cryopreservation.^121^ Multicore hollow particles, i.e., porous beads^64−66^ (Figure 21D) have been also used in bone engineering, e.g., as dental fillers.

On the other hand, the “0D” Janus structures have been applied to study (i) cell migration (Figure 21E),^76,176,373,374^ a mechanism of particular relevance in cancer invasion,^374^ or (ii) intercellular signaling.^76^ In particular, the Janus structures are beneficial in that they provide a well-defined flat interface between the different compartments which facilitates precise measurements of cell migration and allows control of the average cell–cell distance.^176^ Finally, cellular self-assembly including transitions between intermixed and core–shell or Janus microtissue morphologies have been observed using various initial morphologies (Figure 21E).^75,230^

The compartmentalized “1D” structures naturally reproduce the topology of tissues forming tubes, such as blood vessels^262^ (Figure 21F), or bundles, such as skeletal or cardiac muscle (Figure 21G),^73,277^ or otherwise consisting of elongated cells or cells forming very long protrusions such as neurons^82,274^ (Figure 21H). Fibers have also been used to culture cell fibers (analogues of cell spheroids) using stem cells^68,106^ or islet cells^45,51^ (Figure 21I). Stacking,^73^ bundling,^46,74^ or weaving^45^ of cell-laden fibers have been used to fabricate large tissue constructs such as skeletal muscle^46,73^ or hepatic tissue.^74^

The compartmentalized “2D” structures, such as porous mats^360^ (Figure 21J) or thin biomaterial sheets^70^ (Figure 21K), have been applied as dressings for wound healing or skin regeneration, respectively, whereas grooved (Figure 21L) or striped (Figure 21M) sheets find use in muscle,^212^ neuron,^274^ and liver^83^ tissue engineering.

Finally, the compartmentalized **“3D”** structures have been used to guide cell growth in the bulk of the material. In particular, in 3D structures built of close-packed and/or annealed microgel beads, the interstitial spaces between the beads form a system of interconnected cavities and as such represent a suitable scaffold for engineering of tissues such as vasculature,^115,303^ dermal,^156^ or neural tissue^50^ (Figure 21N). On the other hand, cell-encapsulating beads embedded in an external cell-laden hydrogel matrix also allow engineering of 3D coculture systems including bone^231^ and neural tissues^221^ (Figure 21O) as well as 3D vascularized pancreas^303^ or vascularized tumor models^115,131^ (Figure 21P).

Finally, close-packed microfluidic emulsions or foams have been extensively used as templates for fabrication of 3D porous materials, e.g., for bone engineering^54,370^ (Figure 21Q).

In the following sections, we highlight the applications of topological microgels and microtissues in (i) tissue modeling for basic tissue-biology research, (ii) tissue restoration and regeneration, and (iii) high-throughput drug testing.

#### 5.1.2 Tissue Modeling

Arrangement of the tissue-specific cells and auxiliary cells into separate compartments allows to control homotypic vs heterotypic cell–cell interactions and as such to mimic cellular interactions in actual tissues. In vivo, fibroblasts commonly serve as the auxiliary cells in various tissues as they secrete growth factors and cytokines beneficial for the tissue-specific cells. The endothelial cells may also play a similar role, as their presence positively impacts overall cell viability, functionality, and gene expression via paracrine signaling effect. The importance of those two cell types in organoid culture has been verified via microfluidic coencapsulation.^230,175,375^

In particular, in **liver microtissue** engineering (see section 5.2.1), various types of compartmentalized microgels have been used to demonstrate advantages of hepatocyte–fibroblast or hepatocyte–endothelial coculture over hepatocyte monoculture. The hydrogel microstructures utilized for this purpose include core–shell capsules,^175,246,265,309,364^ core–shell fibers,^74,112,355,356^ Janus fibers,^79^ and multi-Janus sheets.^83^ Both fibroblasts and endothelial cells (ECs) have been shown to improve viability^82,112,309^ and gene expression^74,83,355,356^ as well as albumin and urea synthesis^83,175,230,265,309,355,356,364^ in liver organoids.

**Pancreatic cells** (section 5.2.2) differentiated from human induced pluripotent stem cells have been encapsulated in core–shell fibers,^51^ core–shell beads,^68^ as well as in droplet-filled fibers.^68^ These encapsulation strategies showed remarkable success in islet engineering, demonstrating improved gene expression as well as glucagon and insulin secretion. In particular, expression of both glucagon and insulin-specific genes indicated presence of beta- and alpha-like cells in the cultured organoids,^68^ thus mimicking the actual cell heterogeneity in pancreatic islets.

In **neural microtissue** engineering (section 5.2.3), biomimetic wet-spun core–shell fibers coencapsulating neurons (in the core) with support of the so-called Schwann cells (in the shell) have been shown to enhance neuronal differentiation.^114^ Another approach has exploited neuronal growth factor laden GelMa microbeads as cell carriers embedded in extruded GelMa fibers, with PC12 cells and RSC96 Schwann cells supplied to the beads and the fibers, respectively. Such coculture was shown to enhance neurite outgrowth and elongation of PC12 cells.^221^

**Cardiac** cell aggregates (section 5.2.4) formulated using microfluidic encapsulation inside cardiac ECM-hydrogel microdroplets have been shown to develop cardiac-specific behavior such as pulsatile calcium release as well as expression of α-sarcomeric actinin and connexin-43, indicating the ability of adjacent cells to form electrical couplings.

Biomimetic approaches based on core–shell fibers have been exercised in engineering of tissues of native tubular morphology such as **skeletal muscle** (section 5.2.5) and **blood vessels** (section 5.2.7). In particular, the tailored “1D” microgel scaffolds were used to guide the development of highly aligned myotubes and allowed engineering implants for restoration of a functional muscle tissue as verified in vivo.^46,73^ Tubular scaffolds were also used to assemble perfusable blood vessels with liquid-tight vessel walls consisting of biomimetically arranged layers of endothelial and smooth-muscle cells.^262^

Further, microfluidic formulation of functionally graded porous materials^127^ has been demonstrated as a tool toward development of trabecular structures with a gradient in pore size and/or porosity, recapitulating structural complexity of **bone tissue** (section 5.2.6).

Finally, several microhydrogel-based systems have been proposed to model the complex **tumor** microenvironment (section 5.2.9), including (i) core–shell microcapsules with cancer cells in the core, with the shell playing the role of the basal membrane^374^ or serving as the host ECM of adjustable stiffness,^115,191,300^ (ii) microgel-in-gel structures with cancer cells in the microgel and endothelial cells in the outer matrix mimicking the host vasculature,^115,131^ and (iii) fibers with slits^206^ or hydrogel–media interfaces^374^ for studying cancer invasion. Also, the role of cancer stem cells in tumor malignancy has been investigated using the core–shell carriers.^188^

#### 5.1.3 Regenerative Medicine and Cell Therapies

In regenerative medicine, hydrogel droplet- or fiber-based scaffolds can be exploited as microbioreactors to grow macroscopic amounts of tissues. The advantage of using such microcarriers consists in limited risk of hypoxia. Indeed, the small dimensions of the scaffold and associated high surface-to-volume ratio warrant undisturbed access to nutrients and oxygen as cells start to proliferate.^51^

In general, applications of cell-laden microgels in regenerative medicine are particularly challenging due to (i) possibility of immune rejection by the host, (ii) low cell viability after implantation due to the lack of vasculature, as well as (iii) degradability issues associated with the use of particular types of hydrogels. The available in vivo demonstrations are yet scarce but in some cases yield quite spectacular results. Regenerative approaches have been particularly successful in engineering of pancreatic,^51^ muscle,^46,285^ neuron,^50^ and cardiac tissue,^47,48^ as well as in wound healing^156^ and bone regeneration.^231^

In the case of **stem cells** (section 5.2.8), microencapsulation has been also shown to enhance pluripotency,^95,107,111^ a feature desirable in stem cell therapies. Alternatively, the core–shell structures have been used to provide controlled conditions for differentiation of stem cells into organoids,^47,107^ resulting in microtissues developing organ-specific functions.

Finally, we note that the mass production of microtissues for regenerative medicine require not only reproducibility but also sufficiently high throughputs.^50,364^ Various routes toward integration of multiple (tens hundreds, or even thousands) of droplet generators have been demonstrated,^296,297,376−379^ resulting in generation of hydrogel droplets at frequencies in the range 10^3^–10^6^ Hz, which correspond up to tens of kilograms of microgels per day. Other approaches focused on upscaling the throughput of a single generator via exploiting an inviscid gas (rather than a viscous liquid) as the external phase. The strategy has been recently applied to cross-link not only simple droplets but also internally structured ones, such as core–shell or Janus droplets, “on the fly” upon transit (in air) between the droplet generator and a collection chamber^199,303^ at frequencies of the order 10^5^ Hz. Overall, the recent advancements in high-throughput microgel fabrication seem very promising in view of regenerative medicine applications in which millions of microtissues could be used for biofabrication of tissues or organs at the human scale.^303^

#### 5.1.4 Drug Testing

Topological microtissues have been successfully used in screening of various therapeutics. The hydrogel bead-based drug screening assays, as compared to conventional methods, consume much less reagents, are highly repeatable, and can provide shorter response times and higher levels of control over cell and drug concentrations. Microfluidic-based topological microtissues may help researchers in evaluating new drugs before (or instead of) turning to in vivo animal models. In the future, such miniaturized 3D in vitro methods could significantly limit or even eliminate the use of animals in drug testing.

It is known that cancer cells grown in physiological 3D microenvironments develop different (typically higher) resistance to drugs than those grown in 2D monolayers.^26−28,380^ Therefore, the development of anticancer drugs requires the use of possibly realistic 3D in vitro tumor microenvironment to accurately capture drug efficacy. Agarwal et al.^115^ reported a 3D vascularized microtumors based on cancer cell-laden hydrogel core–shell beads embedded in the external endothelial cell-laden hydrogel matrix, which developed up to 100-fold higher resistance to doxorubicin hydrochloride (a commonly used chemotherapy drug) as compared to 2D cultured cancer cells and even 5-fold higher as compared to the avascular microtumors. The latter observation, in particular, clearly demonstrated the relevance of vascularized 3D models in developing the effective therapeutic strategies to fight cancer.

Hydrogel microbeads were also used by Jiang et al.^20^ to develop an automated personalized drug screening platform based on patient-derived cancer cells encapsulated in the beads and individually printed into microwells. Encapsulation accelerated formation of microtumors, allowing performance of multiple drug screens within a one-week period. In particular, the authors demonstrated that the technology could be used to capture patient-specific responses to different anticancer drugs. The study showcased the possibility of performing the automated personalized drug screens in a clinical setting.

Considering drug toxicity screening, Kukla et al.^229^ from Khetani group used human hepatocyte-laden collagen microbeads as model liver microtissues with intermixed- or coated 3T3-J2 fibroblasts to study drug-induced hepatotoxicity against common drugs such as rifampin, omeprazole, rosiglitazone, or troglitazone. In particular, significantly different responses as compared to those in 2D monoculture were found, thus demonstrating a significant role of paracrine effects and 3D microenvironment on the measured drug toxicity. A similar approach was developed by Li et al.,^266^ where primary hepatocyte/fibroblast microtissues were used to evaluate cytochrome P450 enzymes activity in response to omeprazole, dexamethasone, rifampin, and phenobarbital. Here, however, instead of single cell suspension the authors used a mixture of hepatocyte islands (so-called hepatocyte pucks) prepared by seeding liver cells in collagen-coated microwells, and J2-3T3 fibroblasts to generate hepatocyte/fibroblasts-laden PEGDA microbeads. Formation of hepatocyte or hepatocyte/J2-3T3 aggregates prior to encapsulation increased hepatic functions of the liver microtissues including higher levels of albumin production. Importantly, primary hepatocytes were used in the study. This showed that the liver microtissues could be used to assess liver cytotoxicity in individuals with varying risk factors such as age, gender, genetics, or underlying diseases.

Whereas the available demonstrations of microtissue-based drug screening focus on development of new anticancer therapeutics, the efficacy of other types of therapeutic agents such as anti-inflammatory or antineurodegenerative drugs has not yet been tested using microtissues. A possible reason is the lack of nontumor-derived neuronal cell lines, general complex nature of the neuronal tissue, as well as the necessity of evaluating the cognitive functions to asses the efficiency of drugs. Therefore, in the case of neural tissue, in vivo models still seem to be the only possible solution.

### 5.2 Tissue- and Cell-Type Specific Applications

Finally, we turn to a detailed review of the most recent developments in microfluidic formulation of microtissues in the tissue-specific context. In particular, we focus one-by-one on the most widely explored tissues including liver, pancreas, neurons, cardiac tissue, skeletal muscle, bone, and vascular tissue. We also discuss the recent progress in encapsulation of stem cells and cancer cells as the emerging strategies in regenerative and personalized medicine.

#### 5.2.1 Liver

Liver is the largest gland in the body, responsible for a wide range of biological functions including bile production, detoxification, as well as carbohydrate, protein, and lipid metabolism, synthesis of various hormones, and homeostasis. Human liver is divided into a large right lobe and a smaller left lobe, which are each subdivided into lobules, hexagonal units comprising of hepatocytes, and a capillary network built of liver epithelial cells, which together constitute approximately 80% of liver mass. Drug-induced hepatic injury, acute or chronic, is the leading cause of removal of drugs from the market. High sensitivity of liver to drug toxicity is related to its large metabolic capacity and the fact that many therapeutics eventually accumulate in the liver. This raises the need for the development of high throughput strategies dedicated to efficient and fast evaluation of drug toxicity as well as development of new liver tissue restoration therapies. Microfluidic-based hepatic tissue engineering has experienced tremendous progress in recent years.^373^ Numerous 3D models of liver microtissues ranging from simple monoculture “0D” structures^189,265^ to coculture systems of various topology and cellular composition^67,74,79,82,83,89,175,201,230,246,271,309,355,356,364^ have been developed to study the effects of drugs on cells and facilitate new approaches to liver cell transplantation.

**Figure 22:**
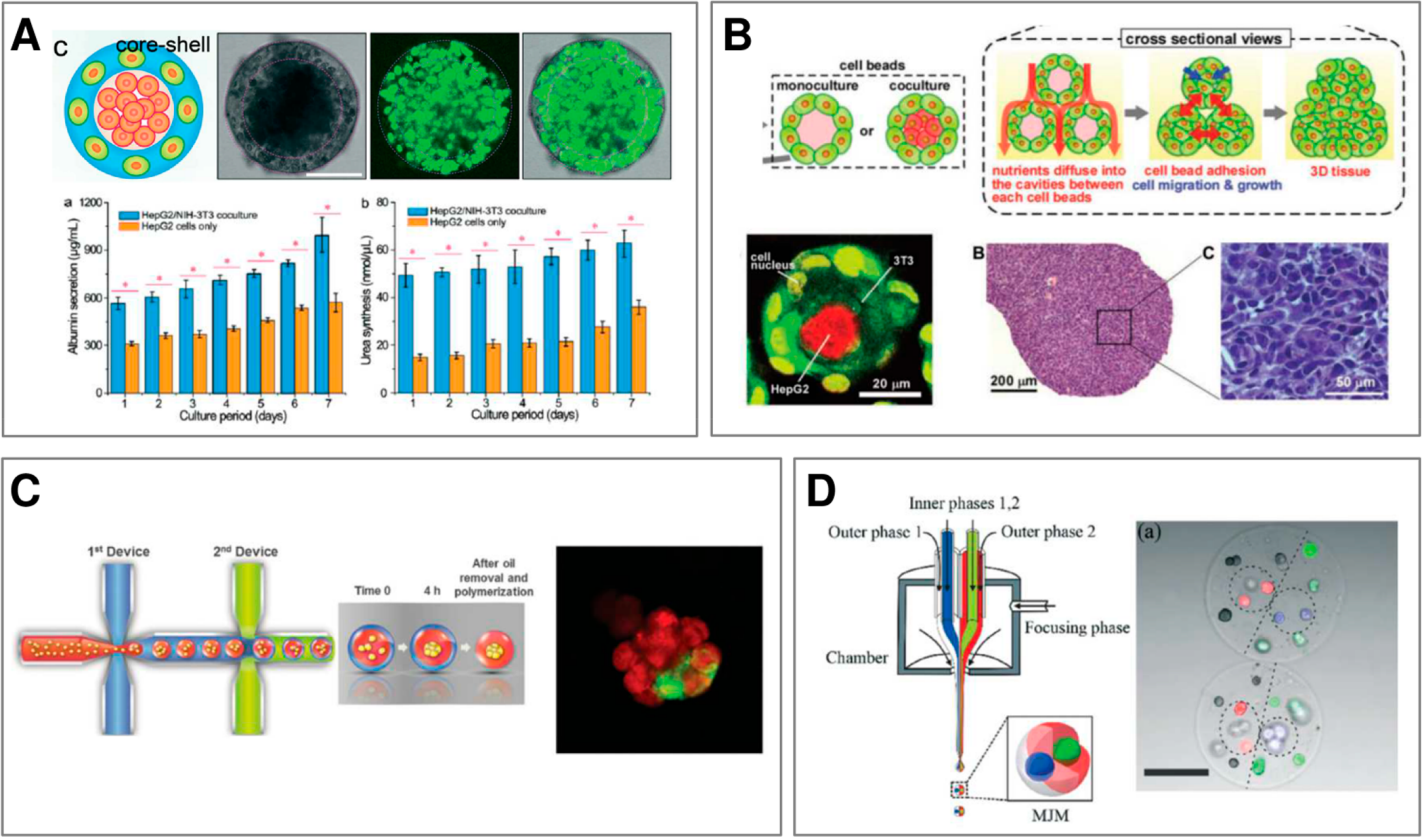
Microfluidics-assisted liver microtissues. (A) Spatial assembly of hepatocytes in the core and fibroblasts in the shell of the 3D core–shell capsules. Green color indicates viable cells (stained with calcein AM/EthD-1 staining kit). The scale bar is 100 μm (top panel). Analysis of the liver-specific functions of the hepatocyte/fibroblast coculture and hepatocyte culture including albumin secretion and urea synthesis measured after 7 days of culture (bottom panel). Adapted with permission from ref (175). Copyright 2016 The Royal Society of Chemistry. (B) Concept of bead-based tissue engineering using monodisperse cell-coated beads encapsulating another cell type molded into a 3D tissue architecture and illustration of the properties of the generated macrotissues (top panel). Fluorescent confocal image of the cocultured cell bead, 17 h after seeding the NIH 3T3 cells over the collagen gel beads encapsulating HepG2 cells (bottom left) and a microscopic view of the liver-like tissue section after reconstruction for 24 h (bottom right). Adapted with permission from ref (364). Copyright 2011 Wiley-VCH. (C) Schematic diagram of the process of generation of microencapsulated hepatocyte spheroids using double-emulsion droplets and a microscopic image of cell organization in the coculture spheroids incorporating endothelial progenitor cells (green) and hepatocytes (red). Adapted with permission from ref (230). Copyright 2016 Wiley-VCH. (D) Schematic of the experimental setup for production of multicompartment Janus microcapsules by the multiplex coaxial flow focusing system (right). Fusion of bright field and fluorescence images of multicompartment Janus microcapsules that encapsulate four types of cells in the designated compartments. HL7702 cells (black), LX2 cells (red), HUVEC (green), and HepG2 cells (blue). Scale bar 100 μm. Adapted with permission from ref (89). Copyright 2017 The Royal Society of Chemistry.

Cell-laden compartmentalized capsules of core–shell topology constitute the most common and well-characterized approach toward liver microtissue engineering.^175,189,230,246,265,309^ Core–shell architectures with hepatocytes residing in the aqueous- or soft-hydrogel core, surrounded by a rigid-hydrogel shell, allow for spontaneous aggregation of cells into hepatic cell spheroids.^175,189,230,246,265,309^ Core–shell microgels may also serve as convenient coculture scaffolds, facilitating incorporation of fibroblasts^175,246,309^ or endothelial cells^230^ in either core^230^ or shell,^175^ aiming to improve biological functions of the coencapsulated hepatocytes. Chen and co-workers^175^ developed a flow-focusing microfluidic device that allowed for generation of water–water–oil (w/w/o) double-emulsion droplets, which were used as templates to successfully formulate the 3D core–shell scaffolds with hepatocytes in the liquid core surrounded by fibroblasts in the alginate hydrogel shell. HepG2 cells and NIH-3T3 fibroblasts self-assembled within the capsules into core–shell microtissues with both homotypic as well as heterotypic cell–cell interactions, which promoted liver-specific functions (Figure 22A). The authors demonstrated that fibroblasts/hepatocytes coculture in core–shell microcapsules significantly increased albumin secretion and urea synthesis as compared to monoculture capsules with HepG2 cells alone. The beneficial effect of fibroblasts/hepatocytes coculture in microgels was later confirmed in other studies,^246,265,309^ showing that introduction of fibroblasts into liver organoids can lead not only to increased secretion of albumin and urea synthesis^265,309^ but also to higher expression levels of liver-specific metabolic genes such as tyrosine aminotransferase (TAT), glucose 6-phosphotase (G6 Pase), or cytochrome enzymes such as cytochrome P450 1A2 (CYP1A2) or cytochrome P450 3A4 (CYP3A4).^265^

Various coculture strategies have been developed to investigate the interplay between hepatic cells and fibroblasts. For example, Liu and co-workers^309^ used a coaxial flow-focusing microfluidic system to encapsulate intermixed HepG2 cells and 3T3 fibroblasts in the core of core–shell alginate microcapsules. In another study, Siltanen and colleagues^265^ used polymer microcapsules with liquid core and solid shell composed of cross-linkable PEG4MAL and inert PEG, which were used to encapsulate primary rat hepatocytes. To enhance hepatocyte–fibroblast heterotypic cell interactions, cell-laden hydrogel beads with hepatic cell spheroids were seeded directly onto 3T3-J2 fibroblast feeder monolayer. Last but not least, fibroblast–hepatocytes interactions can be enforced by coating of hepatic cell-laden hydrogel beads with fibroblasts. This approach was first studied by Sakai et al.,^246^ who developed Gelatin-Ph microcapsules with HepG2 cells encapsulated in the core of gelatin core–shell beads, which were subsequently coated with L929 fibroblasts. Cell-laden microparticles with hollow cores were produced using two types of gelatin with different physicochemical properties: unmodified gelatin which can be cross-linked by thermal gelation and gelatin-Ph, a gelatin derivative obtained by incorporating phenolic hydroxyl (Ph) cross-linkable via conventional thermal gelation and/or peroxidase-catalyzed cross-linking reaction, where the latter method stabilizes gelatin against melting at 37 °C. The production process consisted of generation of unmodified gelatin microparticles of less than 200 μm using microfluidic flow-focusing junction with liquid paraffin as the external immiscible phase. In the second step, the microparticles were coated with gelatin-Ph via re-encapsulation, followed by HRP-catalyzed cross-linking of the shell. Incubation at 37 °C led to melting of the gelatin cores containing HepG2 cells and facilitated aggregation.

Matsunga and co-workers^364^ demonstrated that hydrogel beads encapsulating HepG2 cells and coated with NIH-3T3 fibroblasts can be further used to build macroscopic 3D structures. The authors developed a bottom-up approach toward rapid construction of millimeter-thick macroscopic liver tissue (Figure 22B). Fibroblasts not only enhanced albumin secretion from HepG2 cells but also served as a “binder” connecting the neighboring beads and allowing for formation of more complex architectures. The authors suggested also that by incorporating endothelial cells into the macroscopic structure, for example by coating cell-laden beads with HUVECs, one could generate vascularized-liver like tissues.

Despite highly supportive and well-documented beneficial effect of fibroblasts on hepatocytes, liver models based solely on hepatocyte/fibroblast coculture do not fully mimic the biological complexity of liver tissue. In vivo human liver is composed of parenchymal cells (hepatocytes) and nonparenchymal cells such as Kupffer cells, sinusoidal endothelial cells, and hepatic stellate cells that perform liver functions in synergy with hepatocytes. To address this issue, Chan et al.^230^ used a double-emulsion approach to encapsulate primary rat hepatocytes and endothelial progenitor cells (EPC) in alginate microbeads enriched with collagen. EPCs were used to mimic native liver endothelial cells and emulate the in vivo situation, where these two types of cells form a continuous lining. The hepatic spheroids were generated via encapsulation in the liquid (hydrogel precursor) core of double-emulsion droplets (Figure 22C). After 4 h of aggregation, the droplets were transferred into calcium bath in which the spheroid-enclosing collagen–alginate cores cross-linked and sedimented, while oil shells creamed on top of the solution and evaporated after transient contact with air (highly volatile Novec 7100 was used as the oil phase). Analysis of daily albumin release and urea secretion as well as cytochrome 450 enzymes activity showed that EPCs significantly increased hepatocytes functions and the optimal coculture ratio of 5:1 (EPCs: hepatocytes) was identified.

Multicompartment cell-laden Janus-like structures, allowing for incorporation of larger number cell types in various compartments with well-defined physicochemical properties, were also used to mimic the liver microenvironment.^89,201,271^ Wu and co-workers^89^ developed a multiplex coaxial flow focusing system for fabrication of multicompartment Janus microcapsules (MJMs). MJMs were generated in the axisymmetric jetting mode with three parallel coaxial needles. Depending on the number of coaxial needles, different number of cores could be integrated into a single MJM. The authors encapsulated four different cell lines: LX2 human hepatic stellate cells and HepG2 cells in two different cores surrounded by HL7702 normal human liver cells and HUVEC cells in the two outer compartments (Figure 22D). Most of the cells moderately proliferated and maintained viability within the alginate microgels. Despite remarkable potential, the system has not been further optimized since (e.g., via incorporation of ECM) toward generation of more biomimetic liver microtissues. In other study, Wu et al.^271^ developed alginate multicompartment Janus microparticles with 2, 6, 10, or even 20 separate compartments. The capsules were loaded with HUVEC and HepG2 cells, yet the strategy allowed in principle construction of particles of much higher cellular heterogeneity. Finally, Wang and co-workers^201^ suggested that hepatocyte-laden alginate Janus capsules could be arranged into more complex linear structures via generation of beads-on-string, also called “Buddha” fibers, which potentially could allow microtissue identification based on their consecutive arrangement along the fiber.

Even though hydrogel microcapsules constitute the largest group of microfluidics-based liver microtissues, several works also exploited the use of microfibers. The fibers, typically generated at higher mass throughputs as compared to capsules (due to higher flow rates in the jetting as compared to the dripping regime), seem to be particularly attractive in the context of liver tissue restoration. The strategies include core–shell^74,82,355,356^ and Janus microfibers,^67,79^ including those dedicated to coculture^67,79,82,355,356^ or/and applicable in generation of more complex liver-like structures aiming to mimic liver cords, plates, or even the whole lobule.^74^

A series of works have focused specifically on mimicking the hepatic lobule, a basic building block of the liver tissue, which comprises a portal triad, central vein, and columns of hepatocytes arranged in linear cords between a capillary network extending from the portal region to the central vein. The architecture of the cords was successfully recapitulated by Jia et al.,^356^ who employed microfluidic technology to generate alginate-based core–shell fibers with C3A human hepatocytes in the core and EA.hy926 human endothelial cells in the shell. The authors performed comprehensive analysis of morphology and functions of the generated structures showing that hepatic cord–biomimetic liver microstructures develop more physiological tissue morphology, as well as exhibit higher expression levels of drug-metabolizing enzymes and albumin and ammonia metabolism as compared to cell grown in conventional 2D conditions. The generated microtissues supported hepatocyte polarity and promoted expression of genes activating the liver bile secretion pathway.

**Figure 23:**
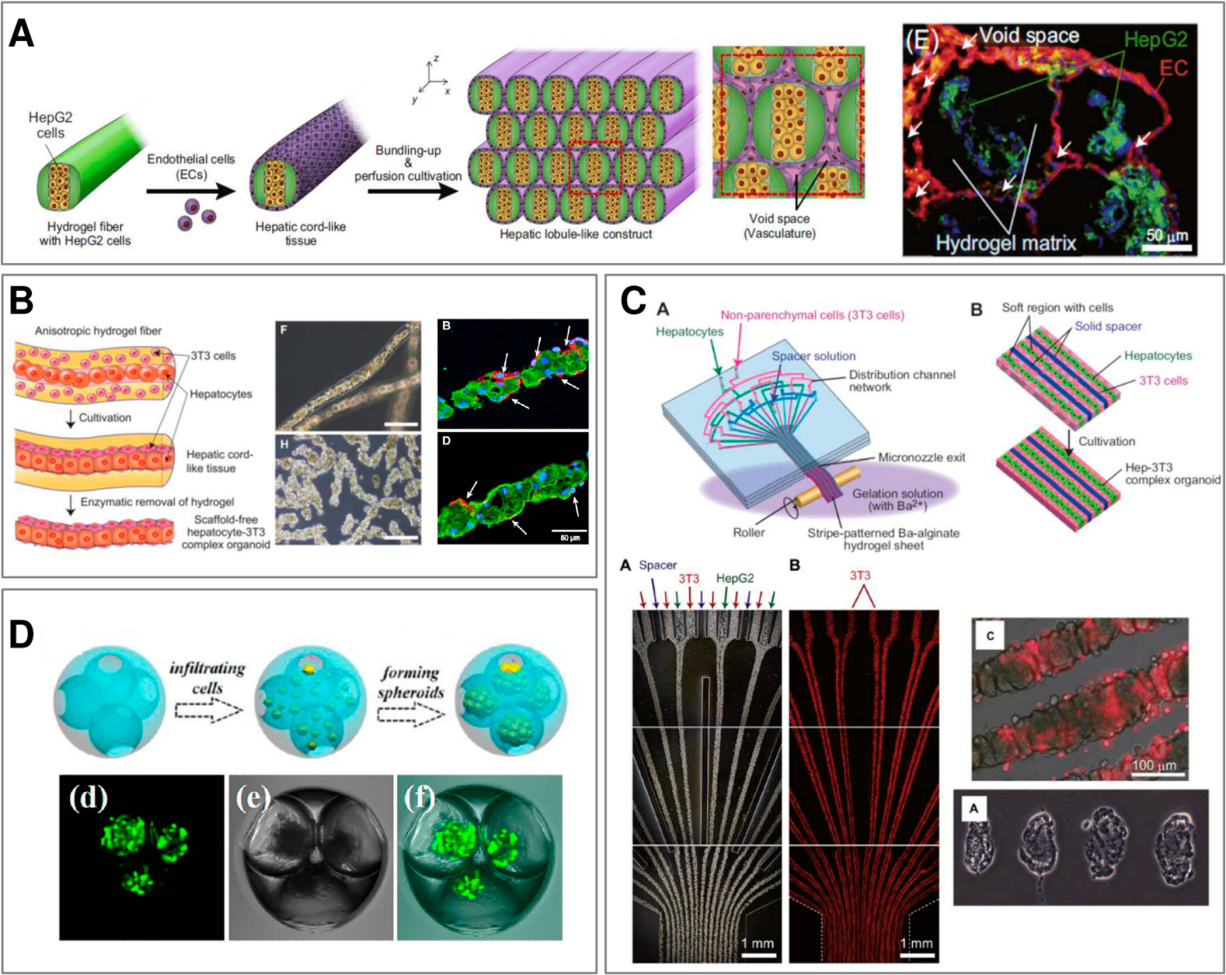
Microfluidics-assisted liver microtissues (continued). (A) Illustration of the fabrication process of the vascular network-like structures using fibers coated with endothelial cells (ECs) and a microscopic image of a section of the fiber bundle. HepG2 cells were stained green using antialbumin antibody, and cell nuclei were stained blue with DAPI. White arrows indicate the void spaces between the fibers. Adapted with permission from ref (74). Copyright 2018 The Society for Biotechnology Japan. (B) The formation process of hepatocyte–3T3 complex micro-organoids in the microfiber (left panel) and bright field images illustrating this process (middle panel). Confocal images of released liver organoids (right panel). The hepatocytes and Swiss 3T3 cells were immunostained with CK18 (green) and vimentin (red) antibodies. Arrows indicate 3T3 cells. Adapted with permission from ref (355). Copyright 2012 Elsevier Ltd. (C) Schematic showing the preparation procedure of the stripe-patterned heterogeneous hydrogel sheet and formation of heterotypic micro-organoids in the soft/solid hydrogel sheet (top panel). Flow behaviors of two types of cells (HepG2 and 3T3) inside the microchannel; bright field and fluorescence image (bottom left). Coculture of HepG2 and Swiss 3T3 cells in the heterogeneous hydrogel sheets: top view and cross-section (bottom right). Adapted with permission from ref (83). Copyright 2013 The Society for Biotechnology Japan. (D) Schematic diagram (top panel) and microscopic images (bottom panel) of the fabrication process of the cell microcarrier and formation of hepatic spheroid aggregates. Adapted with permission from ref (64). Copyright 2015 American Chemical Society.

An interesting approach to functional liver cords or even more complex hepatic-lobule-like constructs was also presented by Yajima et al.^74^ Sandwich-type barium alginate (BaAlg) hydrogel microfibers with HepG2 cells encapsulated in the core were prepared using a microfluidic laminar coflow system. The fibers were bundled up by using a roller and packed in a perfusion chamber. To structurally mimic the 3D hepatic lobules and the sinusoidal structure of the liver, the HepG2 cell-laden fibers were precoated with endothelial cells. The authors showed that after 5 days of culture, microfibers formed an integrated multicellular 3D structure highly resembling liver topology and functions in vivo (Figure 23A). The strategy to generate sandwich-type anisotropic alginate hydrogel fibers mimicking liver tissue was actually initially developed by Yajima et al. in their previous study,^355^ in which 3T3 fibroblasts were used as the coculture cells. Hepatocyte/fibroblast cell aggregates were recovered from the fibers by enzymatic digestion and analyzed with respect to their morphology as well as to cytochrome 450 enzyme gene expression profile, albumin secretion, and urea synthesis (Figure 23B).

Structures with multiple parallel cord-like units were also presented by Kobayashi and co-workers.^83^ A microfluidic device combining multiple micronozzles generating parallel jets was used to fabricate stripe-patterned heterogeneous hydrogel sheets composed of multiple parallel stripe-like regions with varying physical stiffness. The HepG2 cells and 3T3 fibroblasts were encapsulated in the soft stripes composed of PGA and alginate in low concentration separated by stiff-hydrogel “spacer” stripes. Each cell-laden stripe consisted of parallel subregions containing separately hepatocytes and fibroblasts, thus forming a cord-like structure (Figure 23C). The structures were analyzed for albumin secretion and cytochrome 450 activity. The authors suggested that the presented heterogeneous hydrogel sheets could be used in the future to generate relatively large, but precisely controlled, three-dimensional microenvironments for high-density coculture of multiple types of cells, including fabrication of liver microtissues of more complex cellular composition.

The remaining group of topological microstructures dedicated to hepatic cell culture are porous beads, which, depending on the pore size, allow for either infiltration of dispersed hepatocytes into the bulk of the structure (materials with midsize pores, 50–100 μm in diameter)^66,214^ or for formation of multicellular spheroids inside the pores (materials with large pores, 200–500 μm in diameter).^64^ An interesting example of the latter strategy is the work by Wang et al.^64^ The authors generated microcarriers with two, three, or six pores. Aqueous core droplets were generated at the tip of the inner capillary and then encapsulated in a drop of ethoxylated trimethylolpropane triacrylate (ETPTA), resulting in double-emulsion drops with multiple inner cores, which served as templates for porous beads. HepG2 cells seeded onto such porous beads self-assembled into spheroids inside each pore of the microcarrier, resulting in capsules with hepatic cell aggregates of well-defined size (Figure 23D).

Altogether, the recently developed microfluidic platforms for in vitro 3D hepatocyte culture allow for generation of liver microtissues of various topologies and cellular composition that could contribute to the development of bioartificial liver for drug metabolism and toxicity studies as well as liver pathophysiology research. There is a need for new strategies of optimization of the liver microenvironment via selecting the appropriate cellular composition of the scaffolds that would enhance cell–cell interactions and cellular signals and thus contribute to generation of liver microtissues of higher biological relevance. Last but not least, current models are mostly based on the use of cancer-derived or immortalized cell lines, which both have reduced functions as compared to primary liver cells and may not fully reflect the normal physiological responses. Therefore, future studies should preferably involve the use primary hepatic cells or IPSCs-derived hepatocytes instead of HepG2 cells.

#### 5.2.2 Pancreas

Pancreas is an organ of the endocrine system responsible for hormone homeostasis. The endocrine cells such as beta and alpha cells located in the pancreas form compact aggregates, so-called islets of Langerhans or “pancreatic islets”. Although the mass of the islets represents only 2% of the whole pancreas, they are responsible for secreting the pancreas-specific hormones, including insulin, glucagon, and the pancreatic polypeptide and serve to maintain blood glucose homeostasis.^381^ Type 1 diabetes mellitus (T1DM) is a common and highly morbid disease caused by the autoimmune destruction of beta cells within the pancreas, resulting in dysregulation of blood glucose levels. A surgical treatment for type-1 diabetes has been proposed via islet transplantation, which, despite great promise, still has formidable drawbacks such as (i) the difficulties associated with cell viability upon islet isolation, (ii) the necessity of lifelong immunosuppressive therapy after transplantation,^382^ and (iii) shortage of donors. As a solution to these problems, new strategies for artificial islet generation based on microencapsulation of beta cells have been developed,^161,190,217^ including iPSC-derived beta cell encapsulation.^51,383^ Artificial islets are simplified versions of the actual pancreatic islets, capable of performing basic endocrine functions such as insulin production under hyperglycemia. Artificial islets are created in vitro via aggregation and self-organization of beta cells derived from stem cells into a 3D microtissue.

Creating high-fidelity islet organoid models in a reproducible and high-throughput manner remains challenging, but there has been a considerable progress in the field during the last 5–10 years, with unique opportunities provided by the development of microfluidics. Beta cell encapsulation has been demonstrated using core–shell hydrogel microspheres,^161,190,217,383^ core–shell hydrogel fibers,^45,51^ as well as droplet-laden fibers,^68^ in addition to simple hydrogel beads^384,385^ and fibers.^386^ In particular, the core–shell microcarriers provide a unique functionality via combining immune protection (via the solid shell) with tunable internal environment (liquid or semiliquid soft-hydrogel core).

**Figure 24:**
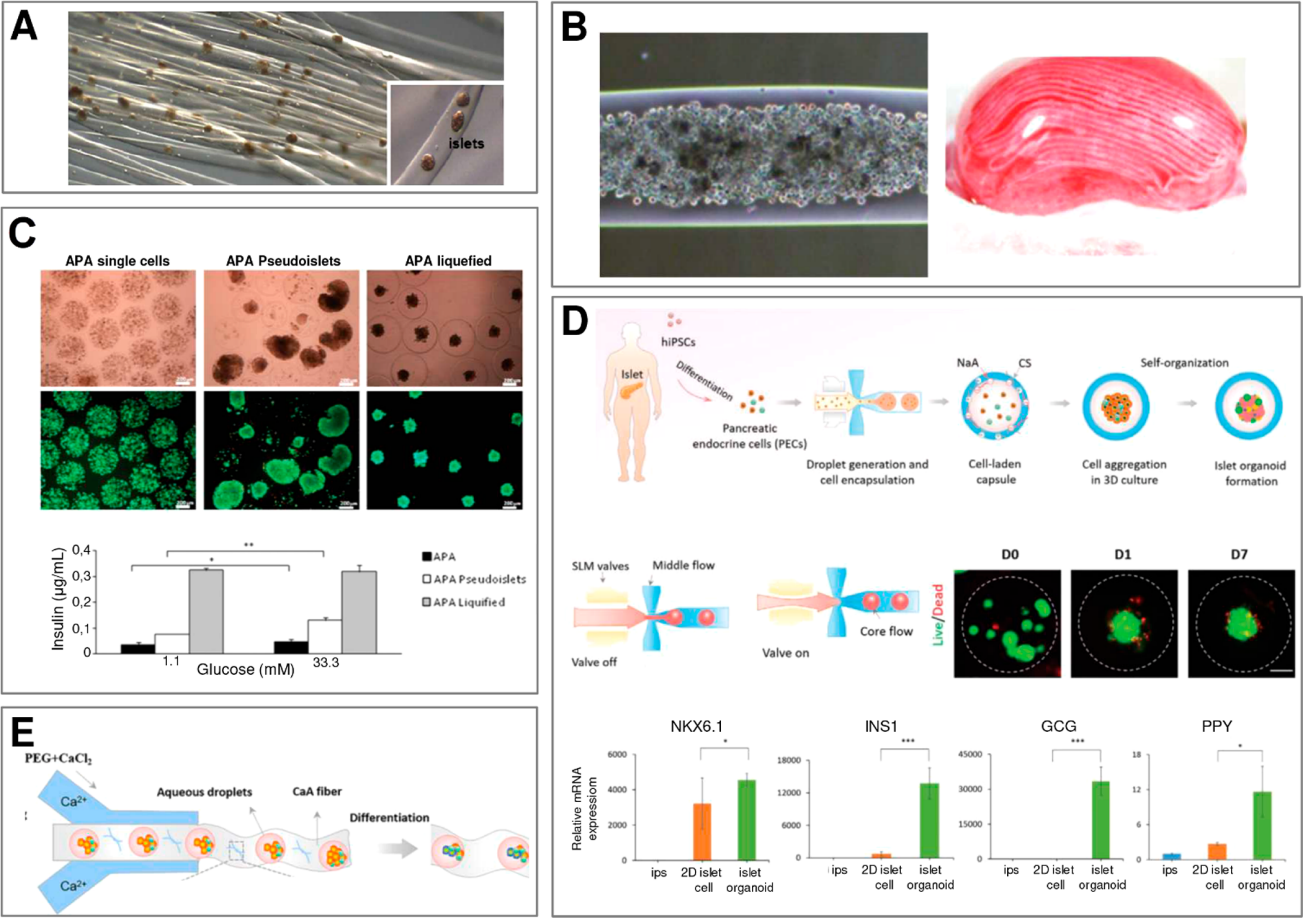
Microfluidics-assisted pancreatic microtissues. (A) Collagen-enriched core–shell fibers encapsulating rat pancreatic islets. Adapted with permission from ref (386). Copyright 2013 Elsevier. (B) Core–shell fiber encapsulating dissociated primary rat islet cells. Adapted with permission from ref (45). Copyright 2013 Nature Publishing Group. (C) Morphology and viability of 1.1B4 cells, forming microtissues in various encapsulation strategies visualized using bright field and fluorescence imaging (top and middle panels), and the corresponding insulin secretion at day 7 (bottom panel). Adapted with permission from ref (161). Copyright 2018 Taylor and Francis. (D) Protocol of islet organoid aggregation from pancreatic endocrine progenitor cells (predifferentiated from hiPSC) in core–shell capsules with liquid core generated using all-aqueous microfluidics (top panel). Dextran-enriched alginate droplets generated with a pressure-controlled single-layer membrane (SLM) valve (middle panel, left) are interfacial cross-linked with chitosan, which leads to capsules with ultrathin shells. Cells aggregate into a spheroid within 1 day in culture (middle panel, right). The expression of beta cell-associated transcriptional factor marker (NKX6.1) and the pancreatic endocrine hormone genes (INS, GCG, PPY) examined using real-time PCR (“ips” stands for undifferentiated hiPSC). Adapted with permission from ref (217). Copyright 2020 Wiley. (E) Schematics of all-aqueous generation of alginate fibers filled with droplets encapsulating pancreatic cells (from hiPSC). Cells aggregate and further differentiate into islet organoids. Adapted with permission from ref (68). Copyright 2021 American Chemical Society.

The pioneering works demonstrating beta cell encapsulation in core–shell fibers were published nearly a decade ago by the Lee group^386^ and the Takeuchi group.^45^ Jun et al.^386^ encapsulated nondissociated rat islets inside collagen-enriched alginate fibers (Figure 24A), whereas Onoe et al.^45^ demonstrated encapsulation of primary islet cells which aggregated inside alginate fibers to form islet cell fibrous organoids (Figure 24B). Both studies reported successful in vivo effective treatment of diabetes quantified via low blood glucose levels for up to several weeks after transplantation in diabetic mouse or rat.

More recently, Acarregui and colleagues^161^ conducted a study comparing pancreatic cell culture in various types of microcarriers, in particular under dispersed and aggregated cellular conformations using either simple hydrogel beads or core–shell structures with a liquid core, respectively. The hybrid 1.1B4 insulin-secreting cells were encapsulated in alginate capsules via electrodripping. The capsules were subsequently coated with a layer of poly-l-lysine and another alginate layer, resulting in formation of alginate-poly-l-lysine-alginate (APA) capsules. Upon liquification of the alginate core via addition of sodium citrate, the encapsulated cells aggregated and formed islet-like spheroids within 7 days of encapsulation. As compared to cells encapsulated in simple alginate beads (“APA single cells”) or to those preaggregated into pseudoislets and then encapsulated (“APA pseudoislets”), the islets cultured in liquid-core APA capsules (“APA liquified”) released more insulin under glucose stimulation (Figure 24C). However, it was not specified if such enhanced performance was due to higher cell number (faster cell proliferation) or rather due to higher insulin secretion per cell. Finally, insulin concentration and response to glucose stimulation in recirculating media were found to gradually increase with time, indicating a possible flow-mediated paracrine effect in the studied system.

Liu and co-workers^217^ (Figure 24D) from the Qin group reported encapsulation of human islet cells, obtained via predifferentiation from human-induced pluripotent stem cells (hiPSCs), in core–shell capsules with an ultrathin semipermeable shell and a liquid core. The authors used an all-aqueous approach in which the external phase was purely aqueous, while the droplet and the shell phases were doped with dextran and PEG, respectively, to support phase separation. After encapsulation, the cells formed spheroids and subsequently self-organized into functional islet organoids. The artificial islets maintained insulin secretion for 7 days and positively responded to glucose stimulation under GSIS test. The organoids exhibited high expression levels of pancreatic endocrine hormone genes (INS, GCG, PPY) (Figure 24D) and beta cell-associated transcriptional factor marker (NKX6.1), all significantly higher than in undifferentiated cells or in 2D culture. Alternatively, as demonstrated later by the Qin group, multiple functional hiPSCs-derived islet organoids can also be encapsulated postaggregation inside a single large (>2 mm in diameter) core–shell capsule consisting of an aqueous core and alginate/PEI semipermeable shell.^383^

The all-aqueous approach was also exercised by the same group to generate aqueous-droplet-filled hydrogel fibers (ADHFs), with beta cells forming stable and uniform organoids within the droplets^68^ (Figure 24E). An important advantage of the fiber-based approach was the possibility of controlling the distance between the organoids, which helped to reduce hypoxia in the multispheroid culture.

**Figure 25:**
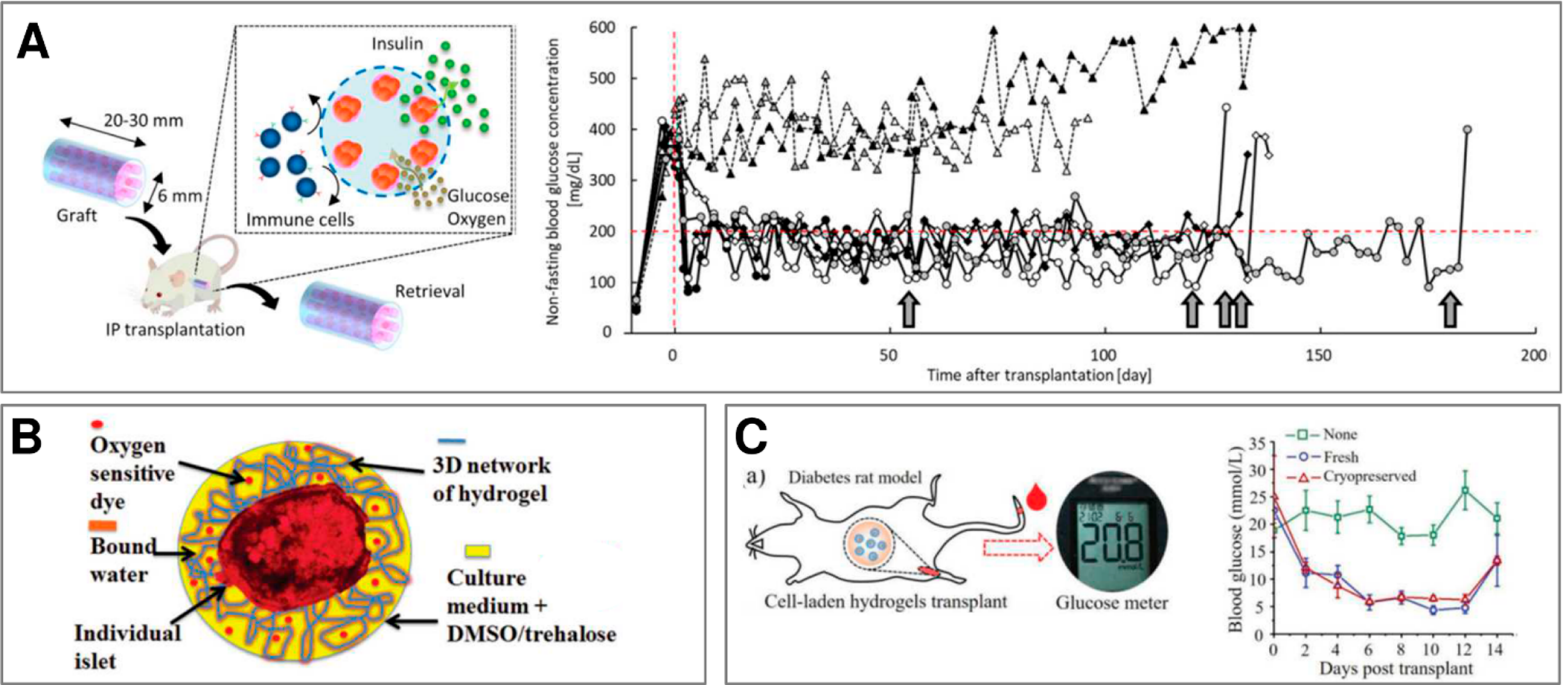
Microfluidics-assisted pancreatic microtissues (continued). (A) Implantation of core–shell fibers with multiple cores in lotus-root like (LENCON) topology encapsulating human beta cells resulted in decreased blood glucose concentration in diabetic mice for up to 6 months. Arrows indicate the time of graft retrieval. Upper curves correspond to nontreated mice. Adapted with permission from ref (51). Copyright 2021 The Authors. (B) Schematics of single-islet-based quality control assay of cryopreserved pancreatic islets with functionalized hydrogel microcapsules. Fluorescent oxygen-sensitive dye (FOSD) serves as quality indicator, while trehalose improves islet functionality. Adapted with permission from ref (384). Copyright 2016 Wiley. (C) Blood glucose level in diabetic rat after implantation of previously cryopreserved islets encapsulated in hydrogel microcapsules, with cold-responsive nanocapsules releasing trehalose during the cryo-stage. Adapted with permission from ref (385). Copyright 2019 Wiley.

Extensive in vivo studies relying on a modification of the core–shell fiber approach have been very recently proposed by Ozawa and colleagues^51^ from the Takeuchi group, who generated Ba-alginate “macro” fibers of 6 mm in diameter with six cell-laden laminin-enriched alginate cores distributed along the fiber, forming a cross-sectional pattern with 6-fold symmetry called LENCON, an acronym for “lotus-root-shaped cell-encapsulated construct”, such that the cores were located close to the surface of the fiber (Figure 25A). Such configuration warranted sufficient oxygen supply to the cells encapsulated in the cores, thus helping to avoid hypoxia, while addition of sodium hyaluronate to the shell had an anti-inflammatory effect. At the same time, the use of such a large object tremendously increased its stability in vivo. The therapeutic potential of the implanted LENCON grafts was demonstrated by nonfasting blood glucose concentrations in immunocompetent mice, which remained at twice-lower levels then in nontreated mice for up to 180 days after implantation. The grafts were shown to maintain functionality (in terms of elevated levels of human C peptide in serum) and retrievability for over 1 year after transplantation. The study demonstrated that the LENCON graft can be a powerful tool with minimal risks for the treatment of T1DM. The authors suggested that the strategy could also be potentially used in other types of cell therapies that require graft retrievability.

Finally, we shortly review the recent efforts aimed at resolving another major challenge in islet transplantation, which is the long-term cryopreservation of the islets and their reliable quality control, especially at a single-islet level, as a step toward “islet banks”. Recently, a new method of efficient quality control based on cryopreserved microcapsules has been proposed by Chen et al.^384^ The authors reported a droplet microfluidic device for encapsulating individual rat pancreatic islets inside porous alginate hydrogel capsules, with fluorescent oxygen-sensitive dye (FOSD) immobilized in the shell to measure the real-time oxygen uptake of individual islets as a functionality indicator after cryopreservation (Figure 25B). The authors demonstrated that the encapsulation process did not affect the cells, whereas the addition of trehalose to the cryopreservation medium improved the functionality of the cryopreserved islets.^384^ Another method, developed by Cheng and co-workers,^385^ aimed at improving the efficiency of cryopreservation via combining cold-responsive nanocapsules (CR-NCs) and microfluidic encapsulation to facilitate CPA (trehalose)-based cryopreservation of beta cell-laden alginate capsules. Beta cells were encapsulated in calcium alginate hydrogel (CAH) using centrifugal microfluidics. After cryopreservation with a commonly used slow freezing and thawing protocol, the cells expressed insulin after implantation in vivo, as measured by blood glucose level in a diabetic rat (Figure 25C). In summary, the authors showed that replacing the traditional toxic cryoprotectant agents such as DMSO with trehalose provides a more biocompatible route toward beta cell cryopreservation.

In summary, encapsulation of beta cells for treatment of diabetes shows great promises and recent in vivo models demonstrate significant success in long-term treatments based on implanted islet cell-laden functional microgels. Cell therapies based on encapsulation of human induced pluripotent stem cells in combination with well-established protocols allowing for long-term cryopreservation of the islets without affecting their paracrine functions appear as a realistic future cure for type 1 diabetes. Current studies should focus on development of methods of encapsulation of larger number of cells and strategies aiming at optimization of the size of the graft for most sufficient insulin production in the type 1 diabetes mellitus patients. Such new strategies are necessary to eventually translate the findings of *laboratoryresearch* into *clinical practice*.

#### 5.2.3 Neural Tissue

Unlike any other cells, neurons form highly organized structures of unique morphological and physicochemical properties, making the nervous system one of the highest biological complexity. The vertebrate nervous system consists of two main parts: the central nervous system (CNS) composed of the brain and spinal cord, and the peripheral nervous system (PNS), whose primary role is to provide the communication between the CNS and the body by innervating all organs, limbs, and skin.

Microfluidics-based neural tissue engineering aims at providing a permissive environment for differentiation and growth of neural cells^88,108,276^ and at developing organized hydrogel scaffolds that support neuronal growth, which could be further used either (i) to model neural tissue organization and function,^45,82,88,114,274,276^ including cytotoxicity assays or drug screening tests, or (ii) to develop implantable scaffolds for restoration of the CNS or PNS functions.^50,221^ Compartmentalized topologies of the hydrogel structures allow for precise control over the spatiotemporal distribution of chemical and physical cues and implementation of the coculture systems. This is of particular importance when designing tissue repair strategies where the presence of supporting cells (e.g., Schwann cells in PNS repair therapies^387^) or growth factors^388^ plays an important role in promoting neural cell survival, growth or migration and ability to integrate with the host neurons and to form a functional neural network. Finally, the supporting cells allow for the improvement of the neural functions, which could be impaired as a result of progression of the neurodegenerative diseases such as Alzheimer’s and Parkinson’s or injuries of the CNS or PNS.

One of the limitations in the development of human neural microtissues is the availability of neural cells. Neurons become terminally differentiated and postmitotic very early during development,^389^ meaning that neural cells that divide and mature during embryonic neurogenesis lose their ability to proliferate in postnatal life and therefore must remain alive and functional for decades. Accordingly, because the use of human fetal neural tissues is unsustainable due to the ethical and availability concerns, the source of choice for human neurons in vitro are neural stem cells or more accessible hiPSCs that can be obtained directly from a patient.^390^

**Figure 26:**
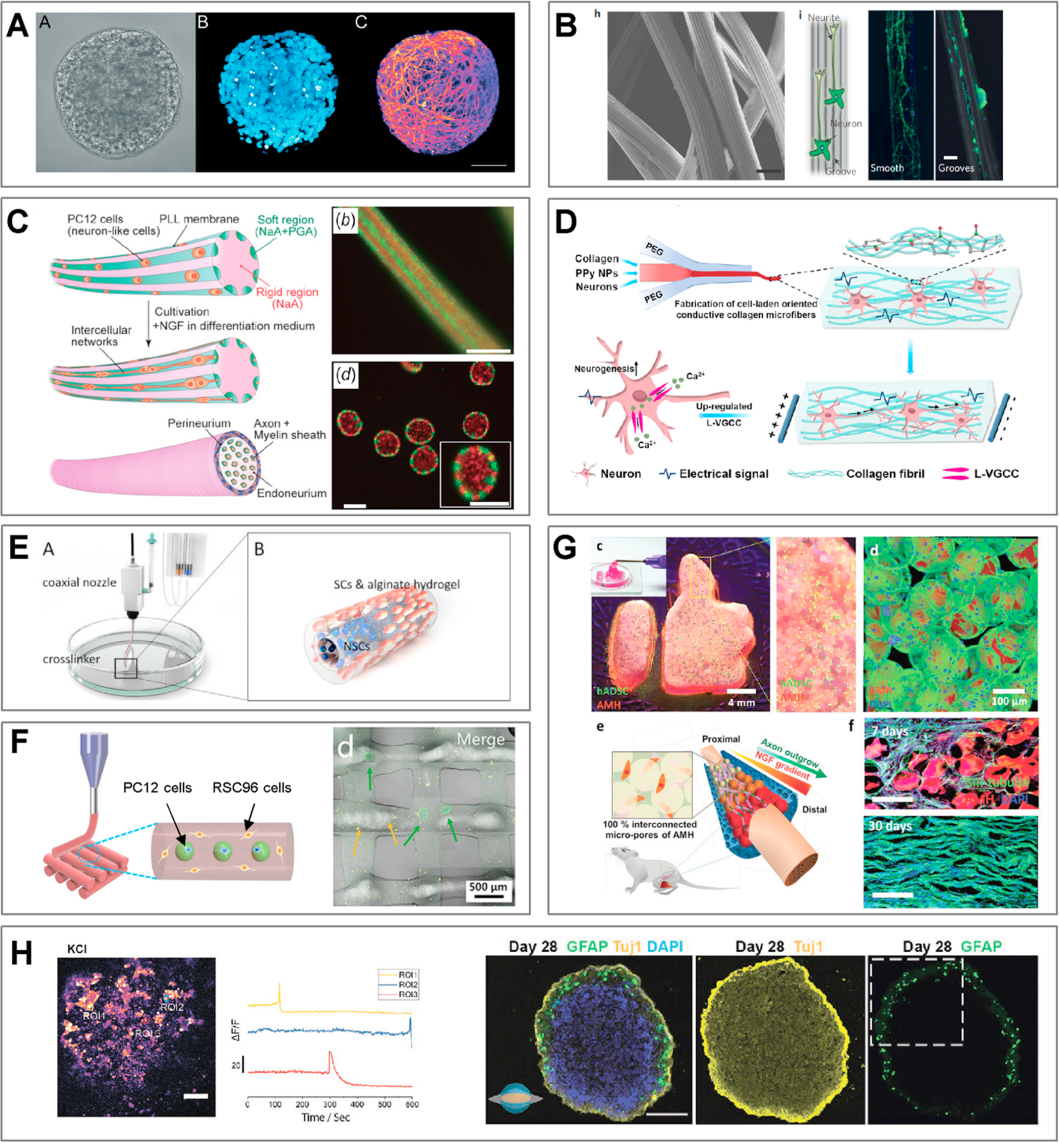
Microfluidics-assisted neural microtissues. (A) Core–shell capsules for neural cell differentiation. A bright field image of neural capsule (left) and fluorescence confocal microscopy images with DAPI staining of the nuclei (middle) and with tubulin subunit beta3 staining of mature neurites (right). Scale bar 50 μm. Adapted with permission from ref (108). Copyright 2016 The Royal Society of Chemistry. (B) Grooved microfibers fabricated using wet spinning, scale bar 20 μm (left), schematic of neuron alignment on a grooved fiber (middle), and fluorescence micrograph of neurons on a fiber without grooves and on a grooved fiber, scale bar 50 μm (right). Neurofilament (green) and nuclei (blue). Adapted with permission from ref (82). Copyright 2011 Macmillan Publishers. (C) Complex hydrogel microfiber composed of a rigid region and the cell encapsulating soft regions for guiding cell proliferation and forming intercellular networks and a schematic image showing a nerve fascicle, a constituent of a broader nerve bundle. NaA, sodium alginate; PGA, propylene glycol alginate; NGF, nerve growth factor (left). Fluorescence micrographs showing the four-region (side view) and the eight-region hydrogel fiber (cross-sectional view). Scale bar 100 μm (right). Adapted with permission from ref (274). Copyright 2014 IOP Publishing Ltd. (D) Schematic illustration of constructing cell-laden polypyrrole(PPy)-incorporated conductive collagen hydrogel core–shell microfibers. Adapted with permission from ref (276). Copyright 2019 American Chemical Society. (E) Schematic views of extrusion setup and core–shell microfiber for coculture of Schwann cells and neural cells. Adapted with permission from ref (114). Copyright 2019 Oxford University Press. (F) The design of a multiscale composite scaffold with PC12 cells and RSC96 cells for peripheral nerve tissue restoration (left) and bioprinted mesh scaffold within PC12 cells and RSC96 cells (right). Adapted with permission from ref (221). Copyright 2020 Elsevier. (G) Adaptable microporous hydrogels (AMHs) for the creation of injectable scaffolds for nervous tissue restoration (top panel). Schematic illustration of AMH with a propagating NGF-gradient for directed and accelerated axonal outgrowth in vivo and microscopic images of parallel growth of the axons through the scaffolds, scale bars 200 μm (bottom panel). Adapted with permission from ref (50). Copyright 2019 The Authors. Published by Wiley-VCH. (H) From left to right: fluorescence intensity maps obtained via calcium imaging after KCl treatment of neurons (scale bar 100 μm), single-cell signals, and sections through a central plane of the organoid with different indicated immunostainings at day 28. Neurons were immunostained with TUJ1 and astrocytes with GFAP. Note that hNSCs have migrated toward the astrocyte compartment, whereas astrocytes remained restricted in the outer compartment. Scale bar are 200 μm. Adapted with permission from ref (88). Copyright 2020 The Authors.

Microfluidic encapsulation of hiPSC was first demonstrated by Alessandri and co-workers,^108^ who developed core–shell capsules, dedicated for neural stem cell culture and differentiation consisting of alginate shell, intermediate layer of Matrigel, and cell suspension in the core (Figure 26A). The inner surface of the alginate shell served as a template for the anchorage of Matrigel, which in turn provided the surface for cell attachment. Neural stem cells attached to the layer of Matrigel, leaving the core of the capsule empty. This prevented spontaneous aggregation of cells and spheroid formation, which for large spheroids would otherwise limit diffusion of oxygen and nutrition to the cells in the inner part of the aggregate, thus possibly resulting in cell necrosis. In the study, human neural stem cells (hNSC) derived from human induced pluripotent stem cells (hiPSC-BC-1 line) were properly differentiated into mature Map2 and tubulin-β-3 positive neural cells.

To develop a neural microtissue model that closely resembles physiological conditions, one must consider not only the native environment of the cells but also their morphology, function, and spatial organization. In vivo, single neurons organize into bigger and more complex linear tissue structures, forming bundles of axons which can be referred to as nerves or tracts. Microfluidic platforms have been shown to be effective in generating liner neural networks ranging from simple core–shell fibers^45,276^ to patterned structures for guided cell growth and controlled neurite outgrowth^82,274^ and complex coculture systems.^88,114,221^ The high sensitivity of neural cells to topographic and mechanical cues of the matrix can be used to locally guide neural tissue development. Such strategy was exploited using grooved fibers, whose longitudinally grooved surfaces promoted selective adhesion and alignment of neural cells along the fiber^82^ (Figure 26B). Recently, an interesting approach has been presented by Kitagawa et al.^274^ A multilayer microfluidic system consisting of an array of micronozzles was developed to generate hydrogel microfibers with complex cross-sectional morphologies (Figure 26C). Adjustments of the micronozzle array geometry and tuning of the rates of flow of the precursor solutions were used to control the morphology of the fibers. Introduction of two different hydrogel precursor solutions allowed for formation of compartmentalized fibers with heterogeneous mechanical properties. In particular, alginate fibers with longitudinal grooves filled with a softer hydrogel were demonstrated. These softer regions (made of alginate with propylene glycol alginate) were used to encapsulate PC12 neural model cells. The cells seeded in the parallel soft regions proliferated and formed intracellular neural-like networks along the fiber due to the physical restrictions imposed by the relatively rigid surrounding regions. The grooved fiber morphology could be seen as structurally analogous to the complex nerve bundles found in vivo.

Functionality of the neural microfibers can be improved by introducing electroconductive environment, hence enhancing transmission of intercellular electrical signals and promoting effective delivery of external electrical cues to the cells. Addition of electroconductive polypyrrole nanoparticles to the collagen core of the cell-laden core–shell microfibers improved cell extension and upregulated neuron-related gene expression, including expression of voltage-gated calcium channels^276^ (Figure 26D). Moreover, application of an external electrical stimulation further enhanced gene expression of tubulin-β-III, NF-66, and Cacnb3 neuronal markers in PC12 neural model cell.

Despite high morphological and physicochemical relevance, thus far generated structures^274,276^ have not yet fully recapitulated the biological structure of the nerve. It should be noted that in physiological conditions, the neural cells both in CNS and PNS are surrounded by many supporting cells. For example, the Schwann cells which wrap around axons of motor and sensory peripheral nerves form insulating myelin sheets to increase conduction velocities along myelinated axons and provide trophic support and cushioning to the unmyelinated ones.^114,391^ Liu et al.^114^ demonstrated that encapsulation of RSC96 Schwann cells in the shell of NE-4C neural stem cell-laden core–shell microfibers increases cell proliferation and upregulates the expression of tubulin-β-III, thus enhancing neuronal differentiation (Figure 26E).

In addition to formation of cell-laden core–shell microfibers with encapsulated different cell types, microfluidics allows preparation of multicompartment coculture composite scaffolds for investigation of the Schwann cell–neuron interactions. For example, a 3D bioprinted multiscale scaffold based on a modular bioink has been developed to mimic the complex functions of multicellular neural tissues^221^ (Figure 26F). Gelatin methacryloyl (GelMA)/chitosan microspheres (GC-MSs) enriched with NGF were prepared by using microfluidic flow-focusing device, cross-linked under UV irradiation to form the monodisperse spherical microgels, and coated with PC12 cells. Subsequently, beads were suspended in aqueous solution of GelMa and RSC 96 neural Schwann cells generating a multiscale neural bioink with great potential applications in peripheral nerve tissue engineering.

An alternative approach to generate compartmentalized microfluidic-based NGF loaded gelatin/chitosan **3D scaffolds** for restoration of the PNS functions has been presented by Hsu and co-workers.^50^ Hydrogel beads of photo-cross-linkable gelatin methacrylate (GelMA) and chitosan oligomer–methacrylate (ChitoMA) were used to fabricate adaptable microporous hydrogel structures (Figure 26G). Cross-linked hydrogel structures were shown to support growth of PC12 cells in vitro and in vivo, where they promoted sciatic nerve regeneration in rodents after injury. Additionally, to induce directional growth of regenerating axons the NGF gradient was applied in a conduit by mixing the beads loaded with 200, 150, and 100 ng mL^–1^ NGF and placing the three batches in a tube one by one to form three sections. Seven days of postimplantation nerve filaments were found with gradually decreased probability in the corresponding proximal, middle, and distal sections of the nerve gap, suggesting steady and directional axonal outgrowth.

Whereas the Schwann cells act as the main supporting cells in the PNS, in the CNS, astrocytes regulate neuronal cell outgrowth and migration. The neuronal cell migration is particularly important during cortical development and allows distinct cell types generated in different brain regions to settle in the cerebral cortex, forming unique cytoarchitecture and layers of highly specialized neuronal cell subpopulations critical for memory formation, language, vision, attention, and other intellectual activities. Studying cortical development and particularly cortical neurons migration in vitro requires prepositioning of the cells in the initial 3D culture scaffold, a task which still remains technically challenging. In a recent study, Zhou and colleagues^88^ proposed a lipid-bilayer-supported droplet bioprinting technique to control spatial patterning of the human cortical cells in the ECM. During the printing process hNSC-laden, ECM-containing droplets were ejected into lipid-containing oil bath, where the amphiphilic lipid molecules spontaneously formed monolayers at their the droplet surface. Next, the cell-laden droplets were positioned next to each other so such lipid bilayers formed at their contacts. The resulting droplet–droplet adhesion supported the 3D architecture of the printed droplet network and allowed for cell compartmentalization and patterning. The structures cross-linked upon incubation at 37 °C before their transfer to cell culture medium. Printed NSCs remained viable, differentiated properly, and projected neuronal processes across droplet boundaries forming functional neuronal networks. Fluorescent intensity maps obtained via calcium live imaging after KCl treatment showed spontaneous oscillation of Ca^2+^ ions (Figure 26H). To study the role of astrocytes in cortical neuron migration and maturation, the authors generated prepatterned 3D matrices using two populations of droplets containing either hNSCs or astrocytes (Figure 26H). The spatial patterning was designed to mimick in vivo 3D structure, where astrocytes are distributed in different layers of cortex while remaining in close contact with the neuronal cells. The observations revealed that astrocytes promote neuronal cell migration into the astrocyte domains and induce formation of axon bundles, a feature crucial for fast interneuronal communication in the brain. The astrocytes rather preferred to remain segregated from neurons and did not change their initial location. Altogether, the study demonstrated that the lipid-bilayer-supported bioprinting technique can be successfully used to spatially position distinct cell types and construct various 3D tissue models, including human brain, and guide their self-organization.

All in all, despite the recent efforts and considerable progress in the field of microfluidic-based neural tissue engineering, creating highly biomimetic neuronal microtissues still remains a challenge. Majority of the developed strategies rely on the use of PC12 neural model cells, which are highly accessible and easy to propagate but do not accurately mimic the heterogeneity of neuronal cells in vivo. Therefore, future research should aim at further development of NSC-based models by creating effective and high-throughput microfluidic technologies for NSC differentiation and performing more detailed functional analyses of the created models. Moreover, current studies mostly aim at development of the PNS rather than CNS microtissue models. This is likely related to higher complexity of the brain tissue and a need to accommodate higher number of supporting cell types, including various types of microglia cells, in the models. In the future, the development of more realistic CNS tissue models could open the way toward better understanding of the neurodegenerative diseases such as Alzheimer’s and Parkinson’s diseases as well as toward new neuronal tissue restoration therapies, which are of particular need for patients after spinal cord injury or brain injury.

#### 5.2.4 Cardiac Tissue

The main function of the heart muscle is to deliver oxygen- and nutrient-rich blood to tissues and organs via blood circulation. The flow of blood is supported by a highly specialized cardiac muscle, which unlike skeletal muscle can contract spontaneously with an intrinsic rhythm set by pacemaker cells in the sinoatrial node, and thus can generate the involuntary heart contractions known as the heartbeat. Heart muscle damage, acute or chronic, is one of the leading causes of heart failure, making cardiovascular diseases one of the major cause of death worldwide.^392^ The main problem resides in the lack of endogenous regenerative capacity of adult heart muscle caused by inability of cardiac myocytes to divide and replace injured cells. Despite the presence of resident cardiac stem cells in the heart, their population is apparently too small to facilitate any repair processes.^393^ Instead, after myocardial infarction, the regions of damaged myocardium are replaced with fibrillar collagen and/or fibroblast-like cells forming the scar tissue.^394^ The scar tissue in the injured heart prevents further damage but lacks the ability to contract and as such remains nonfunctional.

According to World Health Organization (WHO) reports, myocardial infarction is one of a leading cause of heart tissue damage. To restore the heart function after myocardial infarction, one would have to either replace formed scar tissue with functioning myocardium or develop a clinical intervention that would prevent scar tissue formation. Compartmentalized microscaffolds have a great potential in providing a microenvironment for differentiation of stem cells into cardiac lineages^47,107,111,187^ that could be used both in basic biological studies as well as in regenerative medicine as building blocks of multidimensional and implantable hydrogel structures mimicking heart tissue and/or providing a permissive environment for myocardium regeneration after injury.^45,48,203,277,279^

**Figure 27:**
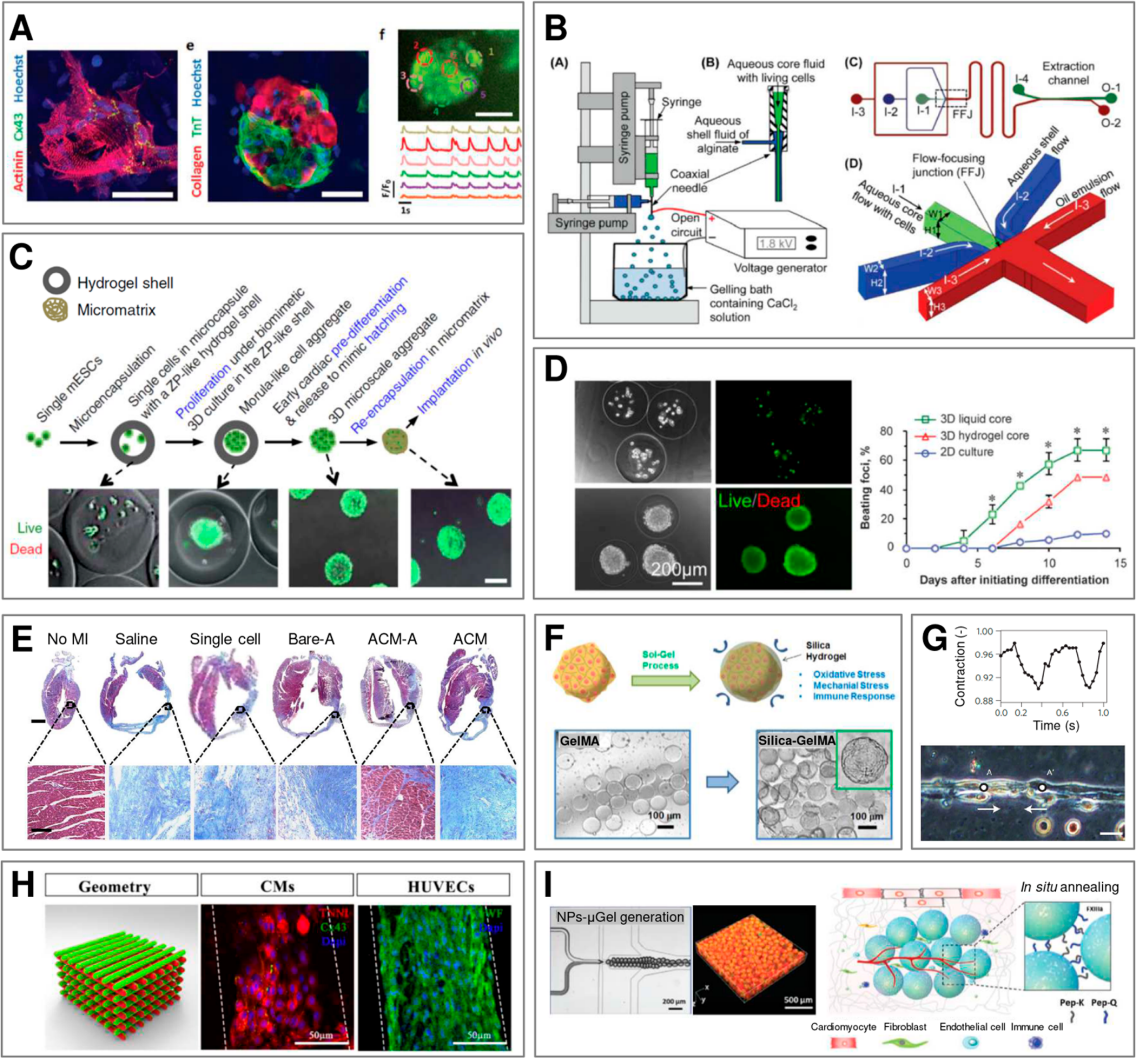
Microfluidics-assisted cardiac microtissues. (A) Beating cardiac foci generated using iPSCs-derived cardiomyocytes. Immunocytochemistry (left and middle panel) and electrical signal analysis performed by calcium imaging on day 7 (right panel). Scale bars 50 μm. Adapted with permission from ref (238). Copyright 2020 Wiley-VCH. (B) Microfluidic approaches for generation of bioinspired core–shell microcapsules: a schematic overview of the coaxial electrospray setup (left side) and of the microfluidic channel system (left side). Adapted with permission from ref (187). Copyright 2016 American Chemical Society. (C) A schematic illustration of the embryo-hatching-inspired procedure for producing 3D microscale constructs of mESCs, together and the corresponding experimental images. Scale bar 100 μm. Adapted with permission from ref (47). Copyright 2016 The Author(s). (D) mESCs under the bioinspired 3D culture in the complex core–shell microcapsules (left side) and cumulative percentage of beating foci on each day, showing that the cell aggregates from the miniaturized 3D liquid core have significantly more beating foci than those from 3D hydrogel and 2D culture. Adapted with permission from refs (111 and 187). Copyright 2016 American Chemical Society and 2014 The Royal Society of Chemistry. (E) Cardiac regeneration with the alginate–chitosan micromatrix (ACM) encapsulated predifferentiated aggregates. Low-magnification sagittal micrographs of Masson’s trichrome stained tissue sections (top row) and zoom-in views of the left ventricular wall (bottom row), showing extensive fibrosis in the myocardial infarction hearts treated with saline, single cells, and ACM-encapsulated cell aggregates after releasing from microcapsules (Bare-A) and after re-encapsulating them in ACM (for ACM-A) or materials alone (ACM). Adapted with permission from ref (47). Copyright 2016 The Author(s). (F) Schematic description of fabrication of protective silica hydrogel shell on the GelMA microgels (top row) and optical microscopic images of GelMA microgels and silica-coated GelMA microgels (bottom row). Adapted with permission from ref (282). Copyright 2013 American Chemical Society. (G) Core–shell fiber loaded with primary cardiomyocytes and its spontaneous contraction. Adapted with permission from ref (45). Copyright 2013 Nature Publishing Group. (H) Multicellular 3D bioprinted cardiac tissue constructs of Janus topology stained for TNNI (red) and Cx43 (green) expressions in CMs and vWF (green) in HUVECs. Adapted with permission from ref (277). Copyright 2018 The Author(s). (I) Cardiac drug-releasing MAP scaffolds for MI therapy self-assembling into 3D structures. Adapted with permission from ref (48). Copyright 2020 Wiley-VCH.

The replacement of scar tissue requires an efficient, reproducible, and scalable system for culturing and differentiation of pluripotent stem cells into functional cardiomyocytes. Microfluidic-based approaches succeeded in providing advanced systems for high-throughput generation of functional beating cardiac foci (Figure 27A)^47,107,111,187,238^ that can be implanted in vivo to promote cardiac tissue regeneration.^47^ Biomimetic core–shell capsules inspired by native milieu in a prehatching embryo were generated at high throughput by using nonplanar microfluidic flow-focusing devices^107^ as well as coaxial electrospray systems^47,111^ (Figure 27B). The liquid core of the capsule supported cell mobility and allowed spontaneous cell aggregation, while the external alginate shell served as a physical boundary protecting the cells from the external environment^47,107,111,187^ (Figure 27C). The shell was demonstrated to be enzymatically digestable. The released differentiated cell aggregates were subsequently re-encapsulated inside a biocompatible and biodegradable micromatrix suitable for injection.^47^ Embryonic cells cultured in a liquid core of the core–shell capsules maintained their stemness for longer time and developed significantly higher capability of directed cardiac differentiation as compared to the cells encapsulated in a cross-linked hydrogel core or cultured in conventional 2D systems. This was signified by higher expression of cardiac genes such as Nkx2.5 and cTnT and reduced expression of mesodermal markers such as brachyury and T.^47,107,111^ More efficient differentiation of stem cells resulted in a higher percentage of spontaneously beating cardiac foci, thus demonstrating high potential of compartmentalized hydrogel capsules in designing and optimizing systems for reproducible generation of cardiac tissue (Figure 27D) and their application in restoration of heart functions after myocardial infarction and in cardiac functions modeling (Figure 27E).

The survival of cardiac cells and their functional performance can be additionally improved by applying degradable protective shells^282^ or by using coculture systems.^395^ For example, silica coating of cell-adhesive GelMa core with cardiac precursor cells on the microgel surface was shown to provide significant protection against oxidative stress, which is often encountered during and after injection into host tissue^282^ (Figure 27F).

In vivo, the individual cardiac myocytes branch and form multiple overlapping arrays of muscle cells fibers arranged in different circumferential orientations. Individual branched cardiac myocytes are linked to each other at both ends through intercalated discs, called desmosomes. The compact end-to-end orientation of cardiac myocytes has been suggested to enhance their electrophysiological connection and functions.^396^ Onoe et al.^45^ developed a microfluidic device with double-coaxial laminar flow that allows for generation of long core–shell microfibers that aimed at reconstituting the intrinsic heart morphology and function (Figure 27G). The core of the fabricated microfibers consisted of cardiac cells and ECM proteins in the pregel state and was encapsulated by the mechanically stable alginate shell. The shell prevented the ECM proteins from diffusing away from the core until the core gelled. In the final stage, alginate shell was enzymatically digested, releasing the self-contracting cardiac microfiber.

Beating cardiac microfibers aim at mimicking the structure of the myocardium tissue, however, the morphological relevance of such generated microtissue is limited because, unlike skeletal muscles, cardiac muscle cells do not form polynucleated long cylindrical fibers but rather a mesh of tightly connected single-branched cells. Thus, it may seem that the high structural complexity of myocardium may narrow the application of linear structures in microfluidic-based cardiac tissue engineering. However, to increase the physiological and morphological relevance of fiber-based models, the simple linear structures can be arranged into 3D structures of higher topological and cellular complexity. Maiullari et al.^277^ presented an innovative approach to fabricate 3D multicellular mesh-like scaffolds for vascularized heart tissue engineering (Figure 27H) via encapsulation of cardiomyocytes derived from iPSCs and HUVECs in hydrogel strands containing PEG-fibrinogen and alginate, extruded through microfluidic printing head. Alginate was used to precisely control fibers deposition. The effect of spatial organization of HUVEC and iPSC-derived cardiomyocytes on vascular network formation and on the differentiation and organization of cardiomyocytes was studied by bioprinting structures of various geometries including Janus fibers, in which the two different cell lines were precisely compartmentalized within each laid fiber and structures generated by alternating layers of HUVEC with iPSC-derived CM layers. Interestingly, the authors showed that differences in topology of the generated microtissues affect expression levels of angiogenic (hif-1α, e-nos, pgk1, vegf, kdr, e-cad), apoptotic (bcl2), and cell proliferation factors (ccnd1) as well as the orientation of engrafted cells after implantation of the scaffold. Moreover, the addition of HUVECs supported the integration of the engineered cardiac tissue with host’s vasculature.

Another approach to restore cardiac functions after injury is based on rapid clinical interventions aiming to reduce local inflammation and scar tissue formation, hence preventing heart damage progression. In such a case, core–shell microcapsules^348^ or microporous 3D hydrogel scaffolds^48,279^ can be used to deliver drugs, promote cell survival and growth, and reduce heart tissue fibrosis. Core–shell drug delivery systems, with a large, drug-laden liquid core and a polymeric shell regulating the release have been shown to be particularly effective in sustained delivery in the cardiac tissue.^348^ For example, microcapsules fabricated using polyethylene glycol diacrylate (PEGDA) and dextran, loaded with vascular endothelial growth factor (VEGF) and platelet-derived growth factor (PDGF) released therapeutic agents for over 30 days.^348^ Intramyocardial injection of VEGF- and PDGF-loaded hydrogel capsules into rat with acute myocardial infarction improved cardiac function and significantly reduced fibrosis of the infarct region.^348^ An interesting approach has been recently presented by Fang and co-workers^48^ (Figure 27I). A microfluidic device was developed to generate “self-healing” injectable granular hydrogel scaffolds for drug delivery. In particular, a flow-focusing device was used to encapsulate drug-loaded nanoparticles inside (PEG)-based beads sensitive to matrix metalloprotease. Dense suspension of the beads, a granular paste, was injected into the infarcted heart. The drug-loaded beads annealed as the endogenous factor XIIIa activated peptides K and Q present at the bead surfaces, resulting in a porous drug-releasing scaffold. Such scaffold provided mechanical support for the seeded cells and promoted cell migration after implantation in vivo while suppressing fibrosis and immune responses. Accordingly, the injectable bead-based scaffolds were demonstrated as a promising strategy toward cardiac regeneration.

In conclusion, topological heart microtissues based on microgels generated using microfluidics certainly have a great potential for in vitro and in vivo applications. They allow for robust differentiation of stem cells into cardiac lineages and formation of cardiac beating foci at high-yield. These functional “mini-hearts” enclosed in hydrogel droplets may serve as valuable heart models in myocardial drug discovery as well as in studies aiming at understanding fundamentals of heart diseases. Furthermore, the heart microtissues derived from human iPSCs, when combined with vascular microenvironment, promise new therapeutic strategies toward myocardium restoration.

#### 5.2.5 Skeletal Muscle

Skeletal muscle tissue is a contractile tissue strained by tendons attached to bones. The contraction, happening under the control of the somatic nervous system, allows voluntary movements that enable locomotion. Unlike myocardium, skeletal muscles have a high potential for self-repair and can regenerate after minor injuries. However, more severe damages involving volumetric muscle loss and myopathies can cause irreversible loss of muscle mass and function. Skeletal muscles consist of hierarchically organized biological units. On the microscopic level, skeletal muscles are built from bundles of skeletal muscle fibers, so-called myofibers, surrounded by the connective tissue. These myofibers are formed by the fusion of myoblasts into elongated, cylindrical, multinucleated fibers, so-called myotubes, of diameters ranging from 10 to 100 μm (depending on the function and location of the muscle)^397^ and representing the functional unit of the skeletal muscle tissue.

**Figure 28:**
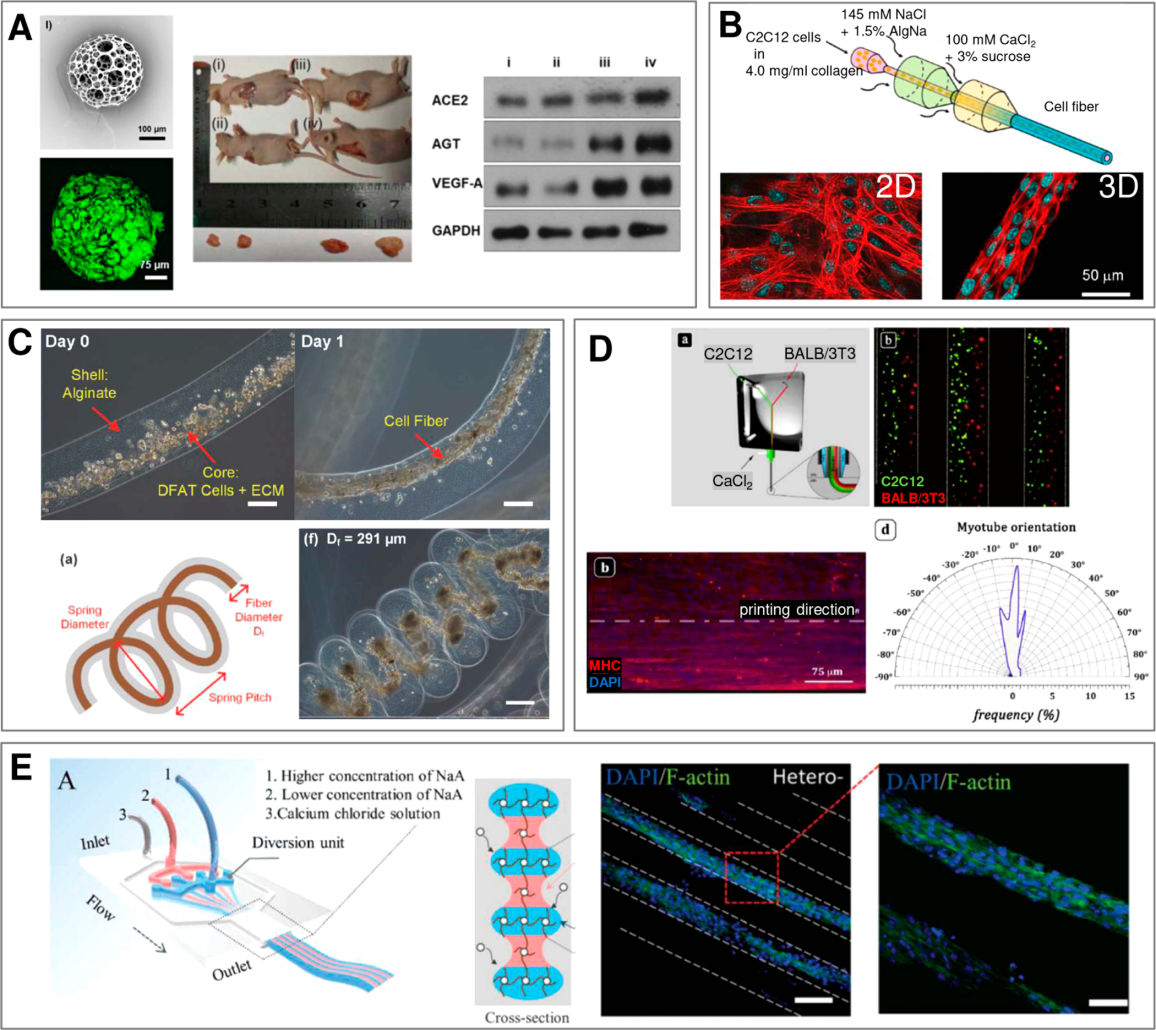
Microfluidics-assisted skeletal muscle microtissues. (A) Scanning electron microscopy image of PLGA porous microbead and confocal laser microscopy images showing the proliferation of C2C12 myoblast cells on the PLGA porous microbeads (left panel). Images showing the growth of myoblasts in mice treated with various samples (i) control (normal saline), (ii) microcarriers (suspended in PBS), (iii) isolated cells (8 × 106/mL suspended in PBS), and (iv) cells-laden PLGA HOPMs (suspended in PBS) (middle panel). Expressions of vascular biomarkers (ACE 2, AGT refers to angiotensin, VEGF-A, and GAPDH) determined by Western blot analysis (right panel). Adapted with permission from.ref (285). Copyright 2019 Wiley-VCH. (B) Schematic for fabrication of C2C12 cell-laden core–shell hydrogel microfibers (top panel). Fluorescent images of rhodamine–phalloidine (red)/DAPI (blue) counterstaining to visualize the actin cytoskeleton of the C2C12 cells. Cells culture in hydrogel microfiber are highly aligned when compared to 2D culture (bottom panel). Adapted with permission from ref (195). Copyright 2019 The Authors. Licensee MDPI. (C) Cell fiber formation. Day 0 image captured immediately following fiber fabrication with single DFAT cells dispersed in ECM proteins encapsulated in the center core of the alginate fiber and a day 1 image showing a section of the formed DFAT cell fiber inside an alginate shell (top panel). Schematic illustration and image of cell-laden spring microfiber (bottom panel). Scale bar 200 μm. Adapted with permission from ref (110). Copyright 2015 The Authors. (D) Schematic of multicellular microfluidic printing head and an image of C2C12 myogenic precursors and BALB/3T3 fibroblasts-laden compartmentalized Janus microfibers (top panel). Myotube alignment in muscle networks obtained in 3D bioprinted constructs after 15 days of in vitro culture (bottom panel). Adapted with permission from ref (73). Copyright 2017 The Author(s). Published by Elsevier Ltd. (E) Schematic diagram of a microfluidic chip with multiple channels for the preparation of grooved microfibers and a fiber cross-section (top panel). Fluorescence images showing the orientation of F-actin (green) and cell nuclei (blue) within cells on heterogeneous grooved microfibers, scale bar 100 μm (bottom panel). Adapted with permission from ref (212). Copyright 2021 The Royal Society of Chemistry.

The native muscle structure, i.e., longitudinal and circumferential architecture of myofibers has been successfully mimicked using topological hydrogel microfibers^73,110,195,398^ and their assembly into muscle-like bundles.^73,212,281^ Some of the structures were tested in vivo, demonstrating great potential in tissue engineering and regenerative medicine applications.^73,398^ Structures of lower dimensionality such as porous beads (Figure 28A) were also used as myoblast carriers, but due to their nonphysiological topology, the bead-based muscle microtissues do not reproduce the native muscle tissue morphology.^285^

Skeletal muscle-mimicking microstructures of linear architecture include a wide spectrum of topologies ranging from simple core–shell microfibers^110,195^ to multicompartment ribbons.^212^ Bansai et al.^195^ demonstrated that C2C12 muscle precursor cells, when encapsulated in the collagen-rich core of a hydrogel core–shell microfiber, indeed reorganized their cytoskeleton along the wall of the hydrogel fiber and formed longitudinal myofiber-like structures (Figure 28B), while the cells cultured in a conventional monolayer exhibited random organization of cytoskeleton. Moreover, the authors showed that cyclic stretch of microfibers promoted elongation and myogenesis of the C2C12 cells and increased the ratio of the mature myotube-like cells. This was possible with the use of the alginate shell which enhanced mechanical stability of the fibers allowing them to withstand the applied mechanical forces.

Biomimetic muscle fibers can be also generated by encapsulation of stem cells inside hydrogel microfibers followed by cell differentiation into muscle cells.^110,398^ Hsiao and co-workers^110^ demonstrated that dedifferentiated fat (DFAT) cells suspended in a mixture of extracellular proteins and encapsulated in the core of alginate–shell microfibers can form functional muscle units (Figure 28C). Upon differentiation and induction to the smooth muscle lineage DFAT cells uniformly aligned along the inner wall of the hydrogel fibers and contracted. This nonaxial contraction gradually led to fiber bending and folding into a helix. Differentiation of DFAT cells into smooth muscle cells was additionally confirmed by measuring the expression of the corresponding markers including ASMA and calponin. Another stem-cell based approach was presented by Kim et al.^398^ A multichannel microfluidic device was used to generate muscle cell-laden hybrid constructs, in which human adipose-derived mesenchymal stem cell (hASC)-laden GelMa beads were encapsulated in the alginate strut, resulting in formation of a hybrid strut containing muscle cell beads that mimicked cell spheroids. The distance between cell beads within alginate fiber was adjusted by controlling the flow rate of the alginate solution. Encapsulated hASC properly differentiated into muscle cells, which was confirmed by high expression of myogenic gene markers such as MyoD, MHC, and MyoG. The muscle regeneration potential of constructs was evaluated by implanting hASC cell-bead-laden struts into volumetric muscle loss injury mouse models. Analysis of tibialis anterior muscle weight, functional testing of the injured limb, and histological stainings showed that hASC-cell-bead-laden struts induced efficient volumetric muscle regeneration when compared to conventionally bioprinted hASC cell-laded fibers.

Differentiation and fusion of myoblasts into myotubes can be enhanced by adding the supporting cells, for example, fibroblasts, which are known to secrete extracellular matrix components and growth factors.^399^ Costantini et al.^73^ designed a multicellular 3D bioprinting system, with a microfluidic printhead allowing extrusion of PEG-fibrinogen-alginate Janus microfibers with C2C12 cells and BALB/3T3 fibroblasts (Figure 28D). The fibers were first quickly gelled in Ca^2+^ bath and then exposed to UV radiation to induce PEG-fibrinogen cross-linking. Before in vivo implantation, the alginate shell was removed. Bioprinted construct promoted longitudinal orientation of C2C12 cells and formation of highly aligned long-range multinucleated myotubes, with abundant and functional expression of myosin heavy chain and laminin.

To form structures of more complex architecture, one can generate multicompartment muscle-like fibers^212^ or sheets^281^ built from multiple parallel muscle cell-laden units. Zhao et al.^212^ used a multichannel microfluidic chip to produce alginate microfibers with the longitudinal grooves filled with GelMa (Figure 28E). The generation of such grooved microfibers relied on the in situ gelation of high and low concentrations of sodium alginate, which resulted in contracted or expanded longitudinal regions after solidification. To test the effect of the grooved morphology on muscle cell alignment and orientation, the C2C12 cells were seeded on the homogeneous (GelMa-alginate) and heterogeneous (GelMa-alginate and alginate alone) microfibers. Cells cultured on both types of microfibers exhibited similar viability. In the case of homogeneous microfibers, C2C12 cells grew on the whole surface of the fiber, including the grooves and convex part, while on the heterogeneous microfibers, the cells grew only in the grooves. However, while the orientation of myoblasts on the flat GelMa-alginate slabs was random, showing no obvious alignment of cells, the C2C12 cells seeded on the heterogeneous microfibers were strictly aligned and exhibited more elongated morphology.

Control over cellular arrangement was also demonstrated by Smandari et al.^400^ who used a specially designed premixing channel, supplying an intertwind alginate/GelMa stream, to formulate hydrogel microfibers with inner microstructure consisting of highly aligned elongated pores. The structure has been shown to dramatically enhance the alignment and metabolic activity of C2C12 myoblasts as compared to those cultured in microfibers formulated using a homogeneous bioink of the same composition (fully mixed alginate and GelMa).

Overall, despite wide spectrum of skeletal muscle-like microtissues of high biological relevance, the number of microfluidics-assisted structures addressing human skeletal muscle tissue is limited. The available studies are mostly based on the use of C2C12 murine muscle cells, which are easy to propagate and grow more rapidly when compared to primary muscle cells. Hence, the emphasis should be put on the design of human skeletal microtissues, which may require different strategies based on human primary muscle cell culture and/or generation of muscle cell lineages from multipotent stem cells. A very recent example comes from the work of Costantini et al.,^46^ who developed wet-spun muscle fibers based on primary mouse mesoangioblasts. Implantation in vivo in immunocompetent mouse resulted in impressive up to 80% restoration in tibialis anterior muscle functionality, as revealed by electrophysiological scoring of the muscle strength.

#### 5.2.6 Bone and Cartilage

Bone and cartilage are key components of the skeletal system, providing the major structure of the body of vertebrates and conferring protection and support of soft tissues. The bone and cartilage tissue engineering constitute a wide spectrum of applications ranging from bone and cartilage regeneration, reconstructive surgeries, to arthritis treatments and provide a promising alternative to conventional bone substitution strategies like autografts, allografts, or xenografts. In general, microfluidic-based strategies aiming to create functional bone and cartilage microtissue are based on incorporation of living cells into matrices or hydrogel scaffolds of highly tunable topology and architecture, providing a structural support for cell adhesion. This can be achieved via either encapsulation of cells with osteogenic and chondrogenic properties into biocompatible and biodegradable hydrogel capsules,^205^ fibers,^192,255,272,398,401^ or other types of structures of desired structural/mechanical properties^128,231,273^ or via fabrication of microfluidics-based porous hydrogel materials of required morphology (porosity/pore size) aiming to provide a permissive environment for cell proliferation and growth.^66,127,215^ The majority of the approaches is based on the use of mesenchymal stem cells and bone marrow stem cells due to their high accessibility and well-established differentiation protocols. To support bone regeneration, osteogenic cells are often coseeded with endothelial cells, providing local vascular network aiming to deliver oxygen and nourishment to the engrafted bone tissue, thus enhancing cell survival.^128,205,255,402^ Cartilage, unlike bones, is avascular.

Alginate capsules of spiral topology constitute an interesting example of microstructures dedicated for generation of vascularized bone microtissues.^205^ The spiral arrangement of originally parallel bioinks increases the surface of heterotypic cell–cell contacts, here HUVECs and hMSCs, as compared to more conventional Janus beads. The internal helical topology also enhances vascularization of the bone microtissue and promotes osteogenic cell survival and proliferation.

**Figure 29:**
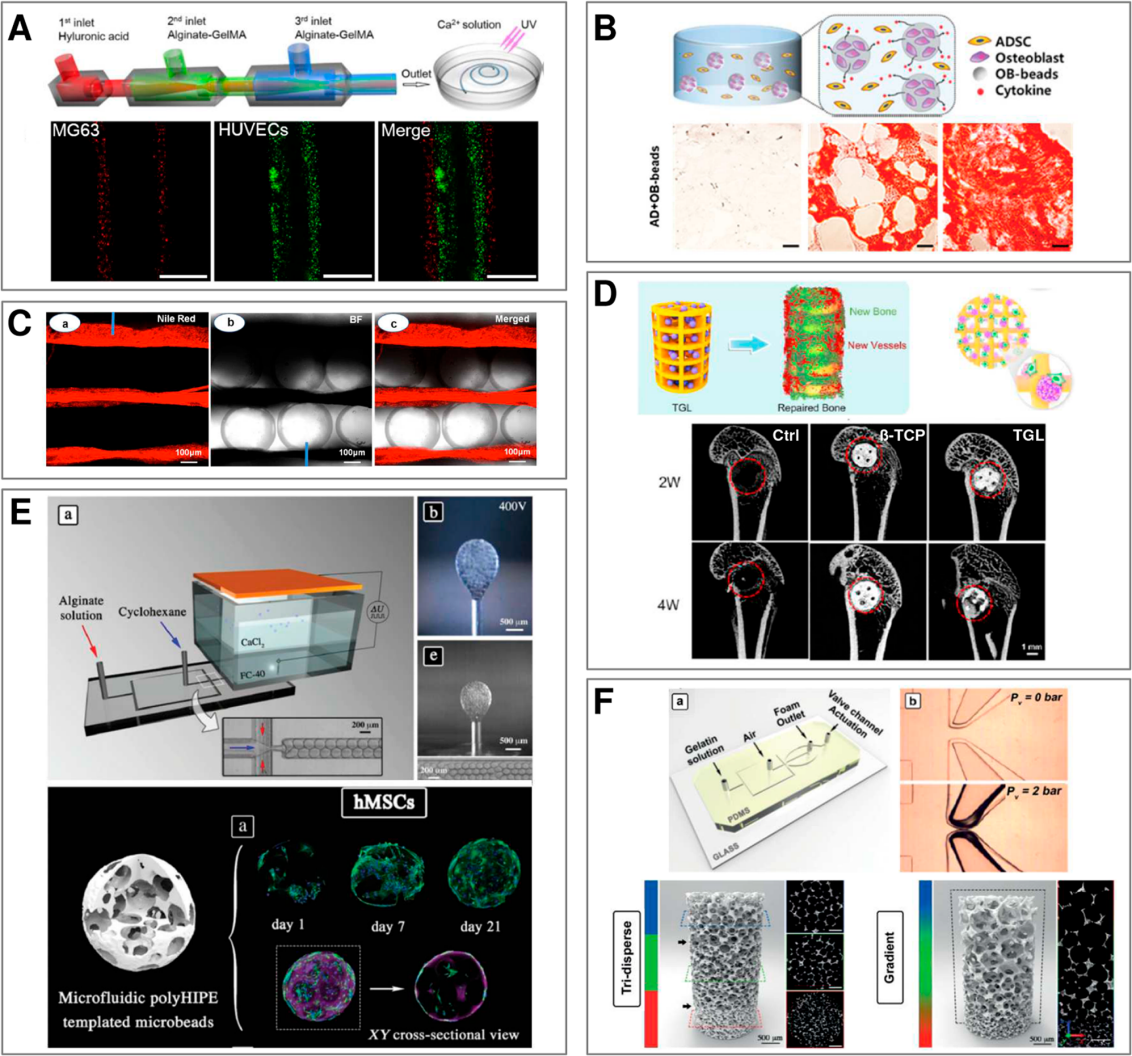
Microfluidics-assisted bone and cartilage microtissues. (A) Scheme illustrating production process of the double-layer hollow microfibers for vascularized bone engineering (top panel). Fluorescent images of microfibers with MG63 cells stained with CM-DIL (red) encapsulated in the outer layer and HUVECs stained with CM-FDA (green) encapsulated in the middle layer to showing cell distributions in the microfibers (bottom panel). Scale bars 500 μm. Adapted with permission from ref (255). Copyright 2016 Elsevier Ltd. (B) Schematic illustration of a 3D indirect coculture system for ADSC differentiation into osteogenic lineage by the paracrine effect of osteoblasts encapsulated in microbeads (top panel). Calcium deposition analysis by Alizarin red S staining of cryosectioned scaffolds. Scale bar 200 μm (bottom panel). Adapted with permission from ref (231). Copyright 2020 The Royal Society of Chemistry. (C) The cross section of the nanomicro alternating multilayer scaffold generated by alternately repeating the electrospinning of the microfluidic processes. The image shows three layers of electrospinning nanofiber membranes made of PCL nanofibers stained with Nile red (red) and two layers of alginate beads generated with a glass microcapillary microfluidic device. Adapted with permission from ref (273). Copyright 2015 Elsevier BV. (D) Schematic diagram of composite scaffold with incorporated GelMA microspheres with liposome (GML) into β-TCP scaffold (TGL) and its biological affects in bone repair process (top panel). Micro CT images of bone defects in rats after 2 and 4 weeks (W) after implantation of the scaffold. The red circle is the site of bone defect constructed by an electric drill (middle panel). 3D reconstruction of micro-CT images of the scaffolds showing their regenerative effects in vivo after 4 weeks (bottom panel). Adapted with permission from ref (128). Copyright 2020 The Authors. Published by Elsevier BV on behalf of KeAi Communications Co. Ltd. (E) Schematics of the device used for the manufacturing of porous microbeads and lateral light microscopy images of the 30G needle during the production of a “droplet of emulsion” at 400 V with precisely controlled microbead pore sizes (top panel). Maximum intensity projection microscopy images of hMSCs cells on porous microbeads templated from the microfluidic. Samples are stained against F-actin (green) and nuclei (blue). Autofluorescence of genipin-cross-linked chitosan in the red/far-red spectral range was exploited to reveal scaffold structure (in purple) (bottom panel). Adapted with permission from ref (66). Copyright 2018 Wiley-VCH. (F) Schematic of the valve-based flow focusing chip and optical micrographs of the vFF device for Pv = 0 and 2 bar, showing the squeezing of the orifice (top panel). 3D reconstructions of layered and graded porous materials obtained from mCT scans and horizontal (for tridisperse materials) and vertical (for the graded material) cross-sectional images (scale bars = 400 mm), showing the varying pore size along the *z*-axis (bottom panel). Adapted with permission from ref (127). Copyright 2019 Wiley-VCH.

Microfibers employed in bone tissue engineering most frequently rely on the core–shell topology,^192,255,272^ examples ranging from vascularized bone models^255^ to extrusion-bioprinted constructs for cartilage restoration.^192^ Zuo et al.^255^ designed a double-coaxial capillary microfluidic device to mimic unique concentric double-ring confirmation of the osteon, the basic functional bone unit (Figure 29A). Fabricated double-layer hollow microfibers with osteon-like structure comprised of HUVECs in the middle layer imitating vascular vessels and human osteoblast-like cells (MG63) in the outer layer representing the bone tissue. The middle and outer layer of the microfibers were composed of alginate–GelMa composite hydrogel, whereas the inner layer consisted of hyaluronic acid. The incorporation of GelMa into two outer layers of the microfibers allowed lowering of the concentration of alginate, resulting in enhanced biocompatibility of the hydrogel, better swelling characteristics, and mechanical properties roughly unaltered, as compared to the pure alginate structures. GelMa–alginate composite hydrogels were also successfully used to generate cell-laden bioinks for cartilage regeneration.^192^ In this case, to enhance viability and chondrogenic differentiation of BM-MSCs, the composite hydrogel was additionally supplemented with biopolymers present in the native cartilage ECM, including chondroitin sulfate amino ethyl and hyaluronic acid. Alginate shell served only as the templating agent to form stable fibers during 3D printing. Three photocurable bioink solutions with increasing degree of biomimicry: (i) GelMA, (ii) GelMA+CS-AEMA, and (iii) GelMA+CS-AEMA+HAMA were loaded with bone marrow-derived human mesenchymal stem cells and neocartilage production was evaluated.

High dimensionality and complex architecture of bones and cartilage require production of preshaped structures of desired topological and morphological properties, allowing for easy and immediate implantation of the generated microtissues into the site of injury. Such requirements call for mesoscopic 3D structures, which currently constitute the largest group of bone and cartilage-mimicking microtissues with demonstrated in vivo applications. Two major strategies to fabricate bone and cartilage-mimicking 3D topological microtissues can be distinguished.

The first group is based on the microfluidic generation of hydrogel beads subsequently embedded or injected into preshaped scaffolds or matrices, providing structural support and architecture of the generated microtissues.^128,231,273,362^ Kim and co-workers^231^ developed a 3D indirect coculture system based on collagen hydrogel containing alginate osteoblast-encapsulated microbeads embedded within bulk collagen matrix with adipose-derived mesenchymal stem cells (ADSCs) (Figure 29B). Compartmentalization of the scaffold and according spatial cell segregation was exploited to study the paracrine effect between ADSCs and osteoblasts. The authors demonstrated that stem cells cultured in 3D collagen hydrogels with osteoblast-loaded alginate microbeads exhibited osteogenic differentiation with increased osteogenic functions in vitro and in vivo when compared to ADSCs-laden hydrogel scaffolds alone or more conventional scaffolds encapsulating only growth factors. The paracrine effect between ADSCs and osteoblasts led to higher expression of osteogenesis markers including alkaline phosphatase, collagen type I, and osteocalcin and induced new bone tissue formation in vivo in the rat calvarial defect model. Another approach to arrange hydrogel beads into stable 3D bone-mimicking structure was presented by Ding et al.^273^ Bone marrow mesenchymal stem cells (rBMSCs) and bone morphogenetic protein-2 (BMP-2) laden calcium alginate microbeads were incorporated into electrospun polymer nanofibers to generate multilayer scaffolds, which were subsequently transplanted into the dorsal surface of the Sprague–Dawley rats to assess the osteogenic differentiation and osteogenesis (Figure 29C). Histological and immunohistochemical studies revealed that rBMSCs and BMP-2 loaded multilayer scaffolds efficiently induced ectopic bone formation in vivo.

Alternative approach to bone regeneration relies on the use of bioinks consisting of osteogenic cell-loaded microcapsules suspended in the external hydrogel.^398,401^ Chai et al.^401^ studied the application of 3D cell-laden constructs composed of cell-laden core–shell capsules with collagen core and alginate shell and methacrylated silk fibroin (SilMA) and GelMa hydrogel composite in rat model of skull defect. The authors showed that 2 weeks after implantation, the cell-laden Microgel 15% SilMA/GelMa constructs placed on the skull defects of rats showed better bone repair efficiency than 15% SilMA/GelMa constructs alone, mostly due to the higher cell viability than that of the 15%SilMA/GelMa group.

In yet another approach, the cells were seeded outside rather than inside the beads. The interbead space was used for cell seeding, with the outer bead surface providing the substrate for cell adhesion. Han et al.^128^ combined microgels with bioceramic scaffold for vascularized bone tissue engineering. GelMa beads, encapsulating deferoxamine (DFO)-loaded liposomes, were injected into the 3D printed ceramic scaffold to construct an internally vascularized bioceramic 3D construct (Figure 29D). Encapsulation of DFO-loaded liposomes into hydrogel microspheres allowed for controlled release of the DFO, resulting in increased angiogenesis of HUVECs and differentiation of BMSCs into the osteoblasts in vitro. At the same time, the ceramic scaffold served as a structural support for new bone tissue formation. In vivo, the composite scaffold increased the expression of vascularization and osteogenic related proteins like Hif1-α, CD31, OPN, and OCN in the rat femoral defect model, significantly reducing the time of bone repair.

The second group of 3D bone and cartilage-mimicking microtissues comprises porous structures of highly tailorable dimensions and architecture and ranges from 3D porous structures with monodisperse pores^215^ to monodisperse porous beads^66^ and functionally graded porous materials.^127^ Costantini and co-workers^66,127,215^ presented a comprehensive set of studies aiming to characterize the architecture and functionality of porous scaffolds templated from microfluidic emulsions or foams. First, the authors showed that sponge-like alginate scaffolds prepared by traditional vs microfluidic foam templating, although presenting a similar architecture, remarkably differ in terms of microstructural homogeneity.^215^ The microfluidic scaffolds exhibited a higher degree of uniformity, which correlated with better biological performance and superior capacity of cell seeding. Second, bone and cartilage-progenitor cells were seeded onto microfluidic-templated porous microbeads. The cells infiltrated and uniformly colonized the beads after 3 weeks of culture^66^ (Figure 29E). Last but not least, the authors presented a novel approach toward the fabrication of biocompatible macroporous materials in which pore sizes varied throughout the structure.^127^ A microfluidic flow-focusing junction with on-demand adjustable size of the orifice was used to generate porous structures with continuously varying pore size, with pore diameters in the range of 80–800 μm (Figure 29F). Moreover, the chip was used to supply a printhead of an extrusion 3D printer and allowed to print porous constructs of predefined 3D geometry and controlled, spatially varying internal porous architecture mimicking the bone structure. Altogether, the studies demonstrated great potential of microfluidic-assisted porous scaffolds in bone and cartilage tissue engineering.

In conclusion, compartmentalized microgels show a great potential in bone and cartilage restoration therapies. Microfluidic-based strategies are highly effective in promoting bone formation in vivo in animal models and facilitate repair of injured bone tissue. Moreover, survival of the engrafted bone microtissue can be enhanced by incorporation of the endothelial cells in the model or/and by providing a permissive environment for their growth. This may be of particular importance upon translation into clinics, where, in larger tissue constructs, microencapsulation helps to avoid tissue necrosis. High accessibility of patient-derived BM-MSCs and well-established protocols of their differentiation inside hydrogel capsules make microfluidic-based bone and cartilage microtissues a promising solution for patients suffering from volumetric loss of bone or cartilage.

#### 5.2.7 Vasculature

The vascular system consists of complex network of arteries, veins, and capillaries that carry blood and lymph through the body and provide nourishment, help in fighting diseases, and maintain homeostasis. The hierarchical architecture of the vascular system is reflected by morphological and functional diversity of the blood vessels. For example, the walls of arteries are thick multilayered structures comprising squamous epithelium, connective tissue, and smooth muscle cells, whereas the walls of capillaries constitute only a single layer of endothelial cells. To deliver oxygen and nourishment to every single cell in the body, the arteries branch into smaller arterioles and then into the smallest blood vessels, the capillaries. The walls of capillaries are permeable to oxygen and carbon dioxide, allowing for local gas exchange. Hence, nonvascularized tissues or tissues with impaired vascularization, for example, cellular grafts lacking endothelial-cell permissive environment or organs after injury will become necrotic and nonfunctional.

The diversity of the structure and function of blood vessels requires a broad range of microfluidic systems able to address topological differences between arteries, veins, and capillaries. Due to the high structural linearity of the blood vessels in vivo, the majority of the microfluidic-based approaches aiming to recapitulate vascular structures is based on generation of microfibers of various topology and cellular composition^45,67,80,194,202,203,208,255,262,272,277,402^ and 3D hydrogel scaffolds with bicontinuous topologies or channel-like architectures.^45,115,131,272^ The majority of the models relies on the use of human umbilical vein endothelial cells (HUVECs),^45,67,80,115,131,194,202,203,205,208,255,262,277^ which are easily accessible and can be propagated in culture for relatively long time. Hence, unlike in the case of cardiac and neural microfluidic-based tissue models, in vascularized tissue models, the use of stem cells is not the only viable option and indeed most studies rely on the use of HUVECs.

**Figure 30:**
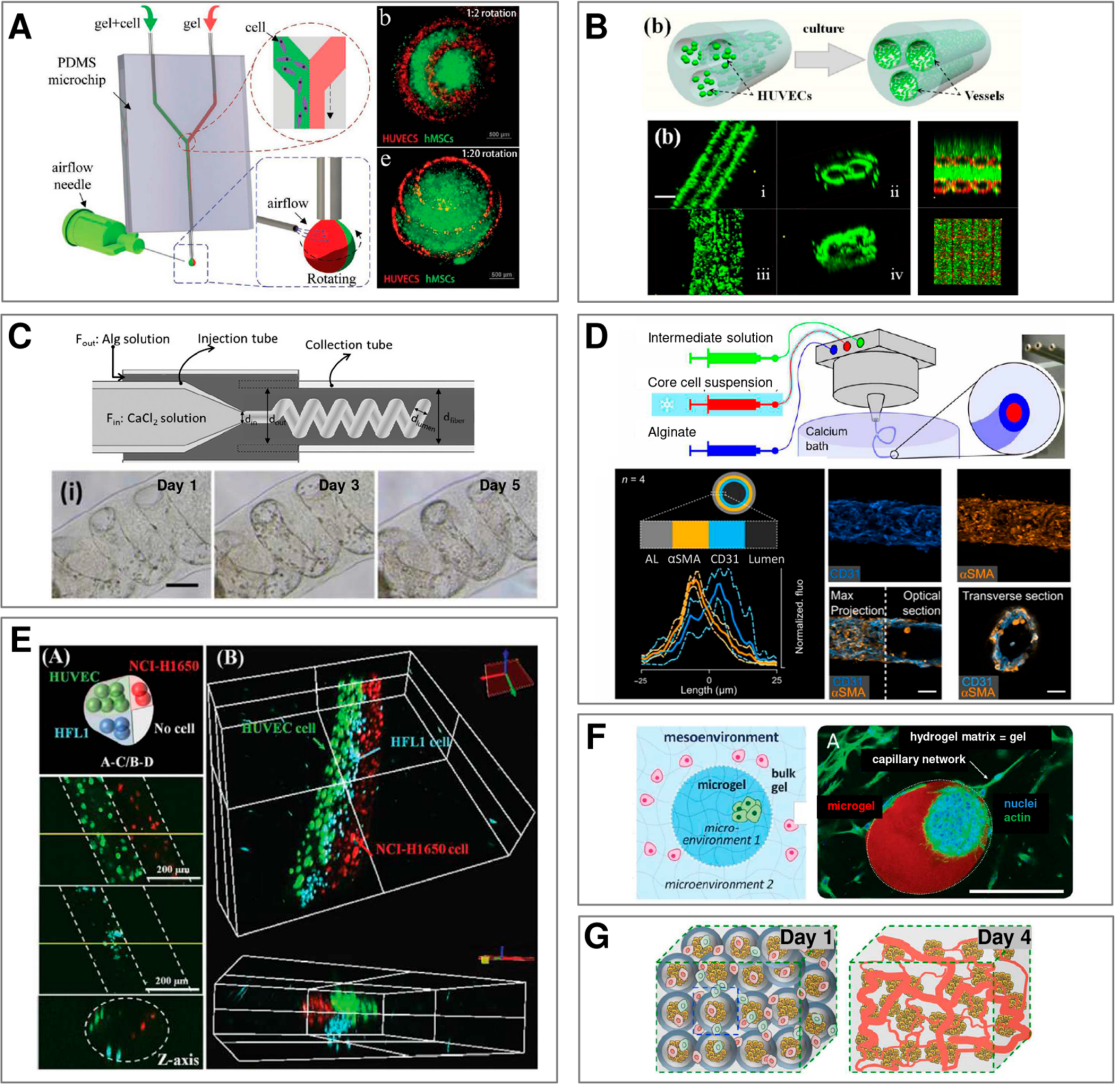
Microfluidics-assisted vascular microtissues. (A) Schematic representation depicting the process of fabrication of 3D spiral-based cell-laden spheroids and an image of vascularized organoids construction through the coculture of HUVECS and HMSCs in spiral-based microspheroids. Adapted with permission from ref (205). Copyright 2018 Wiley-VCH. (B) Generation of biomimetic vessels by using the multihollow microfibers, schematic illustration (top panel). Biomimic vessel with HUVECs in the hollow cores of a GelMA microfiber with different numbers of cell tubes and layer-by-layer architecture of 3D structures made by stacking hollow microfibers (bottom panel). Scale bars 200 μm. Adapted with permission from ref (202). Copyright 2016 American Chemical Society. (C) Schematic illustration of the microfluidic device for fabricating helical hydrogel microfibers (top panel). Proliferation and tubular structure formation of HUVECs in hollow alginate–collagen microfibers during the course of culture (bottom panel). Scale bar 200 μm. Adapted with permission from ref (208). Copyright 2019 Wiley-VCH. (D) Drawing of the microfluidic platform and picture of the coextrusion device for multilayer vessels generation (top panel). Self-organization of generated structures into artificial mature blood vessels. Fluorescence intensity profiles and immunostainings of SMA (muscle cells) and CD31 (endothelial cells) in formed multilayered vesseloids. Scale bars 100 μm. Adapted with permission from ref (262). Copyright 2019 The Authors. (E) Multicomponent Janus microfiber for coculture of three types of cells (NCI-H1650, HUVECs, and HFL1 cells). Adapted with permission from ref (80). Copyright 2020 The Authors. Published by Wiley-VCH. (F) Design of microgel-in-gel system to recapitulate different cellular mesoenvironments and visualization of the 3D in vitro microgel-in-gel vascularized prostate cancer model by immunostaining. Actin filaments stained with phalloidin, Hoechst staining of nuclei, and microgel labeled with atto 610. Scale bar 250 μm. Adapted with permission from ref (131). Copyright 2020 The Royal Society of Chemistry. (G) A schematic illustration of 3D vascularized human tumors models. Cancer cells are encapsulated in the core of microcapsules with a hydrogel shell for miniaturized 3D culture and used as the building blocks for assembling with endothelial cells and other stromal cells to create macroscale 3D vascularized tumor. Adapted with permission from ref (115). Copyright 2017 American Chemical Society.

The application of “0D” structures for culturing of vascularized microtissues is relatively uncommon and includes mostly drug-loaded capsules promoting angiogenesis^348^ in the external microenvironment as well as coculture systems based on compartmentalized microgels.^205^ An interesting example of the latter one has been presented by Zhao et al.^205^ An airflow assisted 3D bioprinting method was used to generate human multicellular organoids of spirally vascularized ossification (Figure 30A). Precisely, two (or more) streams of cell laden Na-alginate solutions were coextruded into a hanging droplet subsequently subjected to an external precisely directed airflow. This led to droplet spinning and resulted in spiral-like arrangement of the hydrogel streams entering the droplet. The coflowing streams were generated using a Y- or ψ-shaped microfluidic junction, which allowed for coextrusion of up to three parallel bioinks from a single nozzle and generation of capsules with heterotypic cell contacts. The spinning of the droplets allowed for generation of beads with complex internal topology, including helices and rose- or saddle-like patterns. In particular, the spiral-based vascularized spheroids were generated using two distinct streams carrying each either HUVECs or hMSCs. The HUVECs were distributed either at the surface or inside the spheroids in controlled manner, which allowed to guide the formation of the capillary-like networks. Cell viability at the level of 80% was demonstrated after 10 days in culture. In addition, the coextruded hMSCs were differentiated into an osteogenic cell line and showed formation of alkaline phosphatase and Alizarin red S-positive osteogenic nodules surrounded by the capillary-like network.

Despite the interesting examples of generation of internally compartmentalized microcapsules, the development of the vessel-like structures relied mostly on the application of the microfibers. The interest in microfibers is due to their natural vessel-like morphology. The studied systems ranged from simple monoculture linear systems mimicking single capillaries^45,202,208,272,402^ through core–shell artery-like structures^262^ to other compartmentalized microfibers of complex internal topology, allowing coculture studies and typically dedicated to bioprinting of vascularized constructs.^80,194,203,255,271,277^ Capillary-like structures were generated either via encapsulation of the endothelial cells inside the core of the core–shell hydrogel fibers^45,272^ or by seeding the cells inside the previously generated hollow microfibers. In the latter case, the cells typically tend to attach to the inner wall of the hollow fibers and align to form lumen-like structures.^67,202,208,272,402^ One meter long endothelial cell fibers with HUVEC-laden ECM-filled core and alginate shell were successfully generated by Onoe et al.^45^ The authors demonstrated that HUVECs spontaneously assembled into lumenous tubular structures. Recently, Wang and co-workers^402^ demonstrated that such endothelial cell laden microfibers can be further implanted in vivo. Rat umbilical vein endothelial cell-laden hollow fibers made from alginate–collagen composite hydrogels were implanted into a GelMa scaffold in a rat critical-sized calvarial model. Engrafted endothelialized tubes locally enhanced the secretion of growth factors, facilitating osteogenesis, and thus promoting bone regeneration. The authors suggested that, in the future, the technology could be adopted in engineering of other tissues to promote in situ tissue repair/regeneration.

From the point of view of biomimetics, it is known that the endothelial cells (as well as other cell types) tend to develop the most physiological characteristics when suspended in the ECM-like matrix. In the case of core–shell microfibers with alginate shell, this was achieved via introducing another ECM-like hydrogel, typically GelMA or collagen, to the core. Indeed, such modifications were shown to enhance endothelial cell survival and proliferation.^202,208,272,402^ It is noteworthy that the morphology of hollow microfibers can be further tuned, e.g., via introducing several parallel cores instead of a single core by using various types of microfluidic nozzles.^67,202,208,272^ For instance, Cheng and co-workers^202^ designed a multibarrel capillary microfluidic device, generating laminar coflow of multiple alginate streams to fabricate alginate-GelMa microfibers with one, two or three hollow cores, subsequently seeded with endothelial cells (Figure 30B). The authors showed that after 5 days of culture HUVECs densely spread on the inner surface of the hollow cores into a monolayer, hence forming tube-like tissue closely resembling the morphology of blood vessels in the human body. It was subsequently demonstrated that such tube-like structures can be arranged, via layer-by-layer deposition, into a complex 3D grid with well-preserved core–shell hollow topology of each endothelial tube-like unit.

The morphology of blood vessels is highly diversified in vivo, including not only straight and curved channels but also helical tubes. Such helical vessels are responsible for creating swirling blood flow (so-called helical blood flow) in certain arteries of the human body, e.g., in endometrial spiral arterioles or intestinal villi spiral arterioles.^403^ Interestingly, some studies suggested protective properties of swirling blood flow in preventing cardiovascular diseases such as atherosclerosis^404,405^ as well as enhanced oxygen transportation from blood to the arterial wall caused by the helical vessel morphology.^406^ Such morphology was addressed by Jia and co-workers.^208^ The hollow helical microfibers composed of alginate–GelMa complexes were generated using coaxial capillaries and seeded with HUVECs (Figure 30C). The helical morphology was achieved via lowering the speed of flow of the external coflowing phase below the speed of flow of the inner fiber phase. The backward viscous drag acting on the fiber caused its steady coiling into a regular helix. The exact morphology of the helices was tuned either via changing the flow rates or via modifying the geometry of microfluidic device (diameters of the capillaries). For example, lowering (or increasing) the speed of the inner flow while keeping the outer flow constant resulted in generation of helices with larger (or smaller) pitch. By tuning the microfluidic setup several types of complex perfusable helical structures, including multilayer helical microfibers and superhelical hollow microfibers (helix in helix) were generated. However, no detailed functional studies of the endothelial cell-seeded hollow helical microfibers were performed.

The vessel-like microfibers described so far consisted of only endothelial cells and thus formed, strictly speaking, endothelial tubes.^45,67,202,208,272,402^ These endothelial tubular structures successfully mimic blood capillaries but do not fully reproduce the structural complexity of higher-order vessels, both in terms of cellular composition and functional properties. While ensuring perfusability, the simple endothelial tubes fail to recapitulate the elasticity and contractility, e.g., of the arterial blood vessels that are required to accommodate the pulsatile blood flows. More precisely, in physiological conditions, arterioles exhibit an organized histological shell-in-shell cross-sectional structure comprising a layer of smooth muscle cells enveloping a layer of endothelial cells around a lumen. The presence of the smooth muscle cells allows for vessel contraction, which is critical for maintaining of the arterial blood flow. Andrique et al.^262^ proposed a one-step strategy to generate mature functional blood vessel-like structures of well-defined layered cellular composition using a microfluidic coextrusion device where three solutions were injected simultaneously by a computer-controlled pump (Figure 30D). The shell phase consisting of alginate solution was injected into the outer channel, the core phase composed of smooth muscle cells and HUVECs suspended in ECM solution was injected into the inner channel, while an intermediate phase consisting of d-sorbitol solution was injected into the middle channel. The role of the intermediate phase was the separation of the shell and core streams until they reached the external calcium bath, which allowed avoidance of untimely alginate cross-linking, otherwise possible due to cellular calcium release. The endothelial cells and smooth muscle cells, initially intermixed in the core, spontaneously self-assembled into the layered configuration at the inner wall of the alginate shell. The cellular self-organization resulted in the correct (physiological) cell configuration with the lumen of the vessel surrounded directly by HUVECs and with the smooth muscle cells forming the outermost cell layer directly attached to the inner wall of the protective alginate shell. The vessel-like structures reached homeostasis within 1 day and exhibited properties of functional vessels, including, besides the double layer histological structure, also perfusability and contractility in response to vasoconstrictor agents. Nevertheless, generation of microfluidic-based hierarchical microvessels still remains an open challenge.

A separate group of linear structures consists of coculture microfibers of complex topology including multishell fibers,^255^ multicore fibers,^194^ and Janus structures,^80,203,271,277^ from which some of them are dedicated to bioprinting of vascularized tissues.^203,277^ Here, the compartmentalization allows for generation of microfibers of different cellular composition by seeding endothelial cells and tissue specific cells in parallel compartments of the fiber (Figure 30E). Examples include double-shell hollow alginate-GelMa microfibers seeded with HUVEC cells and MG63 human osteosarcoma cells,^80,255^ triple-Janus alginate microfibers loaded with HUVECs, lung fibroblasts, and lung cancer cells^80^ (Figure 30E), lotus root-like alginate fibers with HUVECs cells encapsulated in the central core and hepatocarcinoma cells (HepG2) into the six-barrel core of the microfiber^194^ and two-compartment Janus fibers for cardiomyocyte and endothelial cell coculture.^203,277^ The latter structures were successfully used to restore vascularized cardiac tissue and are described in detail in section 5.2.4 of this review. The remaining structures mentioned above lacked functional validation.

Applications of vascularized microtissues are not limited to the coculture linear systems for vascularized tissue engineering^80,194,203,255,271,277,402^ but also have a great potential in design of 3D vascularized cancer models.^115,131,407,408^ Microgel-in-gel structures based on cell-laden spherical microgels (hydrogel microparticles) embedded in cell-laden bulk hydrogel matrices provided thoroughly tunable systems to study tumor-stroma cross-talk during the early stages of tumor angiogenesis (Figure 30F,G). Husman et al.,^131^ developed a prostate cancer model in which PC3 cancer cells were encapsulated in starPEG-heparin hydrogel beads to spontaneously assemble into tumor spheroids and after one-week preculture embedded into softer starPEG-heparin bulk hydrogels, together with suspended HUVECs and MSCs (Figure 30F). After 5 days, microgel-in-gel culture resulted in vascularized matrix-embedded prostate cancer spheroids. The established structure was characterized using immunocytochemistry, allowing for visualization of newly formed capillary network. Moreover, it was proposed that the 3D modular cancer microtissues can be used to create macroscale vascularized tumors for studying cancer progression both in vitro and in vivo and to test efficacy of various anticancer therapeutic agents. Agarwal and co-workers^115^ reported a bottom-up approach for creating 3D vascularized human breast cancer tumors. MCF-7 cancer cell-laden core–shell capsules with the liquid core comprising tumor cell suspension in ECM and with an alginate–collagen hybrid hydrogel shell were assembled together with HUVECs and human adipose-derived stem cells in bulk collagen hydrogel (Figure 30G). 3D vascularized tumor blocks were placed in the microfluidic perfusion device, allowing for vascular capillary network formation. Vascularized microtumors were then used in in vitro anticancer efficacy tests of free and nanoparticle-encapsulated doxorubicin, showing that such cultures may serve as a valuable model in drug discovery and to study the effect of tumor microenvironment on cancer progression and metastasis. Moreover, the authors showed that subcutaneous injection of tumor blocks into athymic nude mice results in formation of resultant tumors in vivo. In summary, the microgel-in-gel structures provide a versatile tool in microtissue engineering. The corresponding 3D modular cancer vascularized microtissues can become instrumental for studying various aspects of cancer biology, including invasion/metastasis and tumor interactions with the endothelium at high levels of precision both in vitro and in vivo.

In summary, the presented strategies, although highly successful, mostly aim at the development of simple endothelial cell tubes. Future efforts should focus on the construction of more complex vessels with a fully biomimetic multilayer structure incorporating several heterotypic cell types. Establishing protocols for generation of blood vessels of predefined diameter and more complex cellular composition could help to fabricate microfluidic-based hierarchical vasculatures in the future. Development of such vascular networks still remains one of the biggest challenges in tissue engineering. Nevertheless, microfluidic technologies have allowed significant progress in the development of new vascularized tissue models. Incorporation of vasculature in the engineered microtissues has been shown to improve the survival of the engineered cell-laden micro scaffolds and allowed for more accurate modeling of healthy and diseased human tissue. In particular, vascularized tumor models have a great potential to improve the accuracy and efficiency of anticancer drug screening and can serve as a powerful tool in studying the mechanisms of cancer biology.

#### 5.2.8 Stem Cells

The production of an engineered tissue in vitro requires a significant amount of cells to populate the hydrogel scaffolds. The use of the primary cells, taken from the patient, however successful, has several limitations such as (i) the necessity of performing invasive procedures to collect cells and (ii) the possibility that the cells do not survive the procedure. Therefore, stem cells, which can renew themselves through cell division, and under certain conditions differentiate into specialized cell types, offer a great alternative and currently play a major role in regeneration of tissues and restoration of tissue functions. Based on their differentiation potential, stem cells can be divided into two categories: pluripotent and multipotent stem cells. Pluripotent stem cells include embryonic stem cells (ESCs) and induced pluripotent stem cells (iPSCs). Because ECSs need to be isolated from the inner cell mass of the blastocyst during embryological development, their use is controversial and usually limited to modeling of animal tissues. The iPSCs, on the contrary, can be generated by reprogramming adult fibroblasts which makes them more accessible for generation of human-origin microtissues.^390^ Both, ESCs and iPSCs, due to their pluripotent character and the associated high risk of spontaneous and unspecific differentiation, for example, into cancerogenic cells,^409^ require well-defined and highly controllable environment during differentiation process. On the other hand, multipotent stem cells, which can be isolated from numerous adult tissues, including bone marrow, peripheral blood, adipose tissues, or nervous tissues have a larger capacity to differentiate into a limited number of cell types as compared to ESCs. For example, mesenchymal stem cells (MSCs) which reside in the bone marrow can differentiate into bone (osteoblasts),^410^ muscle (myoblasts),^411^ fat (adipocytes),^410^ and cartilage (chondrocytes)^410^ cells, while neural stem cells (NSCs) either give rise to the support cells in the nervous system (astrocytes and oligodendrocytes) or to neurons.^412^

Compartmentalized hydrogel microstructures have been demonstrated to provide a great potential in culture and differentiation of stem cells. The use of microfluidics allows generation of structures of predefined topology and tightly regulated microenvironment, which is critical for propagation of stem cells, maintenance of their pluripotency, and proper differentiation. Moreover, because the differences in spheroid size may influence the stem cell behavior and differentiation potential,^313^ the reproducibility, e.g., monodispersity, of capsules (or other types of microstructures) generated using microfluidics is of particular importance. Last but not least, scalability of the process allows for robust and large-scale production of stem-cell derived microtissues, thus fulfilling the crucial requirement of tissue restoration therapies.

Stem cells were successfully encapsulated and cultured in microfluidics-assisted topological microgels, resulting in a wide range of biomimetic microtissues including cardiac,^107,111,187,205^ nervous,^108,114^ muscle,^110,113^ liver,^112^ and bone or cartilage tissues.^66,192,205,215,231,273^ Such structures were shown to have a great potential in in vivo tissue restoration.^47,231,273^ Because the tissue-specific types of microtissues have been already thoroughly discussed in this review (see the previous sections 5.2.1–5.2.7), they will not be included in this chapter. Here, we focus on microfluidic-generated structures dedicated solely for propagation of stem cell culture and stem cell differentiation into target cell lineages.

**Figure 31:**
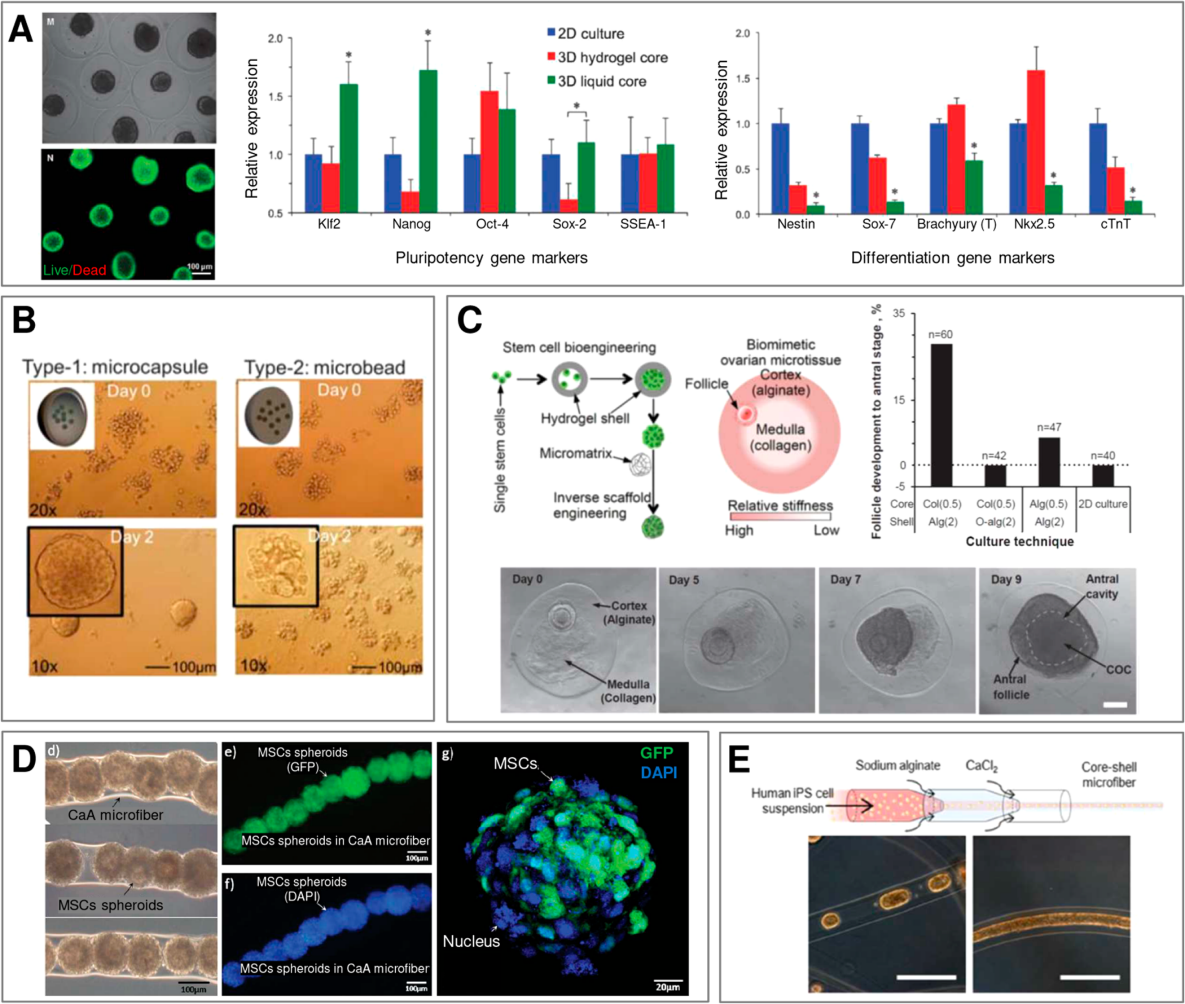
Microfluidics-assisted stem-cell spheroids. (A) Core–shell capsules with EC encapsulated in the liquid core; bright field image and live/dead staining (left panel). Pluripotency of ES cells obtained under three different culture conditions at the gene level. qRT-PCR data of pluripotent genes showing that, on average, the aggregated ES cells in the miniaturized 3D liquid core have significantly higher expression of Klf2 and Nanog compared to the cells under 2D culture and significantly higher expression of Klf2, Nanog, and Sox-2 compared to the cells cultured in the 3D hydrogel core (middle panel) and qRT-PCR data of typical germ layer markers and cardiac markers showing that ES cells from the miniaturized 3D liquid core have significantly lower expression of Nestin (ectoderm), Sox-7 (endoderm), brachyury (or T, mesoderm), Nkx2.5 (early stage cardiac marker), and cTnT (late stage cardiac marker) (right panel). Adapted with permission from ref (111). Copyright 2014 The Royal Society of Chemistry. (B) The formation of EBs by P19 cells in core–shell microcapsule (type 1) and microbead (type 2). Adapted with permission from ref (306). Copyright 2011 The Royal Society of Chemistry. (C) Schematic illustration of generation of biomimetic core–shell capsules and a scheme of biomimetic ovarian microtissue, showing stiffness gradient in the capsule (left panel). In vitro development of early secondary preantral follicles of deer mice under miniaturized 3D culture in microtissue. Typical micrographs showing the development to antral stage over 9 days of an early secondary preantral follicles in the collagen core of biomimetic ovarian microtissue with an alginate (nonoxidized) shell and quantitative analysis, illustrating the effect of the core (0.5% collagen or alginate) and shell (2% oxidized or nonoxidizedalginate) materials for making the ovarian microtissue on the development of early secondary preantral follicle into antral stage together with that from 2D culture. Col(0.5), 0.5% collagen; Alg(2), 2% alginate; O-alg(2), 2% alginate with oxidization; Alg(0.5), 0.5% alginate. Scale bar 100 μm (right panel). Adapted with permission from refs (52,183, and 187). Copyright 2016 American Chemical Society and 2014 Elsevier Ltd. (D) Bright field and fluorescent images of alginate fibers with encapsulated mesenchymal stem cell (MSC) spheroids. Adapted with permission from ref (60). Copyright 2014 Wiley-VCH. (E) An illustration of the double coaxial laminar flow microfluidic device used for the formation of the human iPS cell-laden core–shell hydrogel microfiber and microscopic images showing the morphologies of human iPS cell aggregations in the core–shell microfiber culture and the suspension culture systems on days 4 and 8. Scale bar 500 μm. Adapted with permission from ref (106). Copyright 2017 The Author(s).

Compartmentalized cell-laden hydrogel capsules, due to their easily tailorable physicochemical parameters and scalability of the production process, constitute the most common approach in microfluidic-based 3D stem cell culture.^47,52,95,107−109,111,121,183,187,306,310,340^ Additionally, as the stem cells have a tendency to form aggregates/spheroids in their native environment, the spherical topology seems to be the most relevant from the biological point of view. The core–shell capsules with a solid alginate-based hydrogel shell and a liquid core have been successfully used to culture pluripotent and multipotent stem cells, including ESCs^47,107,111^ and adult stem cells like adipose-derived stem cells.^340^ The liquid or nearly liquid (soft-hydrogel) microenvironment of the core supports cell mobility and thus promotes cell aggregation, which in turn helps maintain cell pluripotency.^413^ The He group^107,111,187,365^ developed a core–shell microcapsule system mimicking the miniaturized 3D architecture of prehatching embryos with an aqueous liquid-like core containing embryonic cells and the hydrogel-shell mimicking zona pellucida. The capsules were generated using a microfluidic flow focusing device^107^ or coaxial electrospray technology.^111,365^ The latter technique allowed for generation of hundreds of thousands of murine embryonic cell-laden microcapsules of high viability in less than 1 h. The encapsulated cells self-assembled into 3D aggregates of a size up to a few hundred micrometers as they do in their native milieu in a prehatching embryo. To analyze the pluripotency of mESCs mRNA expression levels of common stemness genes (Klf2, Nanog, and Oct-4), and differentiation genes including Nestin for ectoderm, Sox-7 for endoderm, Brachyury (or T) for mesoderm, Nkx2.5 for early cardiac commitment, and cTnT for late cardiac differentiation were evaluated.^111^ Cells encapsulated in bioinspired capsules were shown to maintain higher expression of the stemness genes and lower expression of all the differentiation genes than cells in the conventional 3D and 2D culture methods (Figure 31A). This shows higher physiological relevance of liquid-core/solid-shell capsules for stem cell culture and underlines the fact that the nonphysiological culture conditions may lead to altered gene and protein expression. These results actually support the previous microscopic observations by Kim et al.,^306^ showing that embryonic carcinoma cells encapsulated inside the liquid core of the microcapsules aggregate more easily than those in the solid bead and more effectively form single embryonic body (EB) (Figure 31B). Moreover, Zhao et al. further demonstrated that when mESCs-laden bioinspired microcapsules were subjected to cardiac differentiation their capability to differentiate into cardiac cells was higher than that of the ES cells under the 2D culture or cultivated in conventional 3D microbeads of homogeneous alginate hydrogel.^111^

The impact of mechanical and biochemical heterogeneity of the core–shell microcapsules on the stem cell proliferation and development was also investigated. Particularly, the effect of addition of ECM proteins, such as collagen, to the liquid core was studied.^52,95,183^ Preantral ovarian follicles were encapsulated in core–shell capsules and addition of hydrogels of various mechanical properties to the core phase was tested. The authors showed that encapsulation of preantral follicles in 0,5% collagen core engulfed by an alginate shell most accurately mimicked the in vivo conditions of oocyte maturation and resulted in the formation of the highest number of the antral stage structures^52,95^ (Figure 31C). Changing of mechanical properties of the capsule via either increasing the collagen concentration or via using oxidized alginate to reduce the mechanical strength of the shell resulted in significant decrease of the number of generated antral follicles. This has confirmed that mechanical heterogeneity plays a key role in regulating follicle development and ovulation and that microfluidic-based topological structures may provide excellent means in the ovarian tissue engineering. In particular, the new technology could help to better understand the mechanisms associated with ovarian disorders and serve as an in vitro culture system to preserve and restore fertility in women.

Encapsulation of stem cells in core–shell capsules has been also demonstrated using all-aqueous systems.^340^ Instead of oil, the authors used aqueous PEG solution as the external phase and aqueous dextran solution as the droplet “shell” phase. The inner “core” phase was also aqueous and contained cellulose for increased viscosity, which served to prevent mixing of the core and the shell phases. The PEG-rich and dextran-rich phases spontaneously phase-separated, which led to formation of the PEG/dextran interface with small, yet finite, interfacial tension; this in turn allowed formation of droplets. However, the droplet pinch off had to be enforced via periodic pressure pulses, e.g., generated with a solenoid valve.^340^ This strategy of generation of stem cell-laden core–shell hydrogel capsules allowed elimination of the use of oil, thus preventing any potential cytotoxic effects. However, the important limiting factor of the technology was the droplet generation frequency, which in the mentioned study did not exceed 4 Hz.

Even though the hydrogel microcapsules constitute the largest group of microfluidics-based stem cell culture systems, several works also exploited the use of microfibers for this purpose.^60,106^ Yu et al.^60^ used a combination of droplet microfluidic technique with the wet-spinning process to generate hybrid fibers of unique bamboo-like architecture suitable for stem cell spheroid culture (Figure 31D). The hybrid microfibers were successfully used for long-term cultivation of MSCs spheroids, providing sufficient nutrient and oxygen exchange. Moreover, unique design of the microfluidic setup allowed for simultaneous incorporation of two different kinds of droplets into the fiber in a highly controllable manner, hence generating fibers encapsulating well-defined droplet patterns. This was achieved via synchronized operation of two independent T-junctions, each operated by a different pneumatic valve integrated on-chip and dispensing the dispersed phase in a controlled manner. Interestingly, Ikeda et al.^106^ demonstrated that stem cells may preserve their pluripotency inside of the core–shell microfibers. More precisely, the authors showed that iPSCs encapsulated and cultured within the collagen-rich core region form rod-like cell aggregates of high viability and with fixed diameter set roughly by the inner diameter of the fiber core (Figure 31E). The iPSCs exhibited high expansion rates and retained pluripotency, as demonstrated with mRNA expression analysis of the pluripotency-associated marker genes (including Oct 3/4 and Nanog). The expression levels were higher in cells cultured in human iPSC-laden core–shell microfibers then in cells cultured in conventional 2D systems.

In summary, microfluidics-based topological hydrogel microstructures offer a promising tool toward high-throughput cultivation, optimization, and differentiation of stem cells while preserving their pluripotency and allowing for precise control over the differentiation process.

#### 5.2.9 Cancer

In the healthy tissue, cells grow and divide to form new cells as the body needs them. In cancer tissue, the cells grow and reproduce uncontrollably. When cells become old or subject to damage, they die, and the new cells take their place. When this highly organized process is disturbed, abnormal or damaged cells start to proliferate in an uncontrolled way, resulting in cancer tissue formation. The cancerous cells can spread toward other parts of the body through the blood and lymph systems and invade surrounding healthy tissue and organs in the process known as metastasis. According to the recent reports, cancer remains a major public health problem worldwide and is the second leading cause of death in the United States.^414^ So far, based on the origin of tissue and molecular characteristic of the tumors, more than 200 different types of cancer have been reported and most of them require specific diagnostic tools and treatment. Most common types of cancer include respiratory system and gastrointestinal cancers as well as breast and prostate cancers.^414^

Microfluidics holds great promise in cancer diagnosis as well as in basic cancer biology research. Microfluidic-based cancer microtissues emerged as a powerful tool in drug efficacy screening,^184,204,206,269^ including personalized cancer treatments^20^ and allowing for incorporation of complex interactions to mimic in vivo tumor microenvironment and/or recapitulate tumor metastasis.^75,115,131,206,300,374,407,415,416^

Microfluidics-assisted generation of hydrogel microcapsules, due to its reproducibility and scalability, constitutes one of the most common approaches in anticancer drug screening. Compartmentalized topologies including core–shell capsules^75,184,188,191,204,407^ and Janus structures^75^ were shown to enhance native cancer cell properties, including their ability to proliferate and grow uncontrollably as well as to invade surrounding tissue or ECM. Yu and co-workers^184,204^ demonstrated that the composition and mechanical properties of the extracellular matrix, particularly its stiffness, can determine organization and behavior of MCF-7 breast tumor cells. MCF-7 cells were encapsulated inside core–shell capsules with an alginate shell and with a core comprising a mixture of alginate, collagen, and reconstituted basement membrane. The cells, residing in the core, proliferated more rapidly and formed larger spheroids as compared to the cells embedded in homogeneous alginate beads. Moreover, dose-dependent comparative studies of the response of the MCF-7 cell tumor spheroids and MCF-7 monolayer culture cells to two anticancer drugs, docetaxel and tamoxifen, revealed^204^ that the mechanical pressure exerted on the cells by the shell upon tumor development can affect drug response. The authors suggested that the core–shell beads could be used in the future to study the influence of the external pressure, tunable via changing the concentration of alginate in the shell, on the growing tumors.

Core–shell hydrogel capsules were also successfully used to study cancer cell biology and facilitated rapid identification of efficacious therapeutics against prostate cancer. An interesting example is the study by Rao and co-workers,^188^ who investigated the impact of the presence of cancer stem-like cells, responsible for cancer resistance, recurrence, and metastasis, on the behavior of microtumors. PC3 prostate cancer cells with high metastatic potential were used as model cells and cultured in the aqueous liquid core of alginate core–shell beads. Local liquid environment supported fast cell aggregation into spheroids, which the authors referred to as “prostaspheres”, highly enriched with cancer stem-like cell population. Cells encapsulated in the core–shell microbeads exhibited higher expression of main stem cells marker genes including Oct4, Nanog, and Klf4 as compared to prostaspheres aggregated and cultured at ultralow attachment plates. Accordingly, the encapsulated cells developed higher tumorigenicity. Moreover, the encapsulation of PC3 cells in the liquid cores accelerated the formation of prostaspheres, with spheroid formation time decreasing from 10 to 2 days. Such expedited culture is particularly desirable in the perspective of personalized drug screening.

**Figure 32:**
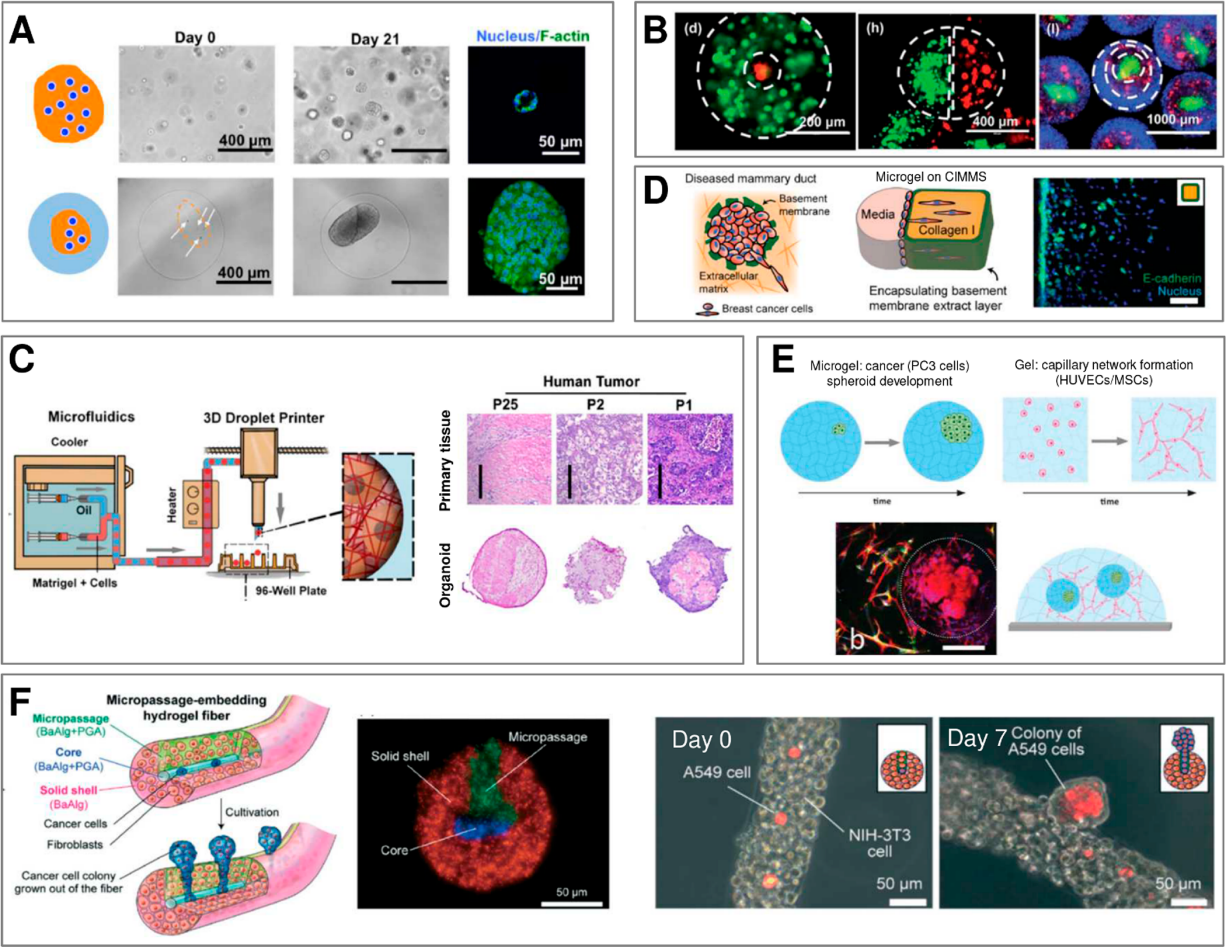
Microfluidics-assisted cancer microtissues. (A) Malignant transformation of mammary epithelium under confinement of core–shell microcapsules. MCF10A cells formed acini after being cultured in bulk Matrigel for 21 days (top panel), whereas MCF10A cells encapsulated in a Matrigel core within a core–shell microcapsule underwent uncontrolled growth (bottom panel). Adapted with permission from ref (191). Copyright 2019 Elsevier Ltd. (B) Double-layer, Janus, and triple layered hydrogel microparticles with MDA-MB-231 expressing GFP cells (green), normal human lung fibroblasts expressing RFP (red), and MCF-10A cells stained with Hoechst (blue) encapsulated in multicompartment hydrogel particles. Adapted with permission from ref (75). Copyright 2015 The Royal Society of Chemistry. (C) Sketch of the automated organoid platform illustrating an organoid fabrication module and an organoid printing module (top panel). Histological images of primary tumors and their organoids derived from human lung (P1), liver (P2), and gastric (P25) tumors. Scale bar 200 mm (bottom panel). Adapted with permission from ref (20). Copyright 2020 The Author(s). (D) Cartoon of diseased mammary duct and a core–shell microgel generated by using digital microfluidic microgel systems dedicated for cancer cell invasion studies (left and middle panel). A confocal image showing migration of MDA-MB-231 cells on day 4 after seeding a core–shell microgel. Cells were immunostained for nucleus (blue) and E-cadherin (green); inset cartoon illustrates the makeup of the microgel. Scale bar 100 μm (right panel). Adapted with permission from ref (374). Copyright 2020 The Authors. (E) 3D in vitro microgel-in-gel vascularized prostate cancer model. Scheme illustrating formation of cancer cell spheroids and capillary network formation of vascular endothelial cells (top panel). Illustration and a confocal image of microgel in gel system with PC3 cancer cell laden hydrogel beads embedded in external hydrogel with endothelial cells 5 days after preparation. Actin filaments (red), nuclei (blue), and HUVECs (CD31, green/yellow). Scale bars 250 μm (bottom panel). Adapted with permission from ref (131). Copyright 2020 The Royal Society of Chemistry. (F) Schematic illustration of cancer cells behavior in multilayered micropassage-embedding composite hydrogel microfibers (left panel) and cross-section of the microfiber (middle panel). A549 cancer cells cocultured with NIH-3T3 fibroblasts in micropassage-hydrogel microfibers at day 0 and day 7. A549 cells were stained with red fluorescent dye (right panel). Adapted with permission from ref (206). Copyright 2018 The Royal Society of Chemistry.

Recently, Lu and co-workers^191^ demonstrated that confinement can also lead to altered gene expression and transformation of tumor cells. Similar to the work by Yu et al.,^184^ the approach relied on the use of core–shell hydrogel beads to impose physical confinement. The authors reported malignant transformation of the mammary epithelial cells upon culture, resulting in the transformation of healthy breast tissue into cancer-like tissue. MCF-10A cells, constituting a benign mammary cell line, were encapsulated in the Matrigel-core alginate-shell beads and analyzed after 3 weeks of culture for tumorigenicity. The encapsulated cells exhibited tumor-like behavior, including uncontrolled growth and formation of carcinomas in vitro and in vivo in immunocompromised mice (Figure 32A). On the contrary, MCF-10A cells embedded in bulk Matrigel formed growth-arrested cellular aggregates, suggesting that physical confinement may indeed induce malignant transformation in mammary epithelial cells. These results were supported by gene expression analysis, showing that 8110 genes were differentially expressed in confined cells in microcapsules as compared with cells cultured in bulk Matrigel. The system was accordingly proposed as a platform for high throughput testing of new breast cancer therapeutics.

Hydrogel microcapsules of compartmentalized topology provide a unique tool to study heterotypic cell–cell interactions in tumor microenvironment and to investigate cancer cell invasiveness. To address this issue, a one-step, multifluidic electrostatic spraying technique was developed, allowing for high throughput generation (>10 000 beads/min) of spherical beads of various microgel composition and topology, including double- and triple-layer core–shell and Janus microbeads (Figure 32B).^75^ The authors used the system to model breast cancer tissue by encapsulating MDA-MB-231 cancer cell, fibroblasts, and MCF-10A mammary cell line (representing healthy breast tissue) in separate compartments. The triple-layered capsules seemed to accurately mimic human breast cancer tissue, yet functional studies were not demonstrated.

A separate group of microstructures constitute hydrogel microcapsules dedicated for studies using patients’ material^20^ as well as in-depth studies of cancer biology.^374^ Jiang et al.^20^ combined droplet microfluidics and bioprinting to develop an automated organoid platform for studying interorganoid heterogeneity and heterogenic response to drugs in cancer patients. Cells of interest were encapsulated in Matrigel droplets and sequentially delivered into wells using a synchronized microfluidic droplet printer (Figure 32C). The platform was then used to perform differential gene expression analysis including studies of (i) healthy and tumor organoids, (ii) organoids and their parental tissues/tumors, and (iii) interpatient heterogeneous responses to anticancer drugs. The organoids recapitulated gene mutations in the parental tumor tissues and reflected the patient-to-patient variation in drug response and sensitivity, thus showing a tremendous potential of the automated organoid platform in the development of personalized anticancer therapies.

In another study, Wheeler et al.^374^ used digital microfluidic microgel system to recapitulate the breast cancer tumor microenvironment. Microgels mimicking mammary gland tissue were formed with a collagen I core surrounded by an encapsulating shell of basement membrane extract. To generate core–shell hydrogel structures, the droplets of collagen I and basal membrane extract were manipulated in an automated manner by using a digital-microfluidic DropBot control system based on electrowetting. MDA-MB-231 breast cancer cells were suspended in culture media and seeded on the platform by dispensing the droplets. The droplets were positioned such that they touched one side of a designated microgel (Figure 32D). The device was then rotated, allowing cells to sediment on the microgel surface and subsequently cultured in the original orientation. The system was used to perform image-based analysis of MDA-MB-231 breast cancer cell invasion and to study the differences in gene expression between invaded and noninvaded cell fractions for MDA-MB-231 cells. To characterize the cells of different invasiveness, microgels were excised from the platform and, after flash-freezing, dissected into thin sections, which were subsequently digested to release the embedded cells for transcriptomic analysis. The system allowed for easy and precise observation of cells under confocal microscope and straightforward extraction of various populations of cells. A disadvantage of the system is the relatively small number of microgels on the platform, which limits its potential in high-throughput screening.

Hydrogel microcapsules, despite being highly effective in drug screening, often lack cross-scale heterogeneity of matrix properties and/or cellular composition and therefore do not fully reflect the structural and compositional complexity of tumors in vivo. Microfluidic-based tumor microtissues of higher dimensionality, including compartmentalized microfibers^206^ and 3D hydrogel structures^115,131,269^ aim to recapitulate the tumor environment more precisely, e.g., via incorporation of vasculature or spatially varying matrix properties dedicated for cancer cell migration and invasiveness studies. Vascularized tumor models^115,131^ (described in more detail in section 5.2.7) are usually based on the assembly of 3D structures from cancer cell-laden hydrogel beads by incorporating them, together with endothelial cells, in an external bulk hydrogel. After a few days in culture, the endothelial cells self-assemble into capillaries forming a vascular network, eventually resulting in the formation of a vascularized tumor tissue (Figure 32E). Such living structures were used to study the effect of tumor microenvironment on cancer cell growth and invasiveness, to investigate the efficiency of certain drugs, as well as implanted in vivo.^115^

Tumor cell migration and invasion play a critical role in tumor metastasis and are closely associated with the level of malignancy and constitute a dominant factor in cancer-related mortality. Therefore, the assessment of cancer cell motility is crucial for tumor characterization, the choice of treatment, and eventually patient prognosis. Nevertheless, despite great progress in microfluidic 3D cancer cell invasion models, quantitative evaluation of cancer cell invasiveness is still an open challenge. In particular, random direction of cancer cell migration and dispersion of cells inside the matrix limit the possibility of such quantitative assessment. To address these issues, Sugimoto and co-workers^206^ developed a new approach to precisely control the initial cell positions and direction of cell migration in a coculture system mimicking physiologically relevant tumor conditions (Figure 32F). Microfluidic device consisting of an array of micronozzles supplying a gelation channel was used to fabricate composite hydrogel fibers. The generated fibers were composed of external shell made of RGD-alginate with a longitudinal slit or “micropassage” opening to the inner core composed of RGD-alginate with PGA. A549 lung cancer cells were encapsulated in the core, whereas fibroblasts were seeded in the surrounding shell region. Cancer cells gradually proliferated in the core and migrated through the micropassage, guided by the low-stiffness PGA-doped alginate, eventually forming colonies outside of the microfiber. Quantitative analysis of the cancer cell invasion was performed by counting cancer cell colonies outside of the fiber. The system was used to investigate the efficacy of three therapeutics, cisplatin, paclitaxel, and 5-fluorouracil, based on cancer cell invasion and metastasis. As expected, all of the drugs decreased the number of cancer cell colonies that migrated through the micropassage, suggesting that the system could be used for assessment of cell invasion in coculture conditions and for drug screening. Additionally, because the growth of the cancer cells was directed into the micropassage, the system did not require 3D confocal imaging, but only 2D fluorescence imaging, which made it more accessible.

In summary, during the past decade, microfluidic-based tumor microtissues have emerged as a promising and powerful tool in cancer research. Current technologies allow for precise 3D modeling of cancer biology, including studies on cancer metastasis and angiogenesis, as well as development of anticancer drug therapies. Furthermore, due to high sensitivity, high throughput, low material-consumption, and low cost, microfluidic-based cancer microtissues appear as a feasible tool in point-of-care diagnostics and personalized cancer treatment. In fact, pathologies like cancer, due to heterogeneous genetic and epigenetic alterations across individuals, often require patient-specific therapies. Present technologies mostly rely on the use of immortalized cancer cell lines, which have a great potential in high throughput anticancer drug screening studies but may lack the complexity characteristic of patient samples. Therefore, future studies should focus on reoptimization and redesigning of available approaches toward incorporation of patients’ material.

## 6 Challenges and Future Directions

Despite tremendous progress in recent years, microfluidics-assisted tissue engineering faces important challenges which must be resolved on a way toward its successful industrial and clinical applications.

First, generation of internally structured droplets or jets typically requires rather sophisticated microfluidic channel/nozzle designs whose preparation involves microfabrication, whereas fluid supply typically relies on the use of syringe pumps or pressure controllers. Altogether, generation of cell-laden microgels, as compared to other liquid/hydrogel handling techniques typically applied in a biological laboratory, e.g., manual pipetting, can be considered complex, technically involved, and relatively expensive. Such practical issues may be a barrier for widespread use of microfluidics in biomedical research. Accordingly, there is an urgent need for simple “workaround” solutions, e.g., microfluidic devices based on readily available components such as off-the-shelf connectors and tubings, with fluid supply driven simply by gravity or centrifugal forces. In fact, the use of a tabletop laboratory centrifuge in generation of alginate microbeads was already established nearly a decade ago.^62^ However, the method requires relatively high alginate concentrations (3% or more) in the precursor solution in order to provide monodisperse microbeads, which then leads to rather stiff microgels, not well suited for 3D cell culture. A solution to this problem is mixing of alginate with ECM-like hydrogels followed by reinforcing the microbeads with poly-l-lysine coating.^176^ Such prepared microgels have been used to encapsulate and culture cells for several days, during which cell adhesion and microtissue formation was observed. Yet, it is noteworthy that the monodispersity of the beads (CV ∼ 10%) was below typical microfluidic standards. More uniform centrifugation-based microbeads (CV < 5%) have been demonstrated with a custom-made spinning-disc system; despite overall excellent performance, such improvement in quality came at a price of a more involved experimental setup. Overall, further advancements in the development of point-of-care systems capable of operating “at the bedside”, e.g., encapsulating cells coming directly from patient biopsies without the use of bulky and/or expensive equipment, are urgently needed.

To allow high-throughput screening applications, the throughput of microgel generation in microfluidic devices should be further upscaled. In this respect, the methods relying on the use of piezo transducers^303^ or centrifugal forces^176^ are arguably among the most promising approaches. However, in systems generating hydrogel droplets at ultrahigh-throughput (>10^4^ Hz), the presence of cells at high densities tends to impact monodispersity of the droplets.^176,199^ Accordingly, further studies, including investigation of the possibility of parallelization of droplet generators,^351,379^ are required in order to optimize the operation of the microfluidic devices at cell concentrations of the order 10^6^–10^7^ cells/mL, typically required intissue-engineering applications.

From the point of view of basic tissue biology/physiology research, the organoids, including the microgel-based microtissues, constitute a unique playground for investigation of cell–cell interactions, cell–ECM interactions, as well as of the role of spatial cell segregation in tissue development.^88^ In this respect, the viability of the microtissues seems to depend on the type of cultured cells rather than on the type of the applied 3D culture method (e.g., microgel vs spheroid). In particular, the viability of fibroblasts such as HDF or endothelial cells such as HUVECs is typically limited to several weeks in vitro, whereas, for example, primary neurons or organoids differentiated from pluripotent stem cells^88^ tend to survive and maintain functionality for months.^417^ The most recent exciting developments in brain-organoid engineering^417,418^ demonstrate the importance of extended culture times (even months) to allow the development of complex tissues with functional organ-like subunits.

In regenerative medicine and cell therapeutic applications, the biological stability of the implanted cells, in particular their nontumorigenicity, is of paramount importance. This requirement remains in contrast with the majority of the currently available proof-of-concept microfluidic studies, which typically rely on encapsulation of immortalized cell lines, of which many, such as hepatocyte cell lines (HepG2, HepaRG) or beta cell lines (1.1B4), are in fact tumorigenic. Therefore, future demonstrations should focus on the use of predifferentiated stem cells, preferably derived from human induced pluripotent stem cells (hiPSC). Pancreatic, hepatic, and brain organoids based on hiPSC-laden microgels have already been demonstrated.^88,217,275^ For example, pancreatic fibers have been shown to suppress levels of glucose for up to half a year after implantation in diabetic mice,^51^ which raises hopes for future diabetes treatments based on hiPSCs.

Apart from cell tumorigenicity, hydrogel stability also remains one of the central issues in microtissue engineering. In particular, the long-term stability is advantageous in therapies in which the implanted cells must remain physically isolated from the hosts’ immune system in order to prevent an immediate immune response. In such applications, the encapsulated cells are actually supposed to produce the biologic cure (such as a hormones, cytokines, vesicles, etc.) rather than to proliferate and develop into a fully functional tissue. In contrast, in tissue-regenerative approaches, such as in muscle, tendon, or bone tissue restoration, the ultimate goal is the integration of an implant with the native tissue, including development of functional vascular and neuronal circuits. In such cases, the required hydrogel stability is midterm: it should support the cells directly post-transplantation but eventually get degraded upon tissue maturation. Whereas the long-term-stable microgels can be readily generated, e.g., with the use of alginate, the design of midterm-stable, fully biocompatible microgels still remains a challenge. For now, most of the approaches rely on the use of mixed alginate–ECM core–shell structures, which, despite promising results in vitro, may lead to side effects in vivo associated with the use of non-fully degradable biomaterials. Accordingly, synthesis of new types of microgels, fulfilling the requirements of stability and biodegradability remains an urgent challenge.

An interesting research perspective, directly associated with the modularity of the microgels, is the generation of the mesoscale structures consisting of tens to hundreds of compartments which could serve as scaffolds for *mesotissue engineering* (rather than microtissue). The basic challenge associated with such mesoscale modular/granular approach is the balance between the stability of the assembled mesostructure and reconfigurability of the building blocks, where the reconfigurability is required for efficient bottom-up assembly. More specifically, upon the assembly, the granular structure should allow rearrangements of the building blocks under the external driving forces, whereas after the assembly, the structure should not sponaneously rearrange but rather remain permanently arrested, warranting mechanical stability. The control over the associated granular “fluid–solid” transition can be achieved via fine-tuning of microgel properties^43^ such as particle size, volume fraction, as well as microgel–microgel interactions.^419^ Such control allows precise positioning of the individual compartments within much bigger constructs. First step in this direction has already been undertaken by the Bayley group.^88,366,419^ In particular, Alcinesio et al. demonstrated 3D printing of structures consisting of adhesive cell-laden hydrogel droplets suspended in oil and stabilized by lipids acting as surfactants.^419^ The droplet–droplet interactions, fine-tuned via changing the oil composition and/or lipid concentration, have been shown to impact the extent of droplet “annealing” (the droplet–droplet contact area) and led to either (i) unstable, (ii) stable amorphous, or (iii) stable ordered droplet arrangements. Exploiting the latter condition allowed 3D printing of hundreds of droplets at a single-droplet resolution.

From the bioengineering perspective, the bigger structures could allow to integrate various types of cells into organ-like constructs with a functional vasculature^115^ and/or innervation,^88^ which eventually could replace animal models and allow for more reliable, cost-effective, and ethical drug screening. The currently available biofabrication methods, such as the above-mentioned droplet 3D printing, still suffer from a relatively low throughput. However, a future combination of the granular self-assembly with rapid droplet generation using microfluidics, e.g., via the use of the double-emulsion-like approaches,^337,338,420^ could increase the throughputs up to the levels allowing applications in drug testing.

Finally, we shortly review the currently available commercial applications of the microfluidic-assembled microtissues. Considering the drug-testing applications, the U.S.-based company Xilis, founded in 2019, offers a product named MicroOrganoSphere (MOS) aimed at drug development and discovery as well as “drug prioritization” assays for patients based on microfluidic-encapsulated patient-derived microtumors. The product is currently under clinical trials for assessing advanced-stage colorectal and breast cancer tumors. Considering stem cell therapies, the company TreeFrog Therapeutics, established in 2018 in France, offers large-scale expansion of stem cells in the form of microtissues with the use of microfluidic core–shell encapsulation technology. Finally, the company Cellfiber in Japan commercializes alginate fiber technologies in a wide range of areas including drug development, food production, and bioremediation. Overall, as compared to the variety of general microfluidic companies (>260 startup companies worldwide^421^), including those developing organ-on-chip devices (around 30 companies worldwide as of 2017^422^), the market associated with microtissues can be considered as just emerging. Nevertheless, considering the remarkable potential of elevating the throughput and reducing the cost of drug testing, the technology can be expected to strongly impact, if not revolutionize, the pharmaceutical industry in the future as well as expedite the development of new strategies of personalized treatment of various diseases, including cancer.
